# Transition-metal-free approach to quinolines *via* direct oxidative cyclocondensation reaction of *N*,*N*-dimethyl enaminones with *o*-aminobenzyl alcohols

**DOI:** 10.3389/fchem.2022.1008568

**Published:** 2022-09-21

**Authors:** Kairui Rao, Zhangmengjie Chai, Pan Zhou, Donghan Liu, Yulin Sun, Fuchao Yu

**Affiliations:** Faculty of Life Science and Technology, Kunming University of Science and Technology, Kunming, China

**Keywords:** quinolines, N, N-dimethyl enaminones, o-aminobenzyl alcohols, oxidative cyclocondensation reaction, transition-metal-free

## Abstract

A transition-metal-free method for the construction of 3-substituted or 3,4-disubstituted quinolines from readily available *N*,*N*-dimethyl enaminones and *o*-aminobenzyl alcohols is reported. The direct oxidative cyclocondensation reaction tolerates broad functional groups, allowing the efficient synthesis of various quinolines in moderate to excellent yields. The reaction involves a C (sp^3^)-O bond cleavage and a C=N bind and a C=C bond formation during the oxidative cyclization process, and the mechanism was proposed.

## Introduction

Quinolines represent an important class of heterocyclic compounds, which widely occur as a core structural motif in natural products (McCormick et al., 1996; Subbaraju et al., 2004; McCauley et al., 2020), pharmaceuticals (Gorka et al., 2013; Kokatla et al., 2013; Jentsch et al., 2018), functional materials (Tong et al., 2003; Kim et al., 2005; Zhang et al., 2014), organocatalysis or ligands (Biddle et al., 2007; Zhang and Sigman., 2007; Esteruelas et al., 2016), and valuable building blocks (Wan et al., 2016; Duan et al., 2018; Wang et al., 2019; Ankade et al., 2021). Due to their great importance, considerable efforts have been focused on the development of efficient synthetic methods to their structures and modifications over the past years. Classical methodologies (Bharate et al., 2015; Li et al., 2017; Harry et al., 2020), such as Camps, Combes, Conrad–Limpach, Doebner, Friedländer, Knorr, Pfitzinger, Pavorov, Skraup synthesis, and others, are known for the construction of quinoline rings; however, these reactions usually suffer from some limitations, such as harsh reaction conditions, tedious workup procedures, and special substrate designs (prefunctionalized anilines). Recently, many elegant strategies toward quinolone rings, such as using new building blocks (Jin et al., 2016; Tiwari et al., 2017; Wu et al., 2017; Trofimov et al., 2018) and multicomponent reactions (Chen et al., 2018; Wang et al., 2018; Zhao et al., 2019; Yang and Wan., 2020), have been developed to construct substituted quinolines. Despite these advances, the development of easy and efficient approaches for the construction of substituted quinolines remains to be explored.

**SCHEME 1:**
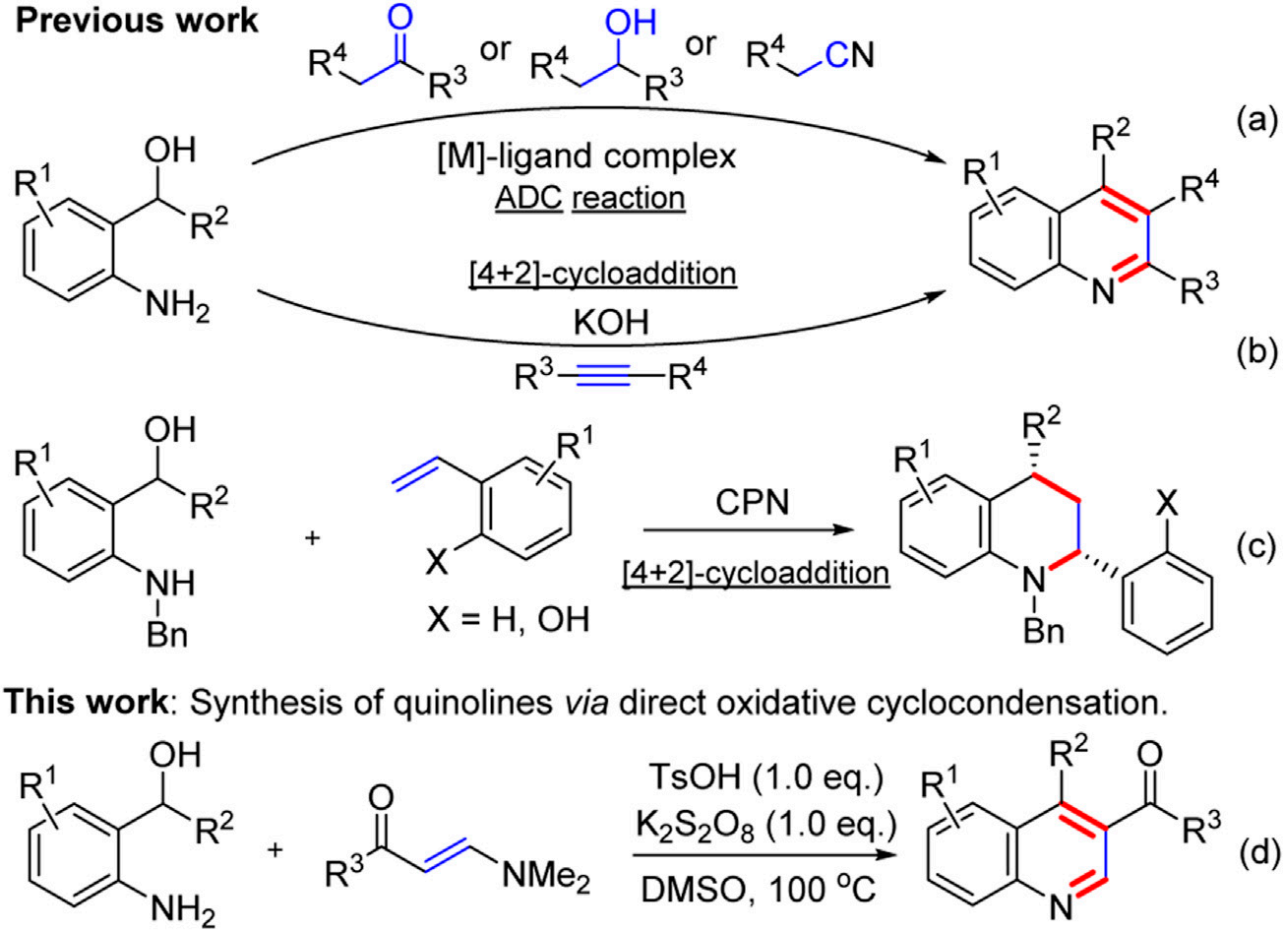
Synthesis of quinolines from *o*-aminobenzyl alcohols.

Recently, *o*-aminobenzyl alcohols are versatile intermediates which have attracted increasing attention in organic synthesis owing to their high reactivity in the construction of *N*-heterocycles (Makarov et al., 2018; Wang et al., 2018; Xie et al., 2018; Yang and Gao., 2018), especially quinolines. In this regard, two strategies have been developed to construct the quinoline framework from *o*-aminobenzyl alcohols: acceptorless dehydrogenative coupling (ADC) reactions and [4 + 2]-cycloaddition reactions. The types of ADC reactions of *o*-aminobenzyl alcohols with ketones or secondary alcohols or nitriles to the construction of quinolines by the release of H_2_ and H_2_O as only by-products have been well-developed (Scheme 1). However, such attractive synthetic strategies required expensive transition-metal (TM) pincer complexes, such as Ir (Wang et al., 2016; Genc et al., 2019), Ru (Maji et al., 2018; Wan et al., 2019), Ni (Das et al., 2018; Das et al., 2018; Singh et al., 2018), Mn (Mastalir et al., 2016; Barman et al., 2018; Das et al., 2019), Cu (Tan et al., 2018), or Re (Wei et al., 2019) complexes. In addition, aza-*ortho*-quinone methides (aza-*o*-QMs), *in situ* generated from *o*-aminobenzyl alcohols as short-lived and highly reactive diene species, have been extensively investigated and applied in organic synthesis (Huang and Kang., 2017; Mei et al., 2017; Lee et al., 2019; Wang et al., 2021). In 2016, a KOH-promoted regioselective synthesis of quinolones *via* [4 + 2]-cycloaddition of aza-*o*-QMs with internal alkynes was disclosed by Verma and co-workers (Saunthwal et al., 2016) (Scheme 1b). In 2018, Shi and co-workers established chiral phosphoramide catalytic asymmetric [4 + 2]-cycloaddition of aza-*o*-QMs with *o*-hydroxystyrenes to afford chiral tetrahydroquinolines (Li et al., 2018) (Scheme 1c). This [4 + 2]-cycloaddition protocol enriched the partners of aza-*o*-QMs to construct quinolones. In spite of these powerful works, there is still a demand for new protocols for generation of quinolines from *o*-aminobenzyl alcohols. As our ongoing interest in quinoline synthesis (Lu et al., 2017; Zhou et al., 2018) and enaminone chemistry (Yu et al., 2011; Yu et al., 2013; Xu et al., 2016; Zhou et al., 2017; Fu et al., 2020; Chen et al., 2021; Huang and Yu., 2021; Yu et al., 2021; Zhang et al., 2021; Fu et al., 2022; Liu et al., 2022; Ying et al., 2022), herein, we report a transition-metal-free direct oxidative cyclocondensation strategy of *o*-aminobenzyl alcohols with *N*,*N*-dimethyl enaminones to synthesize 3-substituted or 3,4-disubstituted quinoline derivatives in moderate to excellent yields (Scheme 1d).

## Results and discussion

Our investigation started with the reaction of readily available *N*,*N*-dimethyl enaminone **1a** with *o*-aminobenzyl alcohol **2a** as model substrates in (Table 1). We carried out the model reaction in the presence of AcOH in DMSO at 100°C, but the desired product **3a** was not obtained (entry 1). Various acids were screened, such as pivalic acid (PivOH), ZnCl_2_, trifluoroacetic acid (TFA), 10-camphorsulfonic acid (CSA), and *p*-toluenesulfonic acid (TsOH), which suggested that TsOH was the most suitable acid for this reaction in 32% yield. A series of oxidants show positive effects for the reaction (entries 7–13). To our delight, K_2_S_2_O_8_ was found to be the most effective one to give the desired quinolone **3a** for greatly increasing the yield to 82% (entry 13). Further experiments showed that DMSO was the first choice for solvents; other solvents, such as DMF, toluene, MeCN, 1,4-dioxane, EtOH, and water, were inferior (entries 14–19). With respect to the acid and oxidant loading, 1.0 equiv of TsOH and 1.0 equiv of K_2_S_2_O_8_ were found to be optimal (entries 20–23). The reaction temperature was also screened, and the results showed that 100°C was still with giving the best yield (entries 24–25).

**TABLE 1:** Optimization of the reaction conditions.^a^ ^,^ ^b^

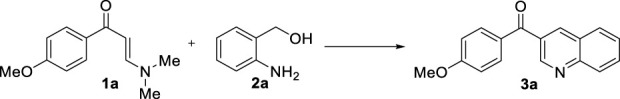					
Entry	Acid [eq.]	Oxidant [eq.]	Solvent	*T* [^o^C]	Yield [%]^b^
1	AcOH (1.0)		DMSO	100	n.d^c^
2	PivOH (1.0)		DMSO	100	n.d^c^
3	ZnCl_2_ (1.0)		DMSO	100	n.d^c^
4	TFA (1.0)		DMSO	100	25
5	CSA (1.0)		DMSO	100	15
6	TsOH (1.0)		DMSO	100	32
7	TsOH (1.0)	Oxone (1.0)	DMSO	100	68
8	TsOH (1.0)	TBHP (1.0)	DMSO	100	37
9	TsOH (1.0)	Fe_2_O_3_ (1.0)	DMSO	100	46
10	TsOH (1.0)	AgNO_3_ (1.0)	DMSO	100	59
11	TsOH (1.0)	DDQ (1.0)	DMSO	100	32
12	TsOH (1.0)	*m*-CPBA (1.0)	DMSO	100	40
**13**	**TsOH (1.0)**	**K**_**2**_ **S**_**2**_ **O**_**8**_ **(1.0)**	**DMSO**	**100**	**82**
14	TsOH (1.0)	K_2_S_2_O_8_ (1.0)	DMF	100	53
15	TsOH (1.0)	K_2_S_2_O_8_ (1.0)	Toluene	100	27
16	TsOH (1.0)	K_2_S_2_O_8_ (1.0)	MeCN	reflux	58
17	TsOH (1.0)	K_2_S_2_O_8_ (1.0)	1,4-Dioxane	100	38
18	TsOH (1.0)	K_2_S_2_O_8_ (1.0)	EtOH	reflux	62
19	TsOH (1.0)	K_2_S_2_O_8_ (1.0)	H_2_O	100	59
20	TsOH (0.5)	K_2_S_2_O_8_ (1.0)	DMSO	100	54
21	TsOH (1.5)	K_2_S_2_O_8_ (1.0)	DMSO	100	79
22	TsOH (1.0)	K_2_S_2_O_8_ (0.5)	DMSO	100	51
23	TsOH (1.0)	K_2_S_2_O_8_ (1.5)	DMSO	100	81
24	TsOH (1.0)	K_2_S_2_O_8_ (1.0)	DMSO	80	28
25	TsOH (1.0)	K_2_S_2_O_8_ (1.0)	DMSO	120	67

The bold values is designed to highlight the optimal reaction conditions.

a Reaction conditions: **1a** (0.5 mmol) and **2a** (0.5 mmol) in 3.0 ml solvent for 1.0 h.

b Isolated yields.

c Not detected.

**FIGURE 1:**
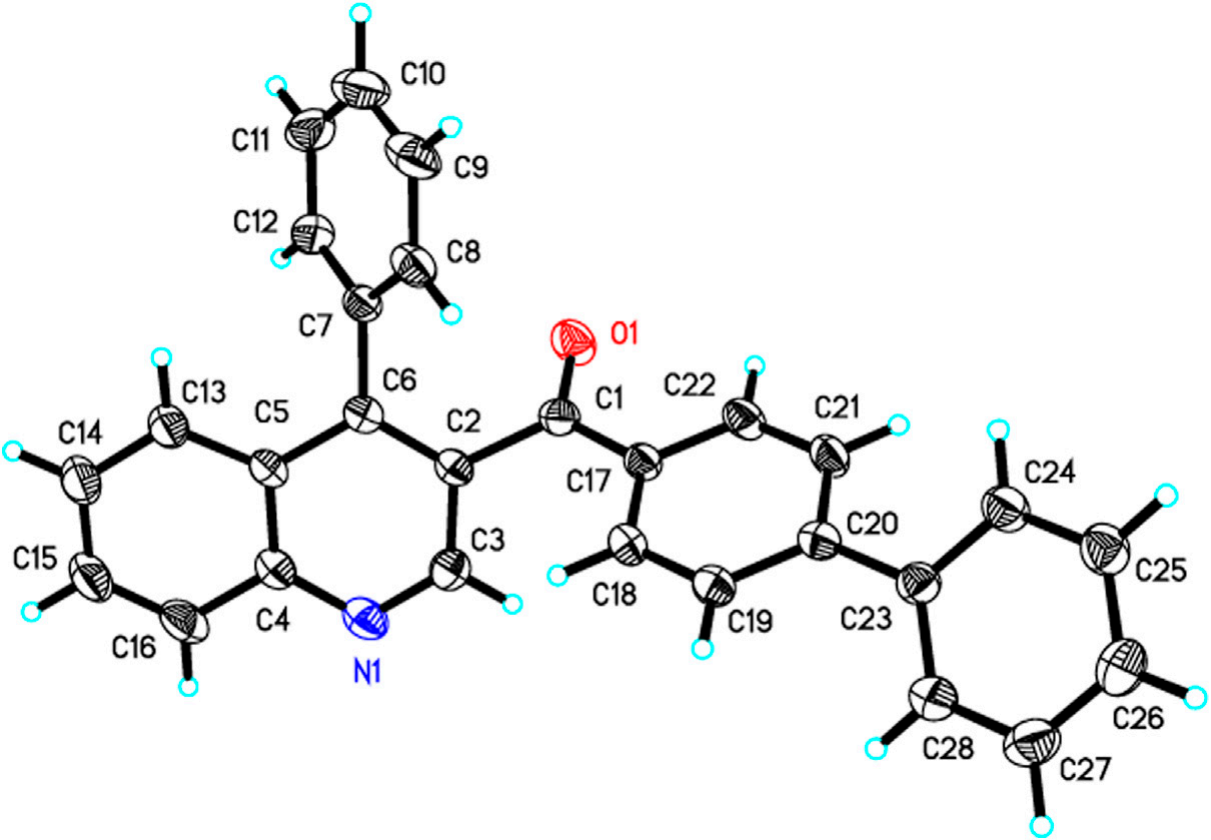
X-ray diffraction structure of 3j.

Under the optimized reaction conditions, we next investigated the substrate scope of this direct oxidative cyclocondensation reaction (Table 2). A wide range of *N*,*N*-dimethyl enaminones **1** bearing different substituents could be used in this transformation. For example, *N*,*N*-dimethyl enaminones bearing electron-rich (4-OMe, 4-Me, and 2-Me), electron-neutral (4-H), halogenated (4-Cl, 2-Cl, and 4-F), and electron-deficient (4-CF_3_ and 4-NO_2_) groups at the aryl ring were tolerated, affording the corresponding 3-substituted quinoline products in good to excellent yields (71–84%, **3a**−**3i**). Subsequently, 4-biphenyl and 1-naphthyl *N*,*N*-dimethyl enaminones were also well compatible with the reaction, giving the expected product in excellent yields (81–84%, **3j**−**3k**). Furthermore, various heteroaryl *N*,*N*-dimethyl enaminones, including 4-pyridyl, 2-furanyl, and 2-thienyl, were well tolerated in this reaction, affording the corresponding products in excellent yields (83–87%, **3l**−**3n**). The phenylethyl enamamine worked well for the reaction, furnishing the corresponding quinoline product **3o** in 61% yield. The *o*-aminobenzyl alcohol scope was also examined. Bearing halogenation (5-Cl) was well tolerated on the phenyl ring of the *o*-aminobenzyl alcohols, furnishing the corresponding 3-substituted quinoline products in good to excellent yields (78–89%, **3p**−**3x**). Notably, 1-(*o*-aminobenzyl) ethanol and *o*-aminobenzhydrol were also employed, affording 3,4-disubstituted quinolines in moderate to excellent yields (68–91%, **3y**−**3c’**). Moreover, the structure of **3j** was unambiguously confirmed by X-ray crystallographic analysis (CCDC 1846910, Figure 1).

**TABLE 2:** Scope of substrates.^a^ ^,^ ^b^

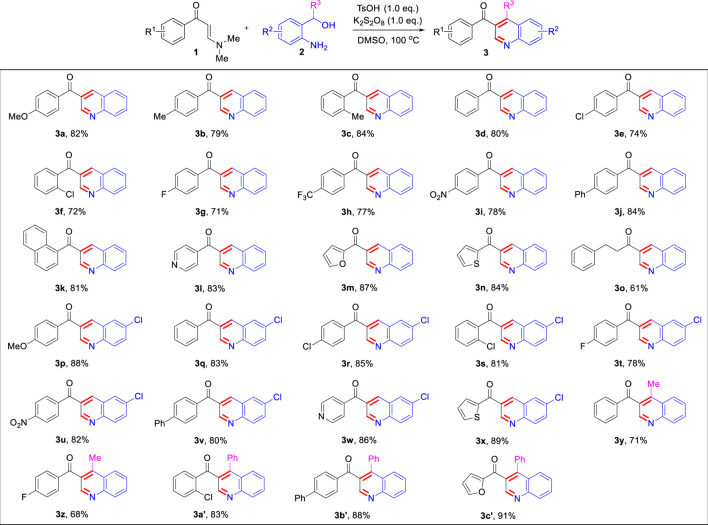

a Reaction conditions: *N*,*N*-dimethylenaminones **1** (0.5 mmol), aryl methyl ketones **2** (0.5 mmol), TsOH (0.5 mmol), and K_2_S_2_O_8_ (0.5 mmol) in 3.0 ml DMSO at 100 C for 1.0 h.

b Isolated yields.

**SCHEME 2:**
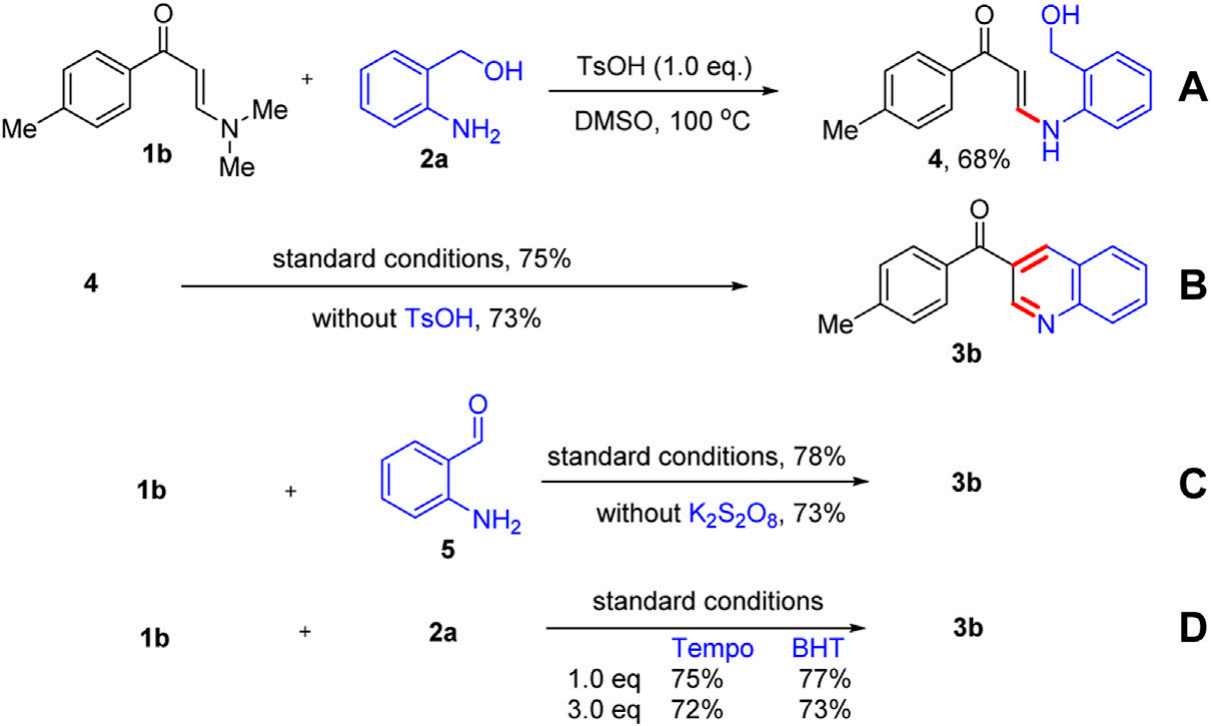
Control experiments.

To further understand the reaction mechanism, some control experiments were carried out, and the results are presented in Scheme 2. When *N*,*N*-dimethyl enaminone **1b** was reacted with *o*-aminobenzyl alcohol **2a** in the absence of K_2_S_2_O_8_, the *N*-aryl enaminone intermediate product **4** was obtained in 68% yield by a transamination process (Scheme 2). Next, product **3b** was obtained in 75% yield by the intramolecular cyclization reaction of intermediate **4** under optimized reaction conditions. However, the intramolecular cyclization reaction could also proceed smoothly without the addition of TsOH, affording product **3b** in 73% yield (Scheme 2). When *N*,*N*-dimethyl enaminone **1b** was reacted with 2-aminobenzaldehyde **5** under the standard conditions or in the absence of K_2_S_2_O_8_, product **3b** was, respectively, isolated in 78 and 73% yields (Scheme 2). Additionally, the reaction was unaffected completely by adding the radical inhibitors Tempo and BHT (Scheme 2). These results revealed that *N*-aryl enaminone **4** and 2-aminobenzaldehyde **5** were important intermediates for this reaction, and the reaction was not a free-radical process.

**SCHEME 3:**
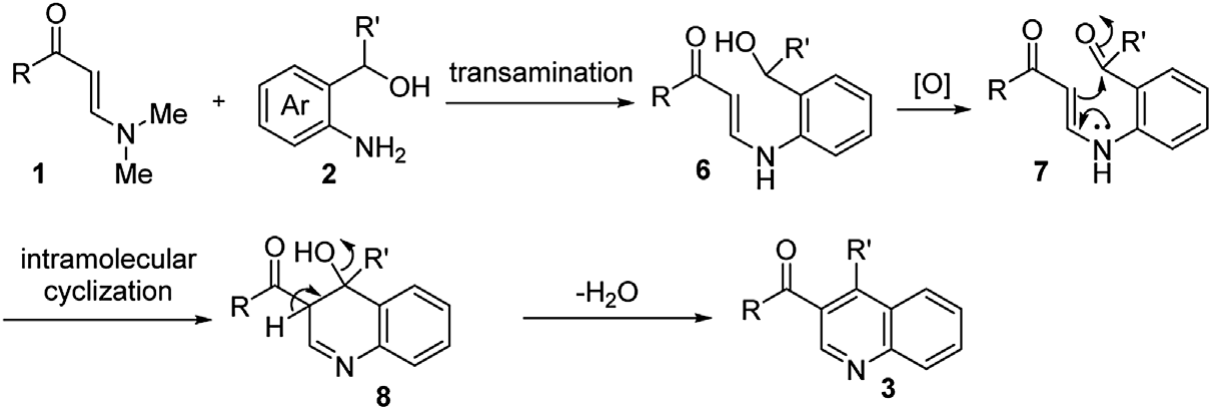
Proposed mechanism.

Based on the above results and previous studies (Zhou et al., 2018), a possible mechanism for this transformation is proposed (Scheme 3). *N*,*N*-dimethyl enaminones **1** reacted with *o*-aminobenzyl alcohols **2** promoted by TsOH to furnish the *N*-aryl enaminone intermediate **6** *via* a transamination process. Next, intermediate **6** underwent K_2_S_2_O_8_-assisted oxidation to form the ketone intermediate **7**, which was then converted into intermediate **8** through intramolecular cyclization reaction. Finally, quinolone products **3** were obtained *via* elimination of a molecule of H_2_O and oxidative aromatization.

## Conclusion

In conclusion, we have developed a concise protocol for the synthesis of 3-substituted or 3,4-disubstituted quinolines with moderate to excellent yields using readily available *N*,*N*-dimethyl enaminones and *o*-aminobenzyl alcohols promoted by TsOH/K_2_S_2_O_8_. This direct oxidative cyclocondensation reaction enriched the quinoline synthesis method from *o*-aminobenzyl alcohols.

## Data Availability

The original contributions presented in the study are included in the article/Supplementary Materials; further inquiries can be directed to the corresponding author.
