# Supplementary material for: Transition-metal-free approach to quinolines via direct oxidative cyclocondensation reaction of N,N-dimethyl enaminones with o-aminobenzyl alcohols
Source: Front Chem. 2022 Sep 21;10:1008568. doi: 10.3389/fchem.2022.1008568 (PMC9532769; doi:10.3389/fchem.2022.1008568)
Supplement: Supplementary file 1 [file DataSheet1.PDF]

Transition-Metal-Free Approach to quinolines *via* direct oxidative cyclocondensation reaction of *N,N*-dimethyl enaminones with *o*-aminobenzyl alcohols

Kairui Rao,[a] Zhangmengjie Chai,[a] Pan Zhou,[a] Donghan Liu,[a] Yulin Sun,[a] and Fuchao Yu*,[a]

[a] *Faculty of Life Science and Technology, Kunming University of Science and Technology, Kunming, 650500, P. R. China.*

Supporting Information

Table of Contents

1. General information. 2
2. General procedure for synthesis of 3-benzoyl quinolines 3. 3
3. General procedure for synthesis of intermediate product 4. 3
4. Spectroscopic data. 4
5. X-ray Structure and Data[1] of 3j (CCDC 1846910). 12
6. ^{1}H NMR and ^{13}C NMR spectra for spectroscopic data. 13
7. References and notes. 74

1. General information.

All compounds were fully characterized by spectroscopic data. The NMR spectra were recorded on a DRX600 (^{1}H: 600 MHz, ^{13}C: 150 MHz), chemical shifts (δ) are expressed in ppm, and J values are given in Hz, and deuterated $CDCl_3$ and DMSO-d_6 were used as solvent. The reactions were monitored by thin layer chromatography (TLC) using silica gel GF_{254}. The melting points were determined on XT-4A melting point apparatus and are uncorrected. HRMs were performed on an Agilent LC/MS TOF instrument.

All chemicals and solvents were used as received without further purification unless otherwise stated. Column chromatography was performed on silica gel (200–300 mesh).

Compounds **1** was prepared according to the literature[1]. The materials **2** were purchased from Adamas-beta®.

2. General procedure for synthesis of 3-benzoyl quinolines 3.

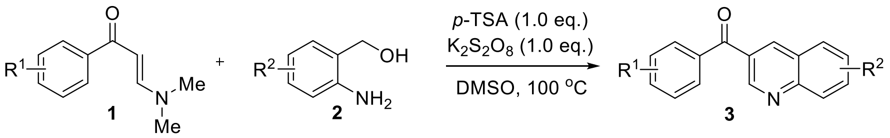

N,*N*-Dimethylenaminones **1** (0.5 mmol), 2-aminobenzylalcohols **2** (0.5 mmol), *p*-toluenesulfonic acid (TsOH) (0.5 mmol, 86 mg), $K_2S_2O_8$ (0.5 mmol, 135 mg), and DMSO (3.0 mL) were charged into a 10 mL Ace Glass pressure tubes, and the mixture was stirred at 100 °C for 1.0 h until *N*,*N*-dimethylenaminones **1** were completely consumed. The mixture was cooled to room temperature, concentrated under reduced pressure, and then EtOAc (15 mL × 2) were added, dried over anhydrous Na_2SO_4, concentrated and purified by flash column chromatography to afford 3-benzoyl quinolines **3**.

3. General procedure for synthesis of intermediate product 4.

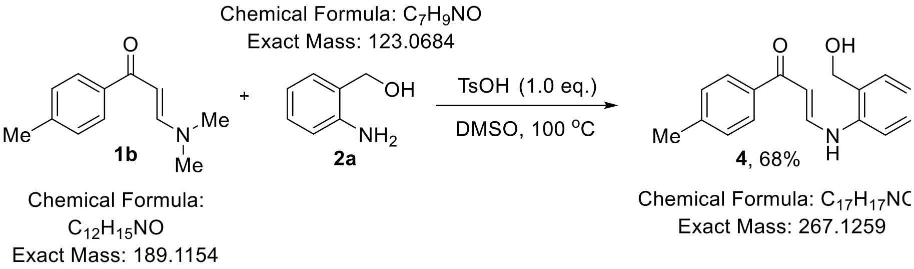

N,*N*-Dimethylenaminone **1b** (0.5 mmol, 95 mg), 2-aminobenzylalcohol **2a** (0.5 mmol, 62 mg), *p*-toluenesulfonic acid (TsOH) (0.5 mmol, 86 mg), $K_2S_2O_8$ (0.5 mmol, 135 mg), and DMSO (3.0 mL) were charged into a 10 mL Ace Glass pressure tubes, and the mixture was stirred at 100 °C for 1.0 h until *N*,*N*-dimethylenaminone **1b** were completely consumed. The mixture was cooled to room temperature, concentrated under reduced pressure, and then EtOAc (15 mL × 2) were added, dried over anhydrous Na_2SO_4, concentrated and purified by flash column chromatography to afford intermediate product **4**.

4. Spectroscopic data.

(4-Methoxyphenyl)(quinolin-3-yl)methanone (3a)

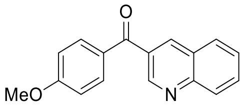

Yellow solid; Yield = 82%, 108 mg; mp 140–142 °C; ^{1}H NMR (600 MHz, $CDCl_3$): δ = 3.94 (s, 3H, $ArOCH_3$), 7.04 (d, 2H, J = 8.76 Hz, ArH), 7.65–7.68 (m, 1H, ArH), 7.86–7.96 (m, 4H, ArH), 7.72 (d, J = 8.46 Hz, 1H, ArH), 8.55 (d, J = 2.16 Hz, 1H, ArH), 9.30 (d, J = 2.10 Hz, 1H, ArH); ^{13}C NMR (150 MHz, $CDCl_3$): δ = 55.6, 114.0, 114.0, 126.7, 127.5, 129.1, 129.5, 129.7, 130.8, 131.6, 132.6, 132.6, 138.3, 149.3, 150.3, 163.7, 193.5; HRMS (TOF ES^{+}): m/z calcd for $C_{17}H_{14}NO_2^{+}$ [(M+H)$^{+}$], 264.1019; found, 264.1017.

Quinolin-3-yl(*p*-tolyl)methanone (3b)

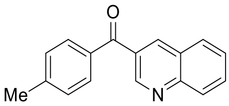

Yellow solid; Yield = 79%, 97 mg; mp 116–118 °C; ^{1}H NMR (600 MHz, $CDCl_3$): δ = 2.50 (s, 3H, $ArCH_3$), 7.36 (d, 2H, J = 7.44 Hz, ArH), 7.65–7.67 (m, 1H, ArH), 7.81 (d, J = 8.16 Hz, 2H, ArH), 7.86–7.89 (m, 1H, ArH), 7.94 (dd, J = 8.58 Hz, 1H, ArH), 8.22 (dd, J = 8.37 Hz, 1H, ArH), 8.57 (d, J = 1.38 Hz, 1H, ArH), 9.33 (d, J = 2.22 Hz, 1H, ArH); ^{13}C NMR (150 MHz, $CDCl_3$): δ = 21.8, 126.7, 127.6, 129.1, 129.4, 129.4, 129.5, 130.3, 130.3, 130.5, 131.7, 134.4, 138.6, 144.1, 149.4, 150.4, 194.6; HRMS (TOF ES^{+}): m/z calcd for $C_{17}H_{14}NO^{+}$ [(M+H)$^{+}$], 248.1070; found, 248.1068.

Quinolin-3-yl(*o*-tolyl)methanone (3c)

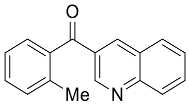

Brown solid; Yield = 84%, 104 mg; mp 77–79 °C; ^{1}H NMR (600 MHz, $CDCl_3$): δ = 2.43 (s, 3H, $ArCH_3$), 7.32–7.35 (m, 1H, ArH), 7.39–7.43 (m, 2H, ArH), 7.48–7.51 (m, 1H, ArH), 7.64–7.66 (m, 1H, ArH), 7.87–7.92 (m, 2H, ArH), 8.21 (d, J = 8.52 Hz, 1H, ArH), 8.51 (d, J = 8.22 Hz, 1H, ArH), 9.38 (d, J = 2.16 Hz, 1H, ArH); ^{13}C NMR (150 MHz, $CDCl_3$): δ = 20.2, 125.5, 126.7, 127.6, 128.9, 129.4, 129.5, 130.2, 131.0, 131.5, 132.2, 137.3, 137.5, 139.4, 149.7, 150.3, 197.0; HRMS (TOF ES^{+}): m/z calcd for $C_{17}H_{14}NO^{+}$ [(M+H)$^{+}$], 248.1070; found, 248.1070.

Phenyl(quinolin-3-yl)methanone (3d)

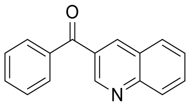

White solid; Yield = 80%, 93 mg; mp 74–76 °C; ^{1}H NMR (600 MHz, $CDCl_3$): δ = 7.56–7.59 (m, 2H, ArH), 7.66–7.70 (m, 2H, ArH), 7.87–7.91 (m, 3H, ArH), 7.95 (dd, J = 8.19 Hz, 1H, ArH), 8.22 (dd, J = 8.52 Hz, 1H, ArH), 8.59 (d, J = 2.16 Hz, 1H, ArH), 9.35 (d, J = 2.22 Hz, 1H, ArH); ^{13}C NMR (150 MHz, $CDCl_3$): δ = 126.6, 127.6, 128.7, 128.7, 129.2, 129.5, 130.1, 130.1, 130.1, 131.9, 133.1, 137.1, 138.1, 149.5, 150.4, 194.9; HRMS (TOF ES^{+}): m/z calcd for $C_{16}H_{12}NO^{+}$ [(M+H)$^{+}$], 234.0913; found,

234.0912.

(4-Chlorophenyl)(quinolin-3-yl)methanone (3e)

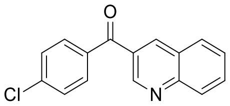

Yellow solid; Yield = 74%, 99 mg; mp 112–114 °C; ^{1}H NMR (600 MHz, $CDCl_3$): δ = 7.53 (d, 2H, J = 8.46 Hz, ArH), 7.65–7.67 (m, 1H, ArH), 7.83 (d, 2H, J = 8.52 Hz, ArH), 7.86–7.89 (m, 1H, ArH), 7.93 (d, J = 8.22 Hz, 1H, ArH), 8.20 (d, J = 8.46 Hz, 1H, ArH), 8.54 (d, J = 2.10 Hz, 1H, ArH), 9.30 (d, J = 2.22 Hz, 1H, ArH); ^{13}C NMR (150 MHz, $CDCl_3$): δ = 126.5, 127.7, 129.0, 129.0, 129.2, 129.6, 129.7, 131.4, 131.4, 132.0, 135.3, 138.7, 139.7, 149.6, 150.1, 193.7; HRMS (TOF ES^+): *m/z* calcd for $C_{16}H_{11}ClNO^+$ $[(M+H)^+]$,268.0524; found, 268.0520.

(2-Chlorophenyl)(quinolin-3-yl)methanone (3f)

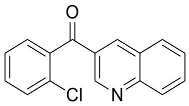

Yellow solid; Yield = 72%, 96 mg; mp 108–110 °C; ^{1}H NMR (600 MHz, $CDCl_3$): δ = 7.46–7.53 (m, 4H, ArH), 7.62–7.64 (m, 1H, ArH), 7.85–7.91 (m, 2H, ArH), 8.19 (dd, J = 8.43 Hz, 1H, ArH), 8.49 (d, J = 2.22 Hz, 1H, ArH), 9.35 (d, J = 2.22 Hz, 1H, ArH); ^{13}C NMR (150 MHz, $CDCl_3$): δ = 126.7, 127.0, 127.6, 128.9, 129.4, 129.5, 129.5, 130.3, 131.5, 131.8, 132.4, 137.6, 139.4, 149.9, 150.0, 194.0; HRMS (TOF ES^+): *m/z* calcd for $C_{16}H_{11}ClNO^+$ $[(M+H)^+]$, 268.0524; found, 268.0522.

(4-Fluorophenyl)(quinolin-3-yl)methanone (3g)

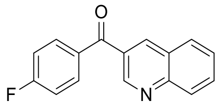

Yellow solid; Yield = 71%, 89 mg; mp 83–85 °C; ^{1}H NMR (600 MHz, $CDCl_3$): δ = 7.22–7.27 (m, 2H, ArH), 7.65–7.68 (m, 1H, ArH), 7.86–7.94 (m, 4H, ArH), 8.21 (d, J = 8.40 Hz, 1H, ArH), 8.54 (d, J = 2.16 Hz, 1H, ArH), 9.30 (d, J = 2.22 Hz, 1H, ArH); ^{13}C NMR (150 MHz, $CDCl_3$): δ = 116.9 (J = 22.5 Hz), 116.9 (J = 22.5 Hz), 126.6, 127.7, 129.1, 129.6, 130.0, 131.9, 132.7 (J = 9.0 Hz), 132.7 (J = 9.0 Hz), 133.3 (J = 2.97 Hz), 138.6, 149.5, 150.2, 165.7 (J = 253.91 Hz), 193.4; HRMS (TOF ES^+): *m/z* calcd for $C_{16}H_{11}FNO^+$ $[(M+H)^+]$, 252.0819; found, 252.0811.

Quinolin-3-yl(4-(trifluoromethyl)phenyl)methanone (3h)

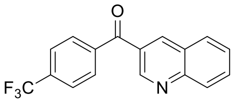

White solid; Yield = 77%, 116 mg; mp 110–112 °C; ^{1}H NMR (600 MHz, $CDCl_3$): δ = 7.67–7.71 (m, 1H, ArH), 7.85 (d, 2H, J = 8.10 Hz, ArH), 7.91–8.01 (m, 4H, ArH), 8.25 (d, J = 8.40 Hz, 1H, ArH), 8.58 (d, J = 2.10 Hz, 1H, ArH), 9.37 (d, J = 2.28 Hz, 1H, ArH); ^{13}C NMR (150 MHz, $CDCl_3$): δ = 122.7, 124.5, 125.8, 125.8, 126.5, 127.9, 129.3, 129.6, 130.2, 130.2, 132.3, 134.4 (J = 131.76 Hz), 139.1, 140.0, 149.7, 150.1, 193.9; HRMS (TOF ES^+): *m/z* calcd for $C_{17}H_{11}F_3NO^+$ $[(M+H)^+]$, 302.0787; found, 302.0787.

(4-Nitrophenyl)(quinolin-3-yl)methanone (3i)

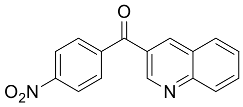

Yellow solid; Yield = 78%, 108 mg; mp 146–148 °C; ^{1}H NMR (600 MHz, $CDCl_3$): δ = 7.70–7.72 (m, 1H, ArH), 7.92–7.97 (m, 2H, ArH), 8.05 (d, J = 8.76 Hz, 2H, ArH), 8.24 (dd, J = 8.55 Hz, 1H, ArH), 8.43 (d, J = 8.76 Hz, 2H, ArH), 8.58 (d, J = 1.38 Hz, 1H, ArH), 9.36 (d, J = 2.22 Hz, 1H, ArH); ^{13}C NMR (150 MHz, $CDCl_3$): δ = 123.9, 123.9, 126.4, 128.0, 128.8, 129.3, 129.7, 130.8, 130.8, 132.6, 139.2, 142.1, 142.1, 149.9, 150.2, 193.2; HRMS (TOF ES$^+$): m/z calcd for $C_{16}H_{11}N_2O_3^+$ [(M+H)$^+$], 279.0764; found, 279.0763.

[1,1'-Biphenyl]-4-yl(quinolin-3-yl)methanone (3j)

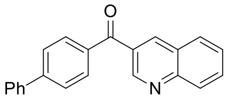

Yellow solid; Yield = 84%, 129 mg; mp 124–126 °C; ^{1}H NMR (600 MHz, $CDCl_3$): δ = 7.44–7.47 (m, 1H, ArH), 7.52–7.54 (m, 2H, ArH), 7.67–7.71 (m, 3H, ArH), 7.80 (d, J = 8.10 Hz, 2H, ArH), 7.83–7.91 (m, 1H, ArH), 7.97–8.00 (m, 3H, ArH), 8.24 (d, J = 8.46 Hz, 1H, ArH), 8.63 (d, J = 2.10 Hz, 1H, ArH), 9.39 (d, J = 2.10 Hz, 1H, ArH); ^{13}C NMR (150 MHz, $CDCl_3$): δ = 126.7, 127.3, 127.3, 127.4, 127.4, 127.6, 128.4, 129.1, 129.1, 129.2, 129.5, 130.3, 130.8, 130.8, 131.9, 135.7, 138.8, 139.7, 145.9, 149.5, 150.4, 194.5; HRMS (TOF ES$^+$): m/z calcd for C22H16NO+ [(M+H)$^+$], 310.1226; found, 310.1226.

Naphthalen-1-yl(quinolin-3-yl)methanone (3k)

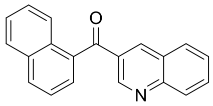

Brown solid; Yield = 81%, 115 mg; mp 101–103 °C; ^{1}H NMR (600 MHz, $CDCl_3$): δ = 7.56–7.67 (m, 4H, ArH), 7.69 (dd, J = 6.96 Hz, 1H, ArH), 7.87–7.90 (m, 2H, ArH), 8.00 (d, J = 7.86 Hz, 1H, ArH), 8.11 (d, J = 8.22 Hz, 1H, ArH), 8.22–8.24 (m, 2H, ArH), 8.57 (d, J = 2.10 Hz, 1H, ArH), 9.45 (d, J = 2.16 Hz, 1H, ArH); ^{13}C NMR (150 MHz, $CDCl_3$): δ = 124.4, 125.5, 126.7, 126.8, 127.6, 127.8, 128.5, 128.6, 129.4, 129.5, 130.8, 130.9, 132.2, 132.2, 133.9, 135.3, 139.7, 149.8, 150.5, 196.4; HRMS (TOF ES$^+$): m/z calcd for $C_{20}H_{14}NO^+$ [(M+H)$^+$], 284.1070; found, 284.1070.

Pyridin-4-yl(quinolin-3-yl)methanone (3l)

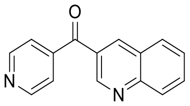

Yellow solid; Yield = 83%, 97 mg; mp 144–146 °C; ^{1}H NMR (600 MHz, $CDCl_3$): δ = 7.67–7.71 (m, 3H, ArH), 7.91–7.97 (m, 2H, ArH), 8.23 (d, J = 8.40 Hz, 1H, ArH), 8.58 (d, J = 2.16 Hz, 1H, ArH), 8.91 (d, J = 4.14 Hz, 2H, ArH), 9.37 (d, J = 2.16 Hz, 1H, ArH); ^{13}C NMR (150 MHz, $CDCl_3$): δ = 122.8, 126.4, 128.0, 128.4, 129.4, 129.6, 132.6, 139.4, 143.6, 149.9, 150.8, 150.8, 150.8, 193.7; HRMS (TOF ES$^+$): m/z calcd for $C_{15}H_{11}N_2O^+$ [(M+H)$^+$], 235.0866; found, 235.0866.

Furan-2-yl(quinolin-3-yl)methanone (3m)

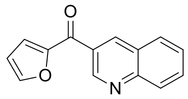

Yellow solid; Yield = 87%, 97 mg; mp 111–113 °C; ^{1}H NMR (600 MHz, $CDCl_3$): δ = 7.70–7.71 (m, 1H, C=CH), 7.42 (d, J = 3.66 Hz, 1H, ArH), 7.66–7.69 (m, 1H, C=CH), 7.80 (d, J = 1.56 Hz, 1H, C=CH), 7.87–7.90 (m, 1H, ArH), 8.00 (dd, J = 8.25 Hz, 1H, ArH), 8.21 (d, J = 8.40 Hz, 1H, ArH) , 8.87 (d, J = 2.10 Hz, 1H, ArH) , 9.45 (d, J = 2.10 Hz, 1H, ArH); ^{13}C NMR (150 MHz, $CDCl_3$): δ = 112.7, 120.8, 126.8, 127.6, 129.3, 129.5, 129.7, 131.9, 138.4, 147.5, 149.6, 149.8, 152.4, 180.4; HRMS (TOF ES$^+$): m/z calcd for $C_{14}H_{10}NO_2^+$ [(M+H)$^+$], 224.0706; found, 224.0705.

Quinolin-3-yl(thiophen-2-yl)methanone (3n)

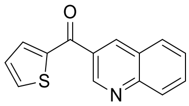

Yellow solid; Yield = 84%, 100 mg; mp 91–93 °C; ^{1}H NMR (600 MHz, $CDCl_3$): δ = 7.23–7.24 (m, 1H, C=CH), 7.65–7.68 (m, 1H, ArH), 7.73 (dd, J = 3.81 Hz, 1H, C=CH), 7.81 (dd, J = 4.92 Hz, 1H, C=CH), 7.85–7.88 (m, 1H, ArH), 7.96 (dd, J = 8.22 Hz, 1H, ArH), 8.21 (dd, J = 8.49 Hz, 1H, ArH) , 8.67 (d, J = 2.16 Hz, 1H, ArH) , 9.36 (d, J = 2.22 Hz, 1H, ArH); ^{13}C NMR (150 MHz, $CDCl_3$): δ = 126.0, 127.0, 127.6, 128.4, 128.8, 130.0, 131.1, 134.3, 134.4, 137.0, 142.5, 148.8, 148.9, 185.5; HRMS (TOF ES$^+$): m/z calcd for $C_{14}H_{10}NOS^+$ [(M+H)$^+$], 240.0478; found, 240.0475.

3-Phenyl-1-(quinolin-3-yl)propan-1-one (3o)

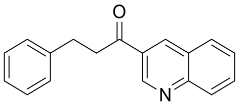

White solid; Yield = 61%, 80 mg; mp 111–113 °C; ^{1}H NMR (600 MHz, $CDCl_3$): δ = 3.15 (t, J = 7.50 Hz, 2H, C-CH_2), 3.45 (t, J = 7.60 Hz, 2H, C-CH_2), 7.24–7.22 (m, 1H, ArH), 7.34–7.29 (m, 4H, ArH), 7.63 (t, J = 7.40 Hz, 1H, ArH), 7.85 (t, J = 7.50 Hz, 1H, ArH), 7.93 (d, J = 8.00 Hz, 1H, ArH), 8.16 (d, J = 8.40 Hz, 1H, ArH), 8.70 (s, 1H, C=CH), 9.44 (s, 1H, C=CH); ^{13}C NMR (150 MHz, $CDCl_3$): δ = 29.9, 40.9, 126.4, 126.9, 127.6, 128.5, 128.5, 128.7, 128.7, 129.0, 129.4, 129.5, 132.1, 137.1, 140.9, 149.1, 149.8, 198.1; HRMS (TOF ES$^+$): m/z calcd for $C_{18}H_{16}NO^+$ [(M+H)$^+$], 262.1226; found, 262.1224.

(6-Chloroquinolin-3-yl)(4-methoxyphenyl)methanone (3p)

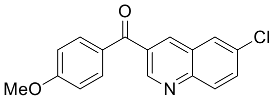

Yellow solid; Yield = 88%, 130 mg; mp 150–152 °C; ^{1}H NMR (600 MHz, $CDCl_3$): δ = 3.94 (s, 3H, $ArOCH_3$), 7.04 (d, 2H, J = 8.88 Hz, ArH), 7.80 (dd, 1H, J = 8.97 Hz, ArH), 7.90 (dd, 2H, J = 6.78 Hz, ArH), 7.93 (d, 1H, J = 2.52 Hz, ArH), 8.16 (d, 1H, J = 8.94 Hz, ArH), 8.45 (d, J = 2.04 Hz, 1H, ArH), 9.27 (d, J = 2.16 Hz, 1H, ArH); ^{13}C NMR (150 MHz, $CDCl_3$): δ = 55.6, 114.0, 114.0, 127.4,

127.5, 129.4, 131.1, 131.6, 132.4, 132.6, 132.6, 133.4, 137.1, 147.6, 150.5, 163.9, 193.1; HRMS (TOF ES$^+$): *m/z* calcd for $C_{17}H_{13}ClNO_2^+$ [(M+H)$^+$], 298.0629; found, 298.0629.

(6-Chloroquinolin-3-yl)(phenyl)methanone (3q)

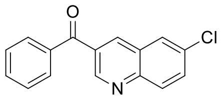

Yellow solid; Yield = 83%, 111 mg; mp 140–142 °C; ^{1}H NMR (600 MHz, CDCl$_3$): δ = 7.57–7.60 (m, 2H, ArH), 7.69–7.71 (m, 1H, ArH), 7.81 (dd, J = 8.97 Hz, 1H, ArH), 7.88–7.90 (m, 2H, ArH), 7.94 (d, J = 2.34 Hz, 1H, ArH), 8.17 (d, J = 9.00 Hz, 1H, ArH), 8.49 (dd, J = 2.22 Hz, 1H, ArH), 9.33 (d, J = 2.16 Hz, 1H, ArH); ^{13}C NMR (150 MHz, CDCl$_3$): δ = 127.3, 127.6, 128.8, 128.8, 130.1, 130.1, 130.8, 131.1, 132.7, 133.3, 133.5, 136.7, 137.7, 147.8, 150.5, 194.6; HRMS (TOF ES$^+$): *m/z* calcd for $C_{16}H_{11}ClNO^+$ [(M+H)$^+$], 268.0524; found, 268.0524.

(4-Chlorophenyl)(6-chloroquinolin-3-yl)methanone (3r)

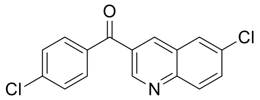

Yellow solid; Yield = 85%, 128 mg; mp 195–197 °C; ^{1}H NMR (600 MHz, DMSO-d_6+CDCl$_3$): δ = 7.64 (d, 2H, J = 8.34 Hz, ArH), 7.87–7.89 (m, 3H, ArH), 8.13 (d, 1H, J = 8.94 Hz, ArH), 8.26 (d, J = 2.40 Hz, 1H, ArH), 8.72 (d, J = 2.28 Hz, 1H, ArH), 9.19 (d, J = 2.28 Hz, 1H, ArH); ^{13}C NMR (150 MHz, DMSO-d_6+CDCl$_3$): δ = 127.5, 128.6, 129.3, 129.3, 130.5, 131.3, 132.1, 132.1, 132.6, 132.8, 135.5, 138.4, 138.9, 147.7, 150.4, 193.4; HRMS (TOF ES$^+$): *m/z* calcd for $C_{16}H_{10}Cl_2NO^+$ [(M+H)$^+$], 302.0134; found, 302.0134.

(2-Chlorophenyl)(6-chloroquinolin-3-yl)methanone (3s)

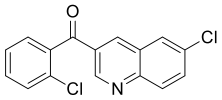

Yellow solid; Yield = 81%, 122 mg; mp 117–119 °C; ^{1}H NMR (600 MHz, CDCl$_3$): δ = 7.47–7.52 (m, 2H, ArH), 7.54–7.58 (m, 2H, ArH), 7.81 (dd, 1H, J = 9.00 Hz, ArH), 7.92 (d, J = 2.34 Hz, 1H, ArH), 8.15 (d, J = 8.94 Hz, 1H, ArH), 8.41 (d, J = 2.22 Hz, 1H, ArH), 9.35 (d, J = 2.10 Hz, 1H, ArH); ^{13}C NMR (150 MHz, CDCl$_3$): δ = 127.2, 127.5, 127.9, 129.5, 129.7, 130.4, 131.2, 131.5, 132.1, 133.2, 133.5, 137.4, 138.4, 148.3, 150.1, 193.7; HRMS (TOF ES$^+$): *m/z* calcd for $C_{16}H_{10}Cl_2NO^+$ [(M+H)$^+$], 302.0134; found, 302.0134.

(6-Chloroquinolin-3-yl)(4-fluorophenyl)methanone (3t)

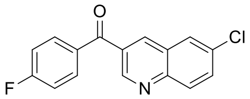

Yellow solid; Yield = 78%, 111 mg; mp 76–78 °C; ^{1}H NMR (600 MHz, CDCl$_3$): δ = 7.24–7.29 (m, 2H, ArH), 7.81–7.83 (m, 1H, ArH), 7.92–7.94 (m, 3H, ArH), 8.17 (d, J = 8.94 Hz, 1H, ArH), 8.46 (d, J = 2.04 Hz, 1H, ArH), 9.29 (d, J = 1.98 Hz, 1H, ArH); ^{13}C NMR (150 MHz, CDCl$_3$): δ = 116.0 (J = 21.0 Hz), 116.0 (J = 21.0 Hz), 127.3, 127.6, 130.8, 131.2, 132.7, 132.8 (J = 1.50 Hz), 132.8 (J = 1.50

Hz), 133.0 (J = 2.96 Hz), 133.6, 137.4, 147.8, 150.3, 162.9 (J = 254.48 Hz), 193.0; HRMS (TOF ES^+): m/z calcd for $C_{16}H_{10}ClFNO^+$ $[(M+H)^+]$, 286.0429; found, 286.0429.

(6-Chloroquinolin-3-yl)(4-nitrophenyl)methanone (3u)

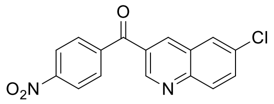

Yellow solid; Yield = 82%, 128 mg; mp 177–179 °C; ^{1}H NMR (600 MHz, $CDCl_3$): δ = 7.85 (dd, J = 9.00 Hz, 1H, ArH), 7.95 (d, J = 2.28 Hz, 1H, ArH), 8.04 (d, J = 8.70 Hz, 2H, ArH), 8.18 (d, J = 8.94 Hz, 1H, ArH), 8.44 (d, J = 2.76 Hz, 2H, ArH), 8.49 (d, J = 1.86 Hz, 1H, ArH), 9.33 (d, J = 2.22 Hz, 1H, ArH); ^{13}C NMR (150 MHz, $CDCl_3$): δ = 124.0, 124.0, 127.1, 127.7, 129.6, 130.8, 130.8, 131.2, 133.4, 134.0, 138.0, 141.7, 148.2, 150.0, 150.3, 192.9; HRMS (TOF ES^+): m/z calcd for $C_{16}H_{10}ClN_2O_3^+$ $[(M+H)^+]$, 313.0374; found, 313.0374.

[1,1'-Biphenyl]-4-yl(6-chloroquinolin-3-yl)methanone (3v)

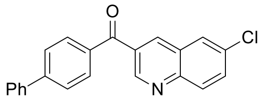

Yellow solid; Yield = 80%, 137 mg; mp 151–153 °C; ^{1}H NMR (600 MHz, $CDCl_3$): δ = 7.45–7.48 (m, 1H, ArH), 7.52–7.55 (m, 2H, ArH), 7.70 (dd, J = 8.19 Hz, 2H, ArH), 7.79–7.83 (m, 3H, ArH), 7.96–7.98 (m, 3H, ArH), 8.18 (d, J = 8.94 Hz, 1H, ArH), 8.53 (d, J = 4.26 Hz, 1H, ArH), 9.35 (d, J = 2.16 Hz, 1H, ArH); ^{13}C NMR (150 MHz, $CDCl_3$): δ = 127.3, 127.4, 127.4, 127.4, 127.4, 127.6, 128.5, 129.1, 129.1, 130.7, 130.7, 131.0, 131.1, 132.7, 133.5, 135.4, 137.6, 139.6, 146.2, 147.8, 150.5, 194.1; HRMS (TOF ES^+): m/z calcd for $C_{22}H_{15}ClNO^+$ $[(M+H)^+]$, 344.0837; found, 344.0838.

(6-Chloroquinolin-3-yl)(pyridin-4-yl)methanone (3w)

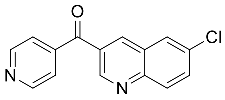

Brown solid; Yield = 86%, 115 mg; mp 146–148 °C; ^{1}H NMR (600 MHz, $CDCl_3$): δ = 7.67 (dd, J = 4.32 Hz, 2H, ArH), 7.85 (dd, J = 8.97 Hz, 1H, ArH), 7.95 (d, J = 2.34 Hz, 1H, ArH), 8.18 (d, J = 8.94 Hz, 1H, ArH), 8.49 (dd, J = 2.13 Hz, 1H, ArH), 8.92 (d, J = 5.94 Hz, 2H, ArH), 9.34 (d, J = 2.22 Hz, 1H, ArH); ^{13}C NMR (150 MHz, $CDCl_3$): δ = 122.7, 127.1, 127.8, 129.1, 131.2, 133.4, 133.9, 138.2, 143.3, 148.2, 150.0, 150.8, 150.8, 150.8, 193.4; HRMS (TOF ES^+): m/z calcd for $C_{15}H_{10}ClN_2O^+$ $[(M+H)^+]$, 269.0476; found, 269.0476.

(6-Chloroquinolin-3-yl)(thiophen-2-yl)methanone (3x)

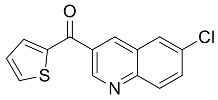

Brown solid; Yield = 89%, 121 mg; mp 153–155 °C; ^{1}H NMR (600 MHz, $CDCl_3$): δ = 7.26 (dd, J = 4.98 Hz, 1H, C=CH), 7.73 (dd, J = 3.78 Hz, 1H, ArH), 7.81 (dd, J = 8.94 Hz, 1H, C=CH), 7.85 (dd, J = 4.86 Hz, 1H, C=CH), 7.97 (d, J = 2.28 Hz, 1H, ArH), 8.17 (d, J = 8.94 Hz, 1H, ArH) , 8.59 (d, J = 2.22 Hz, 1H, ArH) , 9.35 (d, J = 2.22 Hz, 1H, ArH); ^{13}C NMR (150 MHz, $CDCl_3$): δ = 127.4,

127.5, 128.4, 131.2, 131.5, 132.6, 133.6, 135.2, 135.4, 136.6, 143.0, 147.8, 149.8, 185.8; HRMS (TOF ES$^+$): *m/z* calcd for $C_{14}H_9ClNOS^+$ [(M+H)$^+$], 274.0088; found, 274.0089.

(4-Methylquinolin-3-yl)(phenyl)methanone (3y)

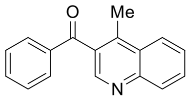

Brown solid; Yield = 71%, 88 mg; mp 91–93 °C; ^{1}H NMR (600 MHz, $CDCl_3$): δ = 2.70 (s, 3H, $ArCH_3$), 7.50–7.53 (m, 2H, ArH), 7.64–7.70 (m, 2H, ArH), 7.82–7.85 (m, 1H, ArH), 7.87 (dd, *J* = 6.84 Hz, 2H, ArH), 8.16 (d, *J* = 8.52 Hz, 1H, ArH), 8.19 (d, *J* = 8.40 Hz, 1H, ArH), 8.84 (s, 1H, ArH); ^{13}C NMR (150 MHz, $CDCl_3$): δ = 16.0, 124.5, 127.4, 127.6, 128.8, 128.8, 130.2, 130.2, 130.2, 130.5, 131.9, 133.9, 137.5, 143.7, 148.0, 148.6, 197.0; HRMS (TOF ES$^+$): *m/z* calcd for $C_{17}H_{14}NO^+$ [(M+H)$^+$], 248.1070; found, 248.1070.

(4-Fluorophenyl)(4-methylquinolin-3-yl)methanone (3z)

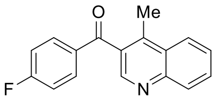

Yellow solid; Yield = 68%, 90 mg; mp 116–118 °C; ^{1}H NMR (600 MHz, $CDCl_3$): δ = 2.70 (s, 3H, $ArCH_3$), 7.18–7.21 (m, 2H, ArH), 7.69–7.71 (m, 1H, ArH), 7.83–7.86 (m, 1H, ArH), 7.89–7.92 (m, 2H, ArH), 8.16 (d, *J* = 8.10 Hz, 1H, ArH), 8.19 (d, *J* = 8.40 Hz, 1H, ArH), 8.82 (s, 1H, ArH); ^{13}C NMR (150 MHz, $CDCl_3$): δ = 15.9, 116.1 (*J* = 22.5 Hz), 116.1 (*J* = 22.5 Hz), 124.4, 127.4, 127.6, 130.3, 130.5, 131.6, 132.9 (*J* = 9.0 Hz), 132.9 (*J* = 9.0 Hz), 134.0 (*J* = 3. 0 Hz), 143.6, 148.1, 148.4, 166.2 (*J* = 255.1 Hz), 195.4; HRMS (TOF ES$^+$): *m/z* calcd for $C_{17}H_{13}FNO^+$ [(M+H)$^+$], 266.0976; found, 266.0977.

(2-Chlorophenyl)(4-phenylquinolin-3-yl)methanone (3a')

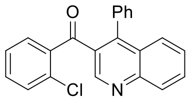

White solid; Yield = 83%, 142 mg; mp 110–112 °C; ^{1}H NMR (600 MHz, $CDCl_3$): δ = 7.06–7.08 (m, 1H, ArH), 7.14–7.22 (m, 5H, ArH), 7.26–7.27 (m, 3H, ArH), 7.53–7.55 (m, 1H, ArH), 7.68 (d, *J* = 8.55 Hz, 1H, ArH), 7.82–7.85 (m, 1H, ArH), 8.25 (dd, *J* = 8.46 Hz, 1H, ArH), 9.17 (s, 1H, ArH); ^{13}C NMR (150 MHz, $CDCl_3$): δ = 126.4, 126.4, 127.1, 127.5, 128.1, 128.1, 128.5, 129.6, 129.6, 129.7, 130.1, 130.7, 131.0, 131.6, 131.9, 132.0, 134.8, 138.2, 147.8, 149.1, 149.7, 196.3; HRMS (TOF ES$^+$): *m/z* calcd for $C_{22}H_{15}ClNO^+$ [(M+H)$^+$], 344.0837; found, 344.0838.

[1,1'-Biphenyl]-4-yl(4-phenylquinolin-3-yl)methanone (3b')

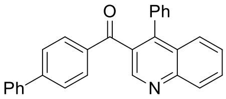

Yellow solid; Yield = 88%, 170 mg; mp 171–173 °C; ^{1}H NMR (600 MHz, $CDCl_3$): δ = 7.31–7.35 (m, 5H, ArH), 7.40–7.43 (m, 1H, ArH), 7.46–7.48 (m, 2H, ArH), 7.55 (d, *J* = 8.46 Hz, 2H, ArH), 7.58–7.61 (m, 3H, ArH), 7.73 (d, *J* = 8.40 Hz, 2H, ArH), 7.82–7.86 (m, 2H, ArH), 8.28 (d, *J* = 8.34

Hz, 1H, ArH), 9.05 (s, 1H, ArH); ^{13}C NMR (150 MHz, $CDCl_3$): δ = 126.5, 126.8, 126.9, 126.9, 127.3, 127.3, 127.5, 128.3, 128.3, 128.3, 128.6, 129.0, 129.0, 129.8, 130.1, 130.1, 130.4, 130.4, 130.5, 131.9, 135.0, 136.0, 139.7, 145.9, 147.1, 148.5, 148.8, 196.3; HRMS (TOF ES^+): *m/z* calcd for $C_{28}H_{20}NO^+$ $[(M+H)^+]$, 386.1539; found, 386.1539.

Furan-2-yl(4-phenylquinolin-3-yl)methanone (3c')

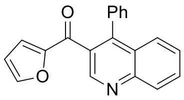

Yellow solid; Yield = 91%, 136 mg; mp 113–115 °C; ^1H NMR (600 MHz, $CDCl_3$): δ = 6.42 (d, *J* = 3.60 Hz, 1H, C=CH), 6.92 (d, *J* = 3.48 Hz, 1H, ArH), 7.36–7.40 (m, 5H, ArH), 7.50 (s, 1H, C=CH), 7.56–7.58 (m, 1H, ArH), 7.81–7.84 (m, 2H, ArH), 8.25 (d, *J* = 8.94 Hz, 1H, C=CH), 9.06 (s, 1H, ArH); ^{13}C NMR (150 MHz, $CDCl_3$): δ = 112.5, 120.7, 126.5, 126.9, 127.5, 128.3, 128.3, 128.6, 129.8, 130.0, 130.0, 130.7, 130.9, 134.8, 147.4, 147.4, 148.3, 148.9, 152.6, 183.3; HRMS (TOF ES^+): *m/z* calcd for $C_{20}H_{14}NO_2^+$ $[(M+H)^+]$, 300.1019; found, 300.1019.

3-((2-(Hydroxymethyl)phenyl)amino)-1-phenylprop-2-en-1-one (4)

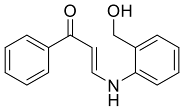

Yellow solid; Yield = 68%, 86 mg; mp 123–125 °C; ^1H NMR (600 MHz, $CDCl_3$): δ = 4.88 (d, *J* = 4.62 Hz, 2H, CCH_2OH), 6.09 (d, *J* = 7.80 Hz, 1H, ArH), 7.07–7.10 (m, 1H, ArH), 7.26 (d, *J* = 8.04 Hz, 1H, ArH), 7.31 (d, *J* = 7.50 Hz, 1H, ArH), 7.35–7.38 (m, 1H, ArH), 7.45–7.47 (m, 2H, ArH), 7.50–7.57 (m, 2H, ArH), 7.97 (d, *J* = 7.08 Hz, 2H, ArH), 12.54 (d, *J* = 12.30 Hz, 1H, NH); ^{13}C NMR (150 MHz, $CDCl_3$): δ = 63.2, 94.5, 115.1, 123.5, 127.4, 127.4, 128.4, 128.4, 128.9, 129.4, 129.7, 131.6, 139.2, 139.8, 145.2, 190.9; HRMS (TOF ES^+): *m/z* calcd for $C_{16}H_{16}NO_2^+$ $[(M+H)^+]$, 254.1176; found, 254.1176.

5. X-ray Structure and Data[1] of 3j (CCDC 1846910).

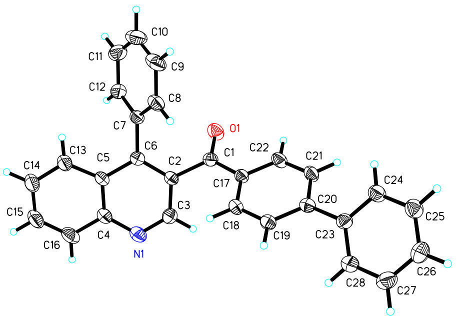

Figure S1 X-Ray crystal structure of 3j.

Table S1 Crystal data and structure refinement for **3j.**

Empirical formula	$C_{28}H_{19}NO_2$
Formula weight	385.44
Temperature	150.15 K
Wavelength	0.71073 Å
Crystal system, space group	Monoclinic, P 21/c
Unit cell dimensions	a = 10.305(4) A alpha = 90 deg.
	b = 20.188(8) A beta = 94.629(8) deg.
	c = 9.940(4) A gamma = 90 deg.
Volume	2061.1(14) A^3
Z, Calculated density	4, 1.242 Mg/m^3
Absorption coefficient	0.075 mm^-1
F(000)	808.0
Theta range for data collection	2.225 to 27.655 deg.
Limiting indices	-12<=h<=12, -24<=k<=23, -11<=l<=7
Completeness to theta = 25.026	99.2%
Absorption correction	Multi-scan
Refinement method	ShelXL (Sheldrick, 2015)
Data/restraints/parameters	3607 / 0 / 271
Goodness-of-fit on F^2	0.965
Final R indices [I>2sigma(I)]	R1 = 0.1471, wR2 = 0.1299
R indices (all data)	R1 = 0.0643, wR2 = 0.1580
Largest diff. peak and hole	0.234 and -0.279 e.A^-3

6. ^{1}H NMR and ^{13}C NMR spectra for spectroscopic data.

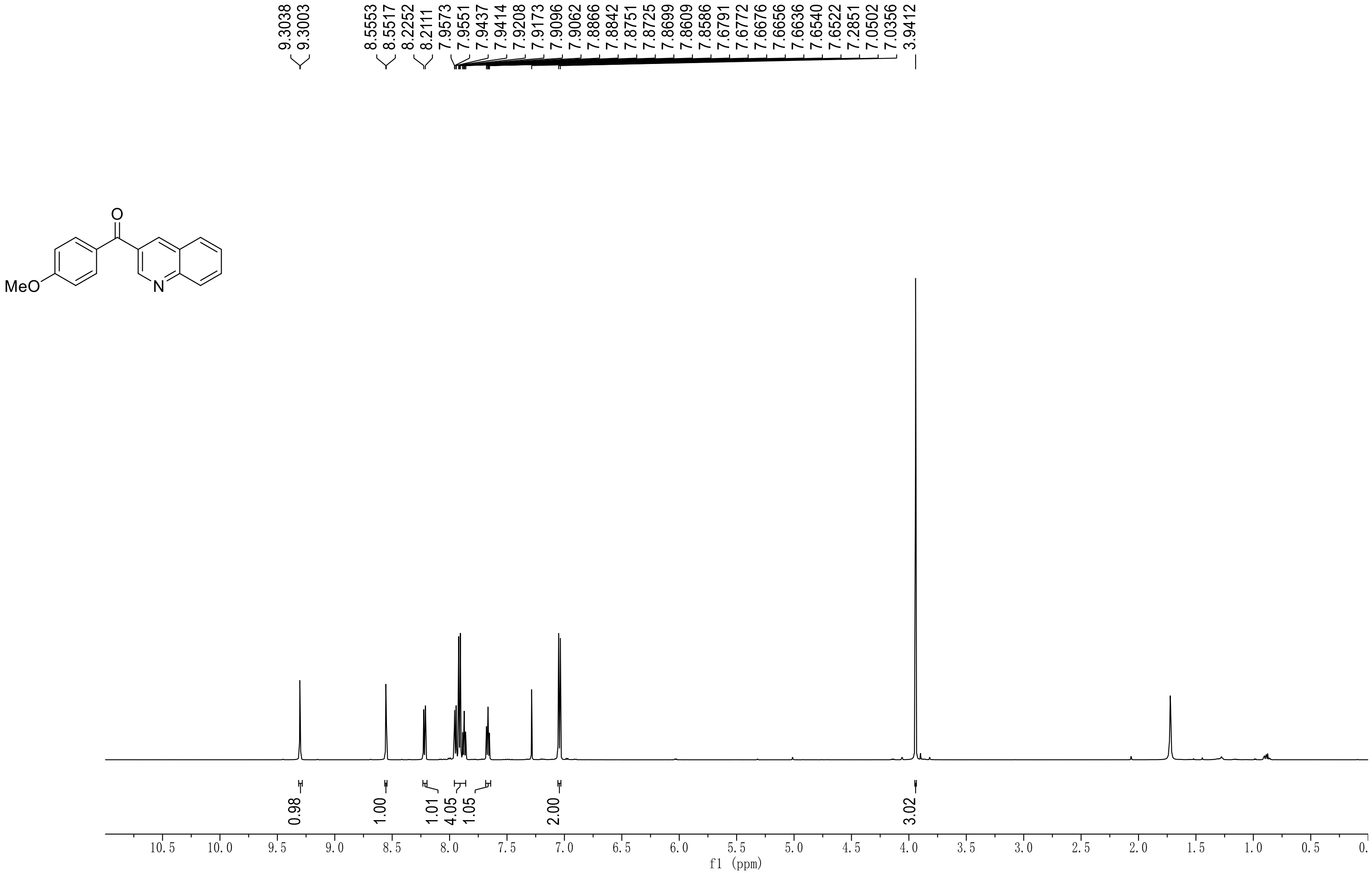

Figure 2. ^{1}H NMR (600 MHz, $CDCl_3$) spectra of compound **3a**

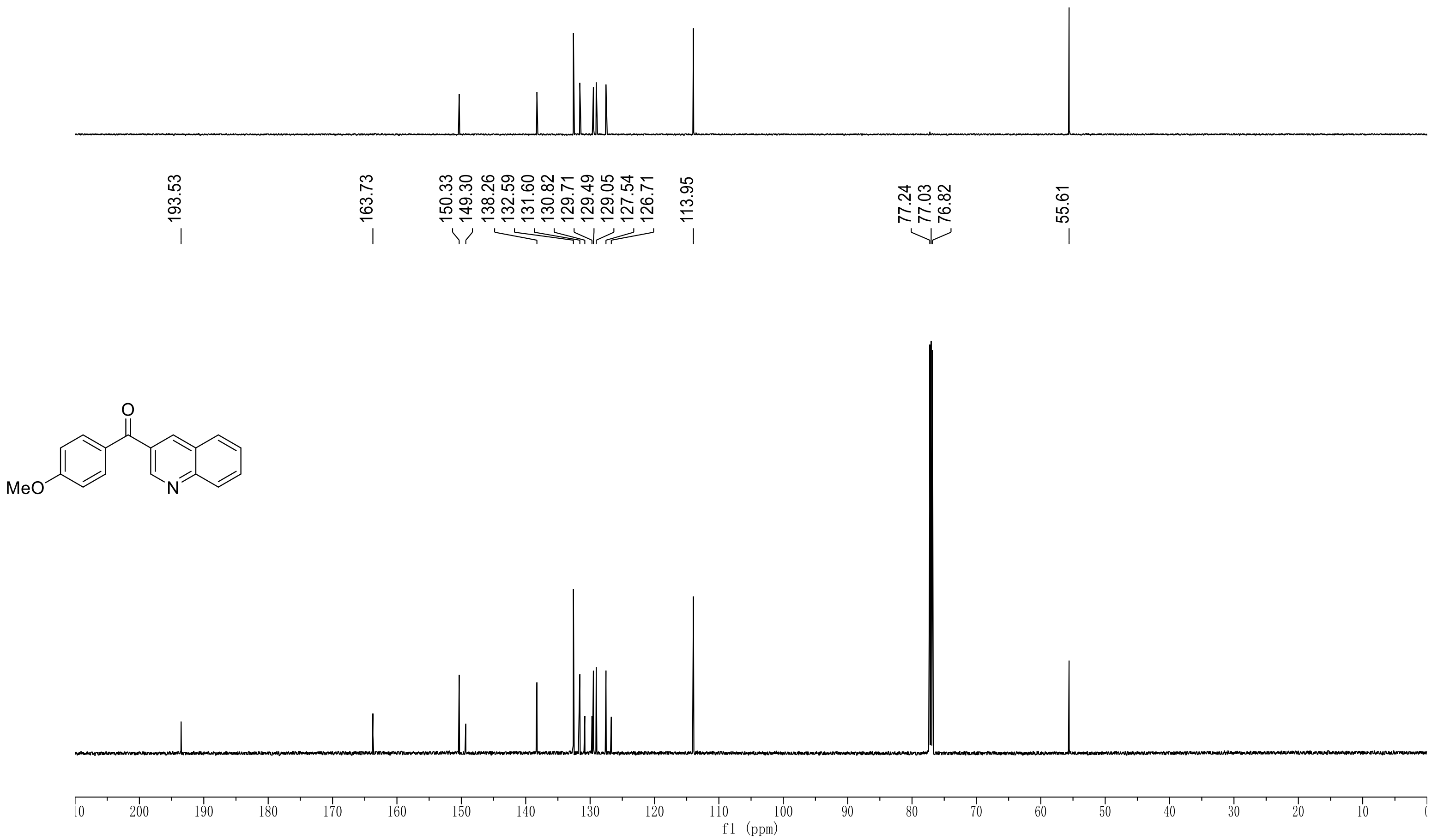

Figure 3. ^{13}C NMR (150 MHz, $CDCl_3$) spectra of compound **3a**

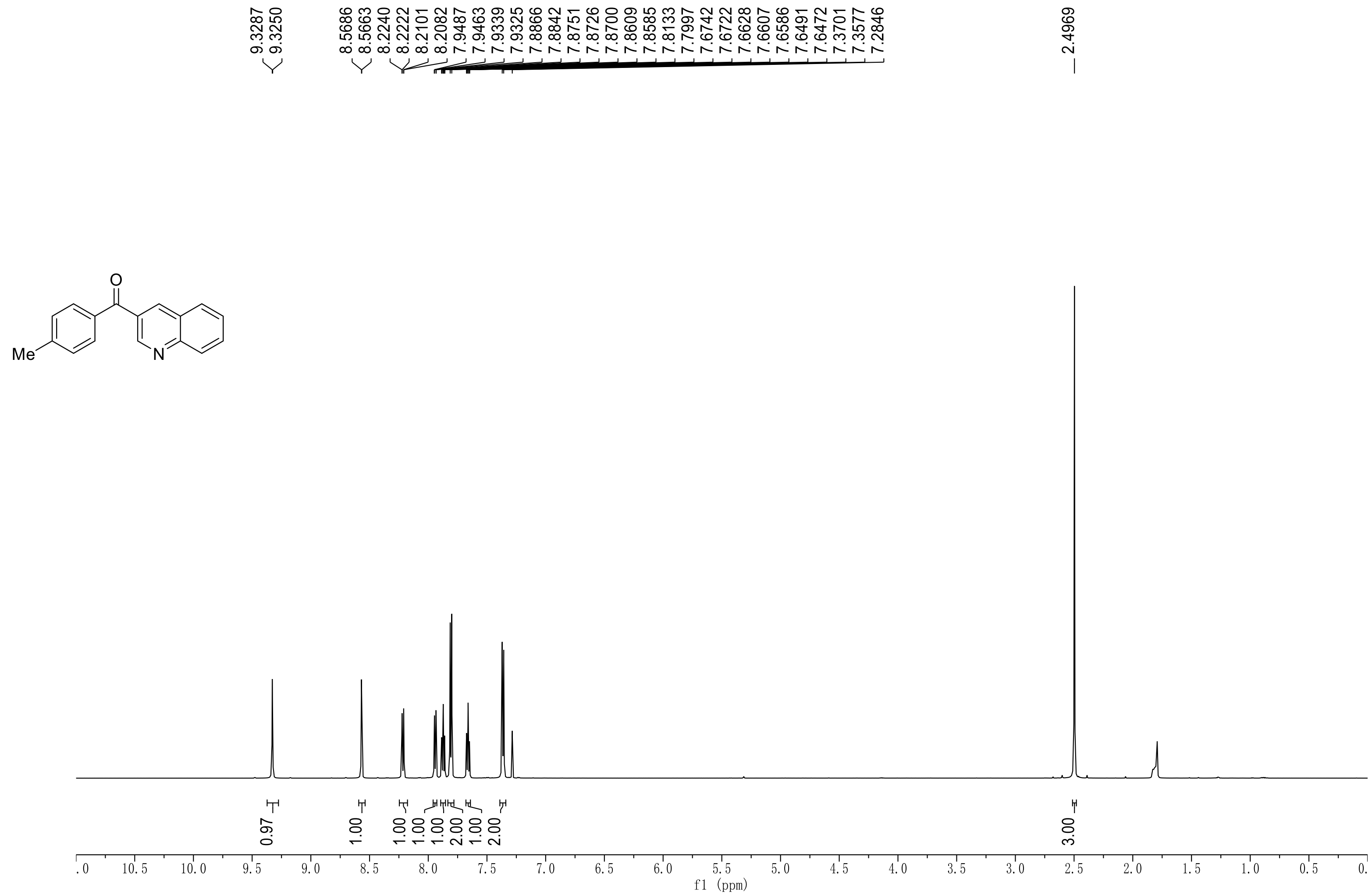

Figure 4. ^{1}H NMR (600 MHz, $CDCl_3$) spectra of compound **3b**

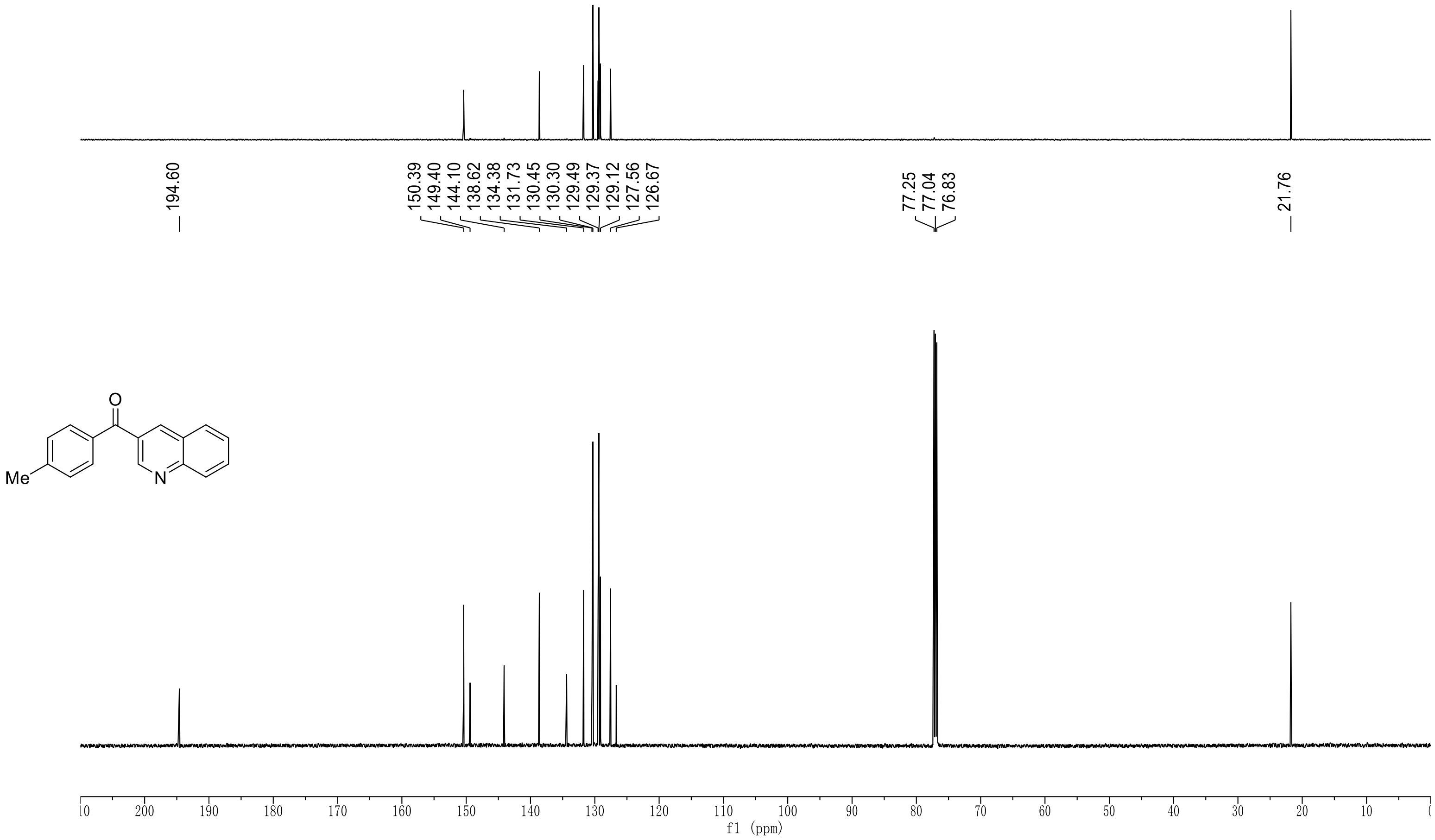

Figure 5. ^{13}C NMR (150 MHz, $CDCl_3$) spectra of compound **3b**

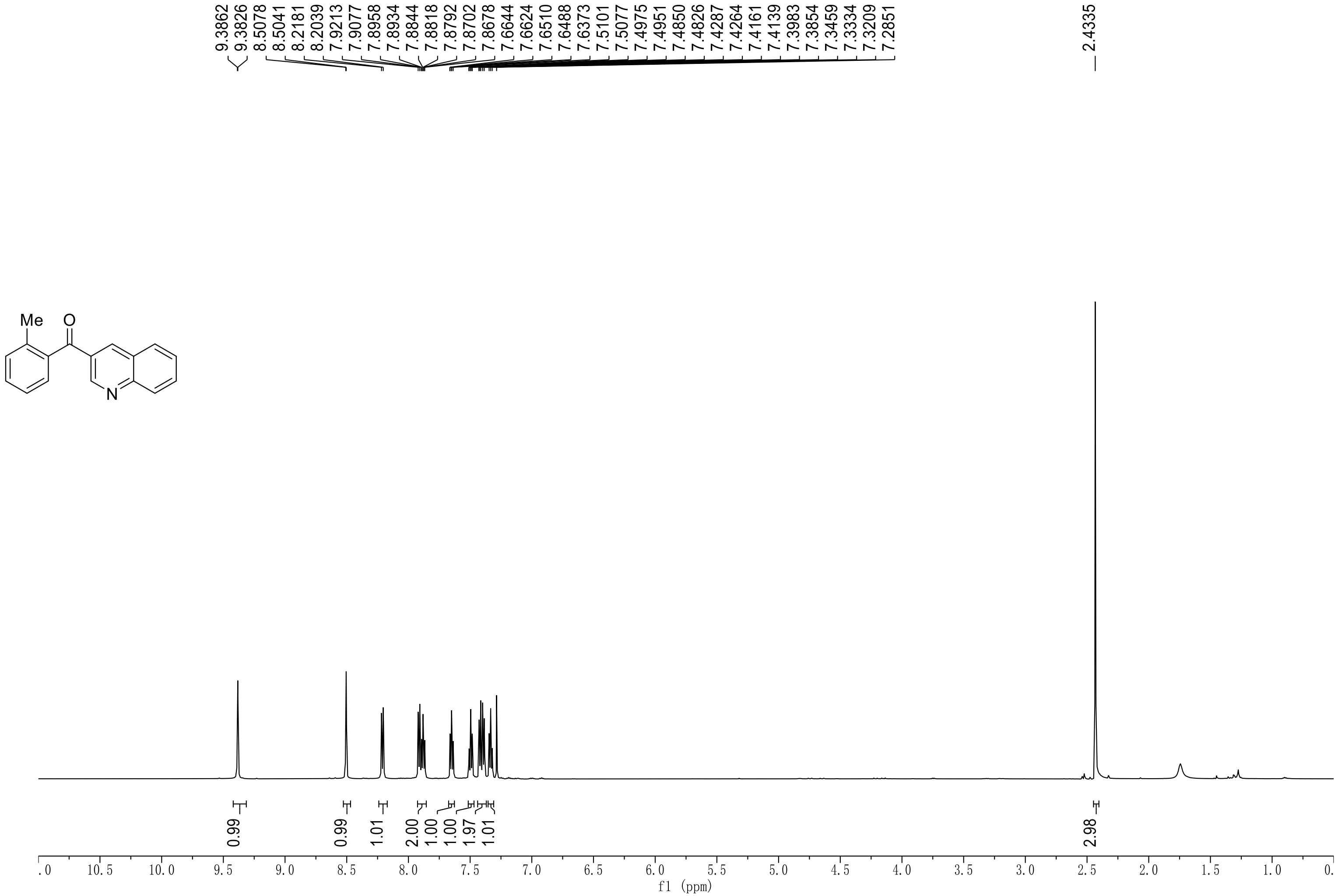

Figure 6. ^{1}H NMR (600 MHz, $CDCl_3$) spectra of compound **3c**

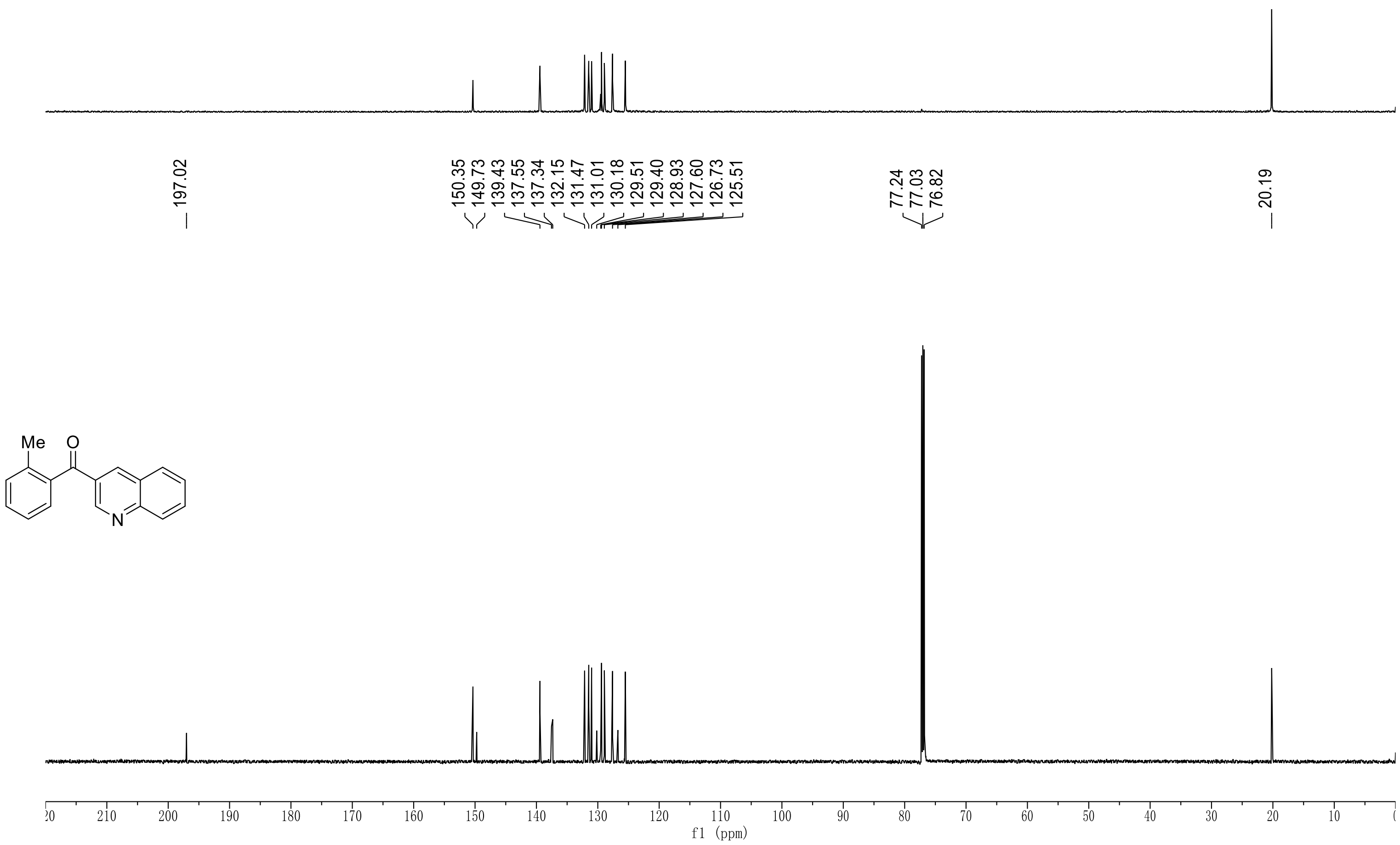

Figure 7. ^{13}C NMR (150 MHz, $CDCl_3$) spectra of compound **3c**

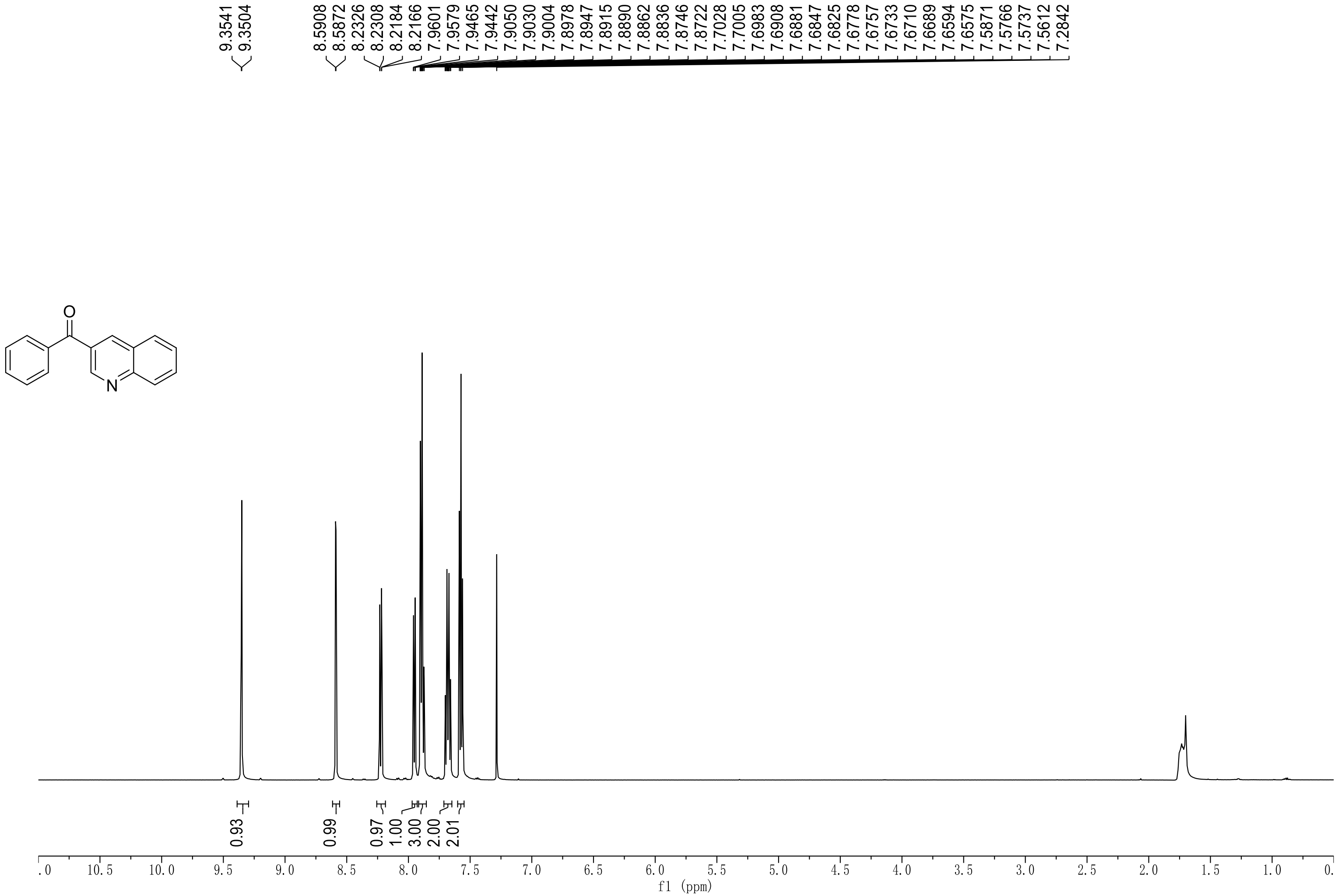

Figure 8. ^{1}H NMR (600 MHz, $CDCl_3$) spectra of compound **3d**

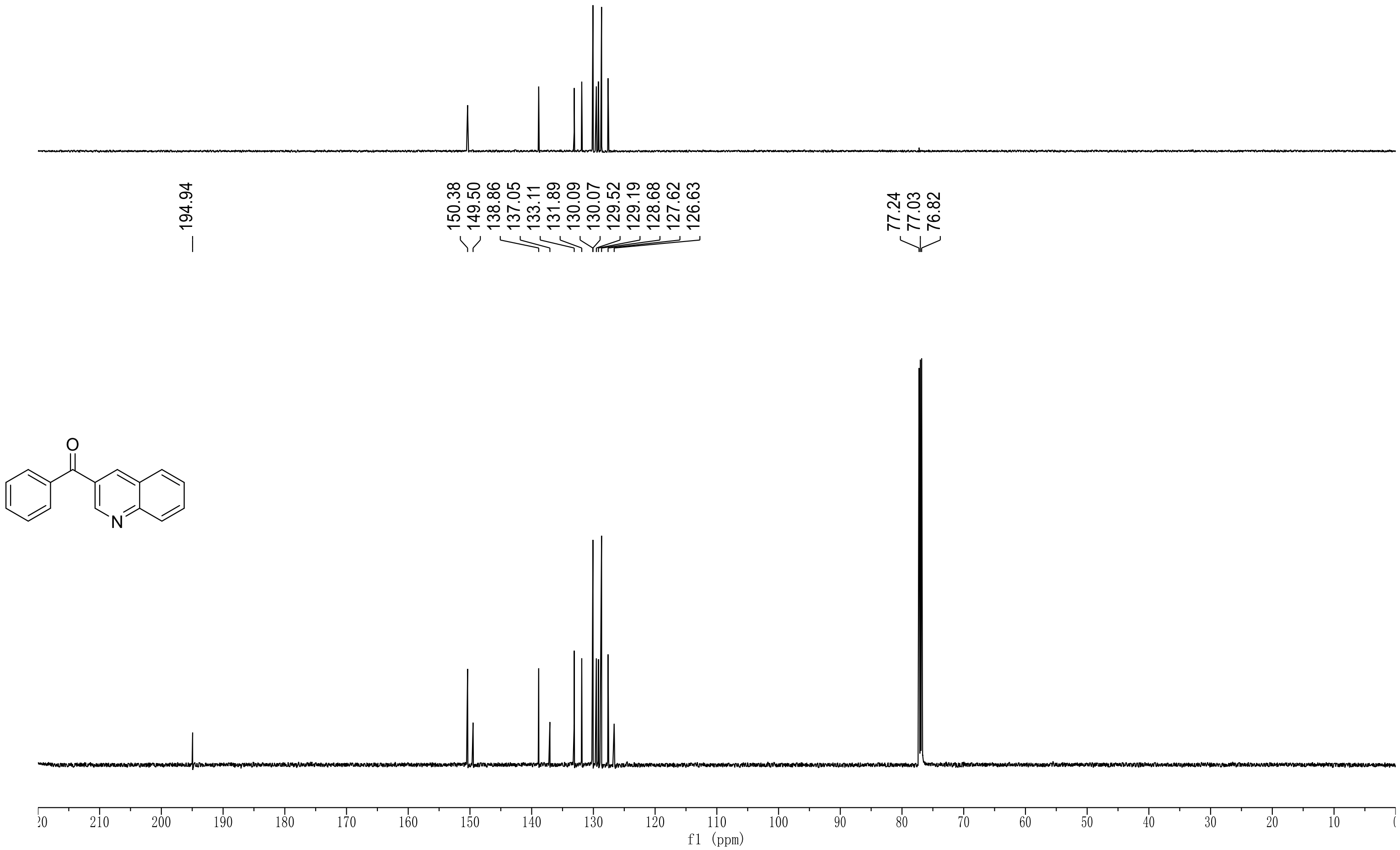

Figure 9. ^{13}C NMR (150 MHz, $CDCl_3$) spectra of compound **3d**

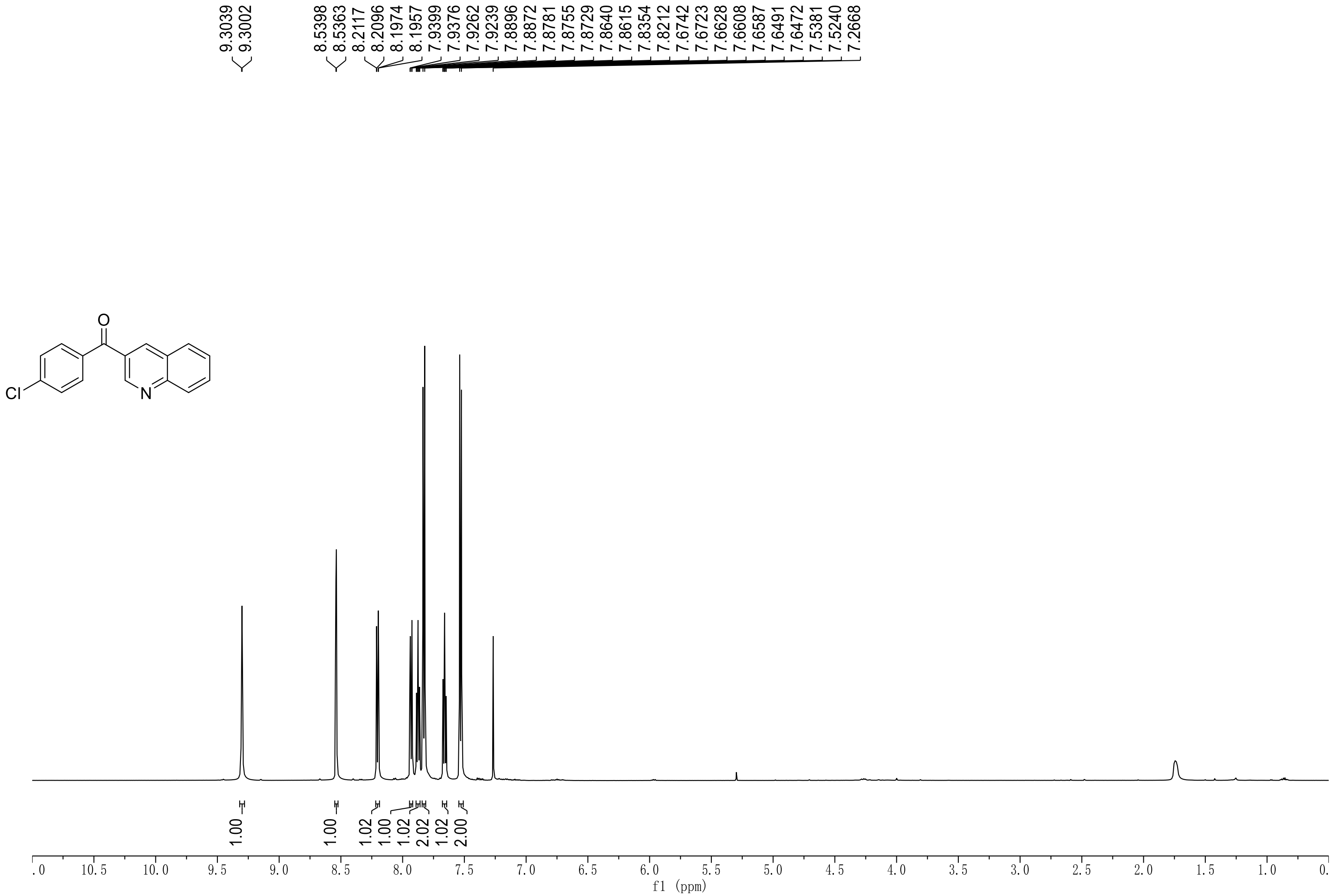

Figure 10. ^{1}H NMR (600 MHz, $CDCl_3$) spectra of compound **3e**

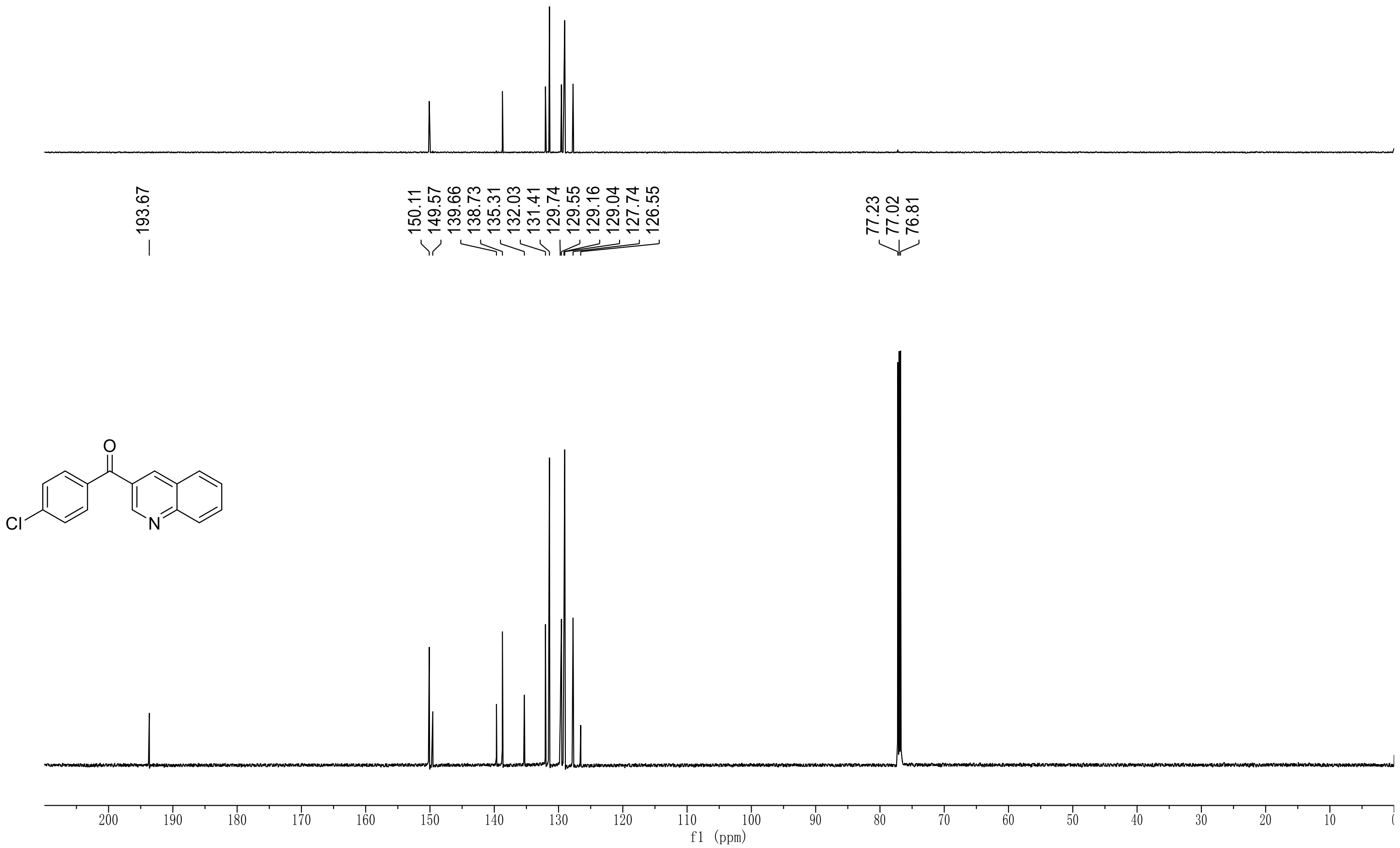

Figure 11. ^{13}C NMR (150 MHz, $CDCl_3$) spectra of compound **3e**

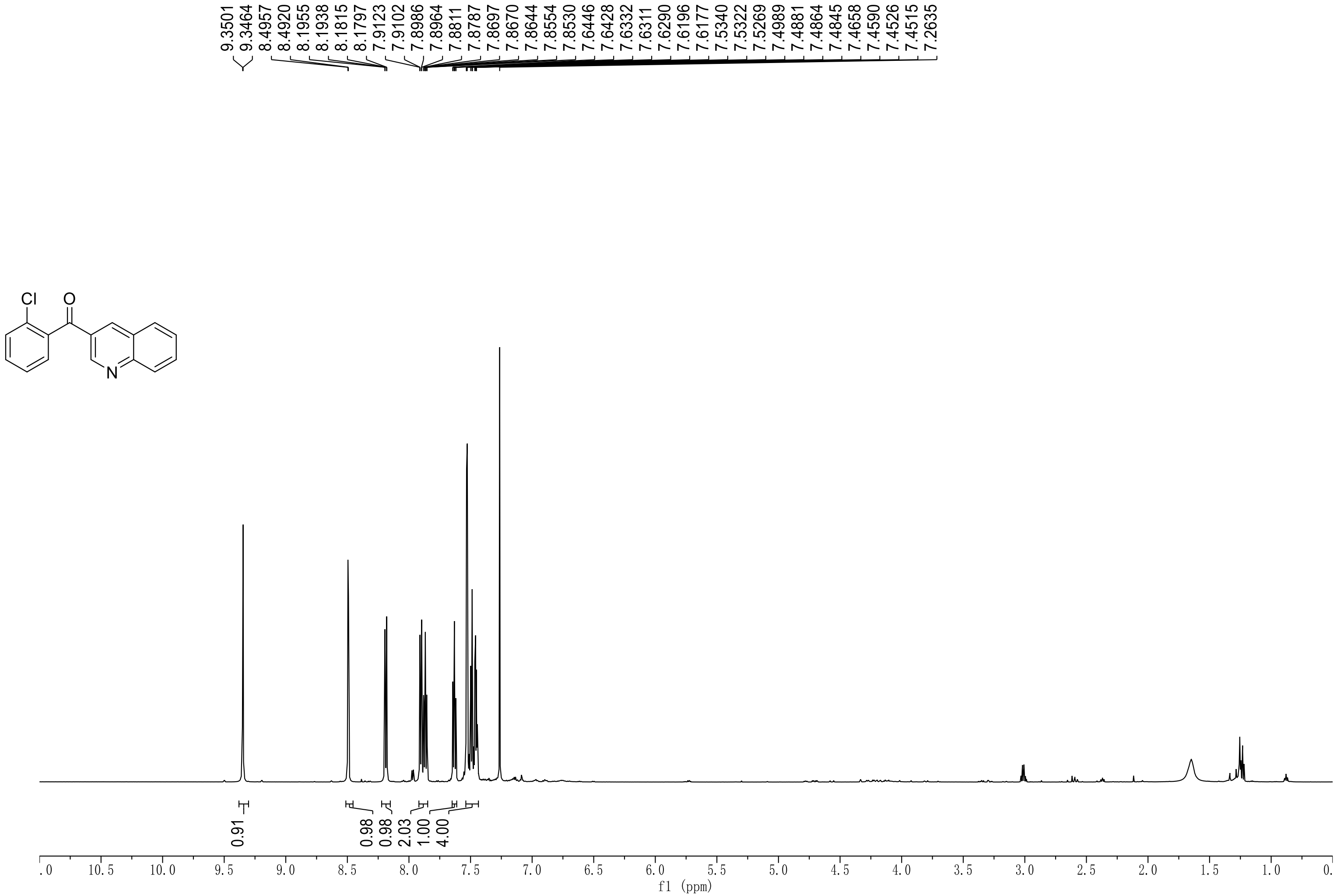

Figure 12. ¹H NMR (600 MHz, $CDCl_3$) spectra of compound **3f**

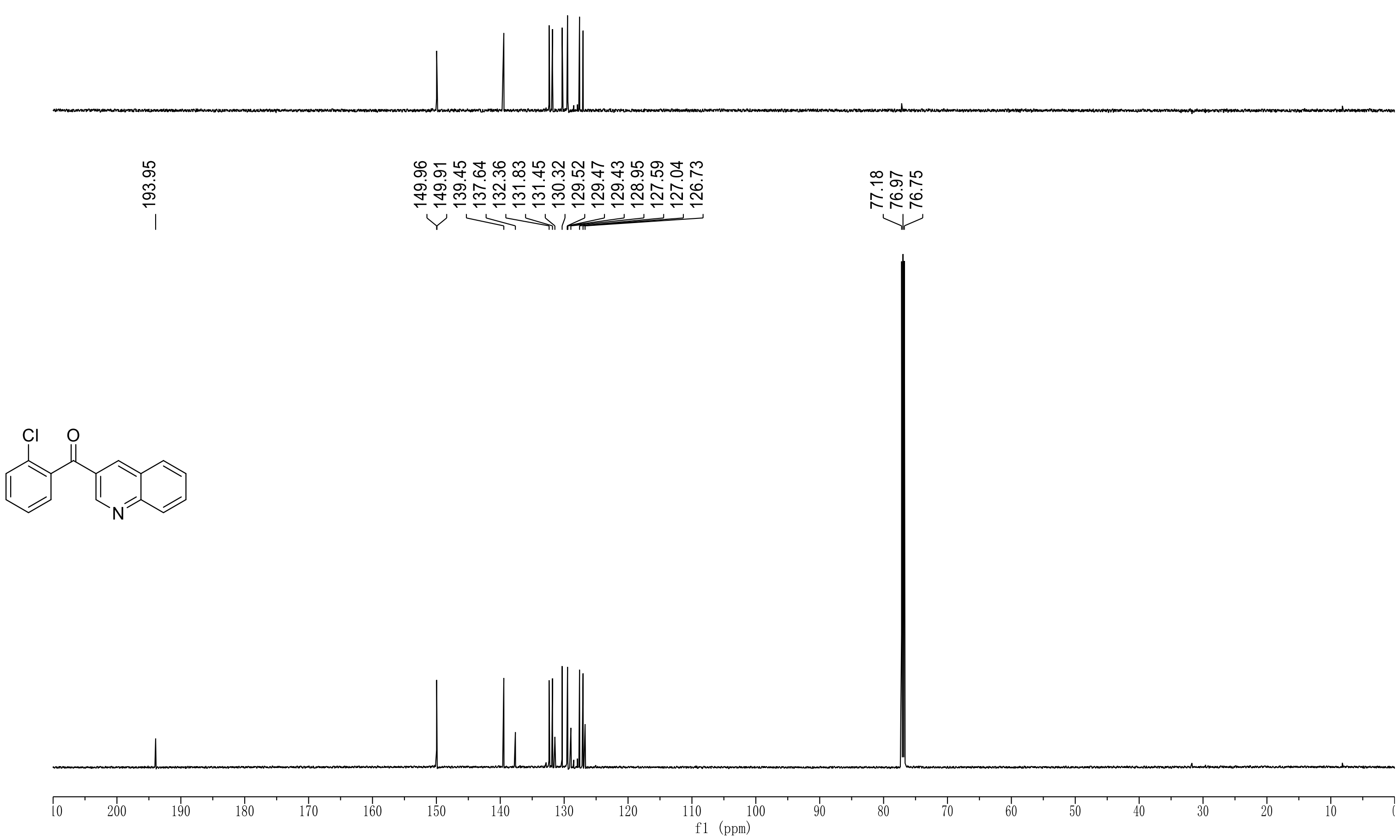

Figure 13. ^{13}C NMR (150 MHz, $CDCl_3$) spectra of compound **3f**

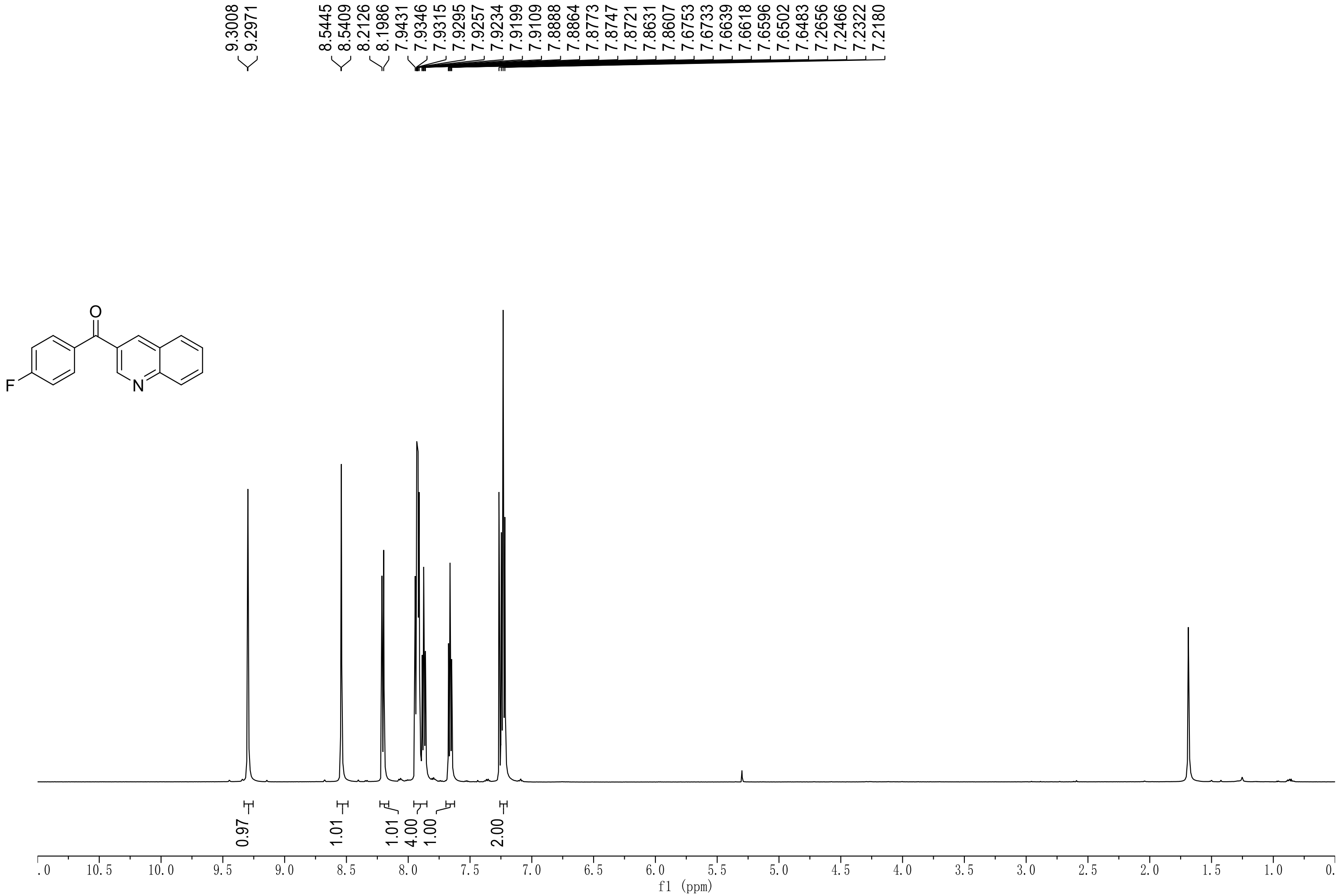

Figure 14. ^{1}H NMR (600 MHz, $CDCl_3$) spectra of compound **3g**

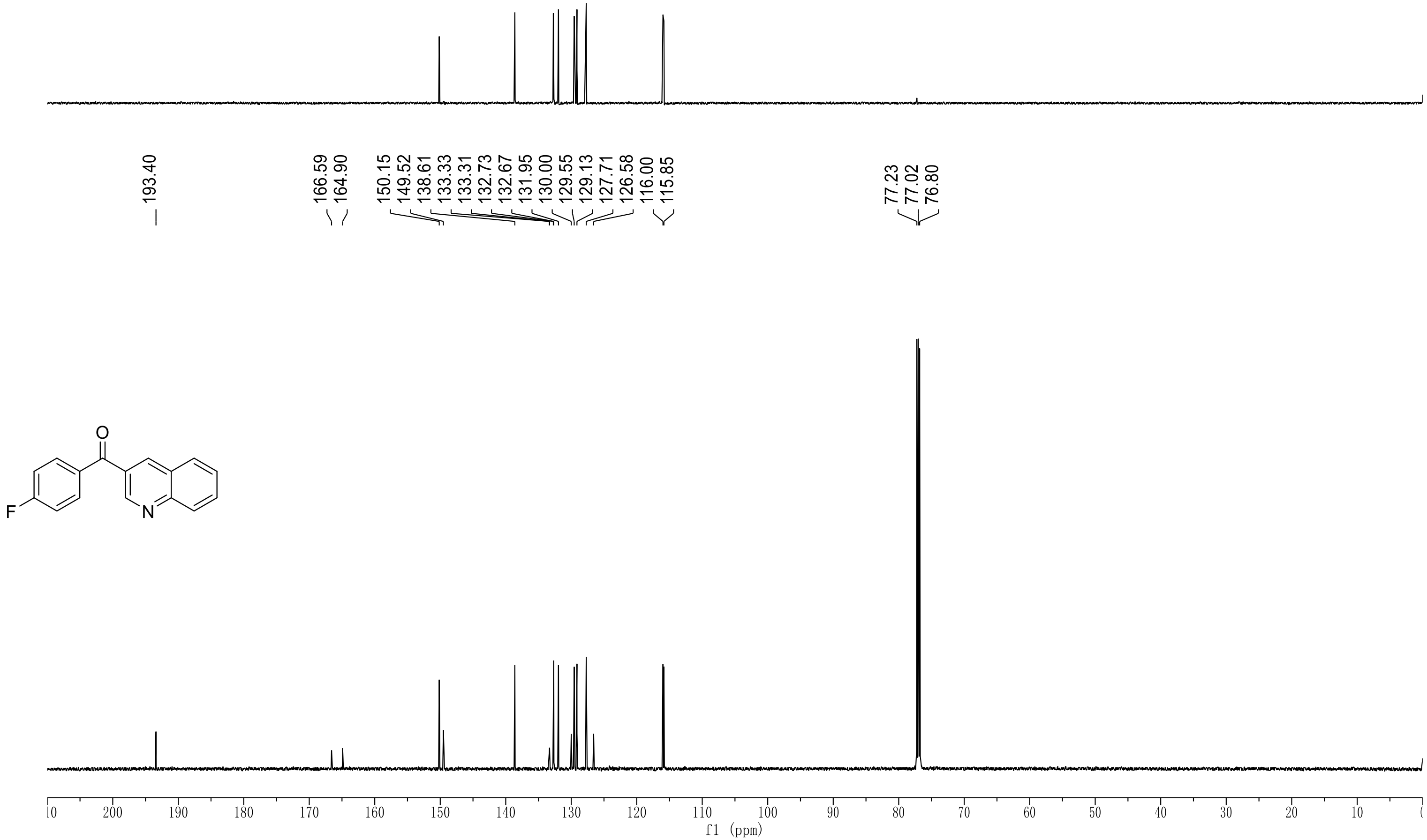

Figure 15. ^{13}C NMR (150 MHz, $CDCl_3$) spectra of compound **3g**

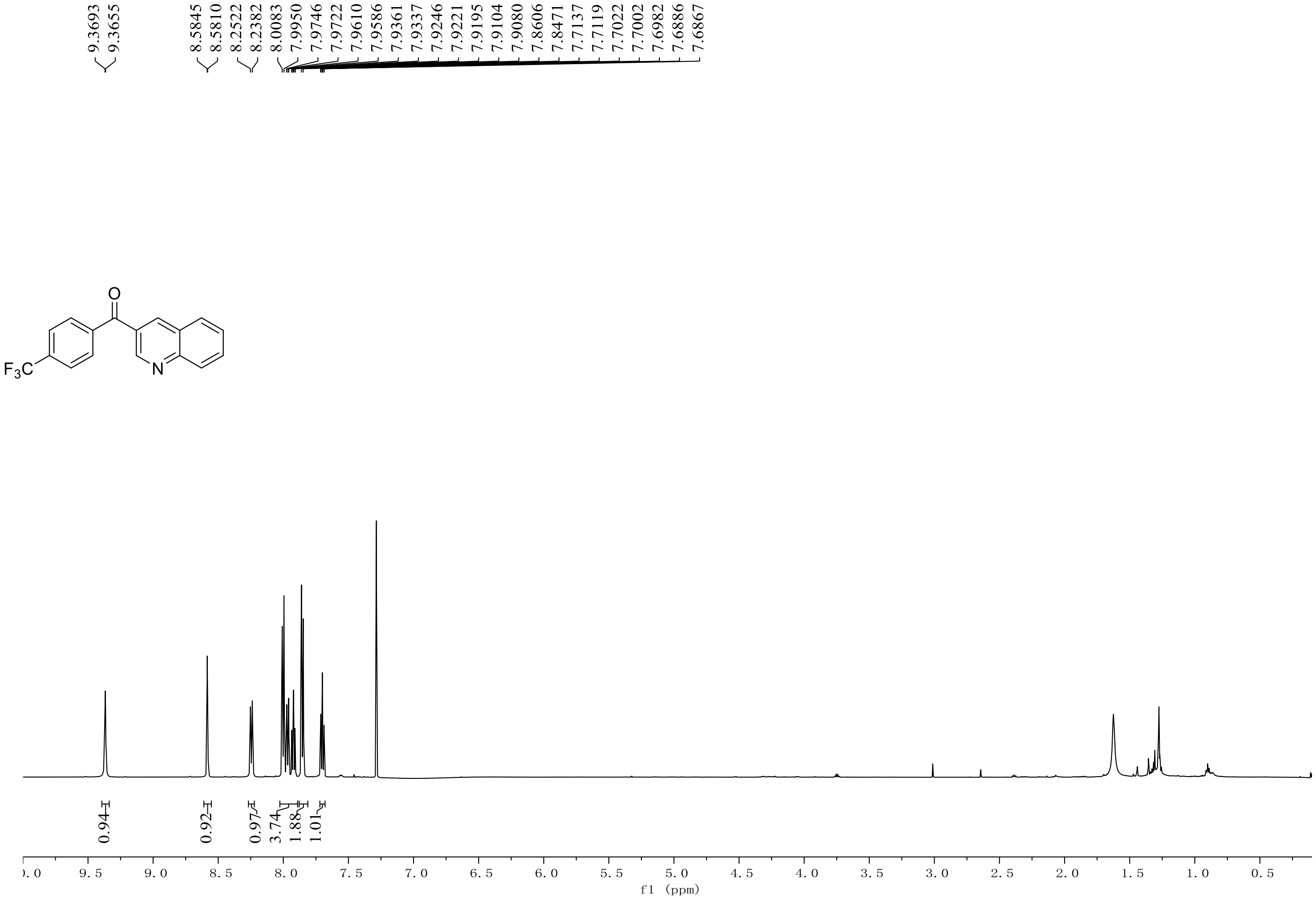

Figure 16. ^{1}H NMR (600 MHz, $CDCl_3$) spectra of compound **3h**

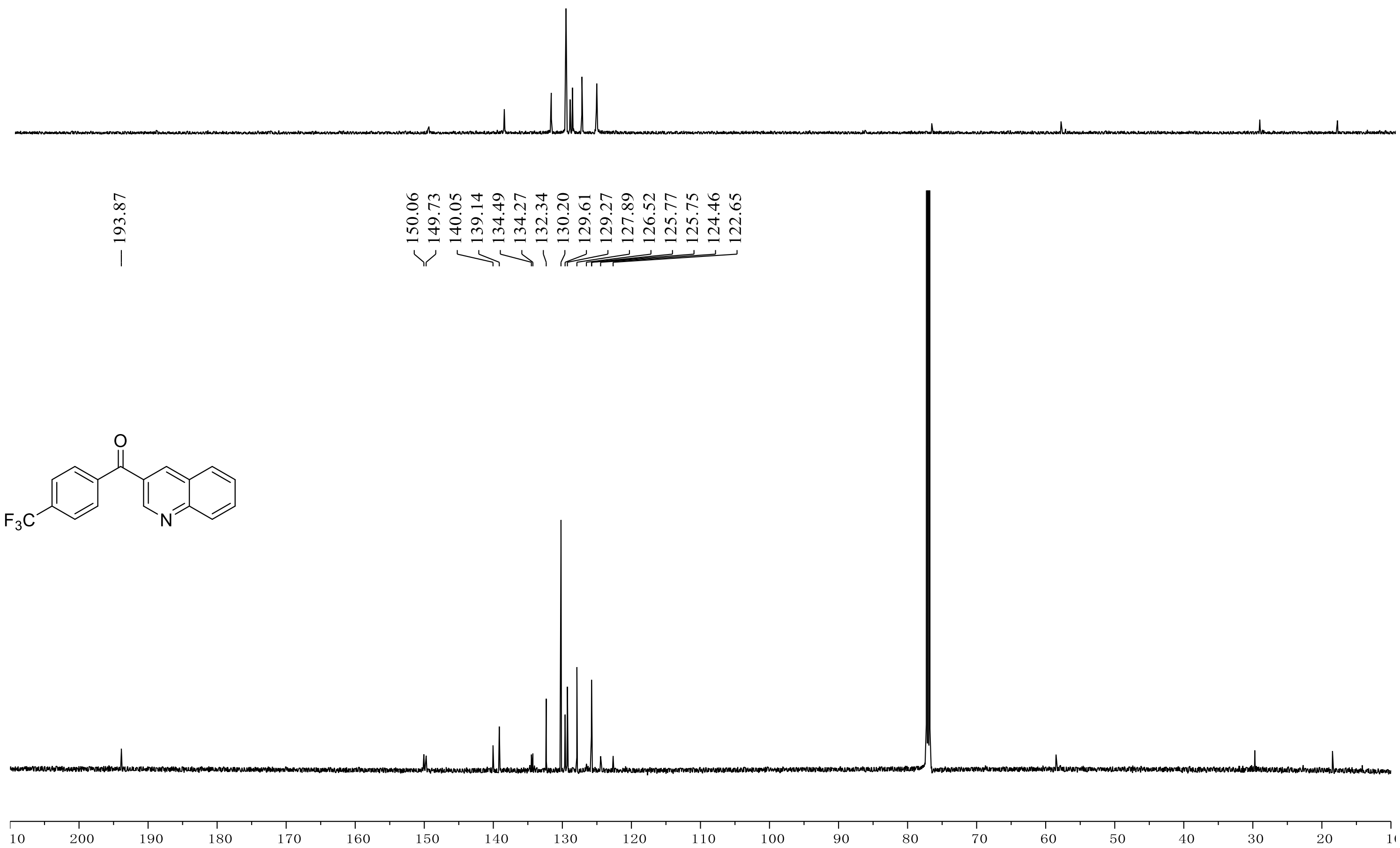

Figure 17. ^{13}C NMR (150 MHz, $CDCl_3$) spectra of compound **3h**

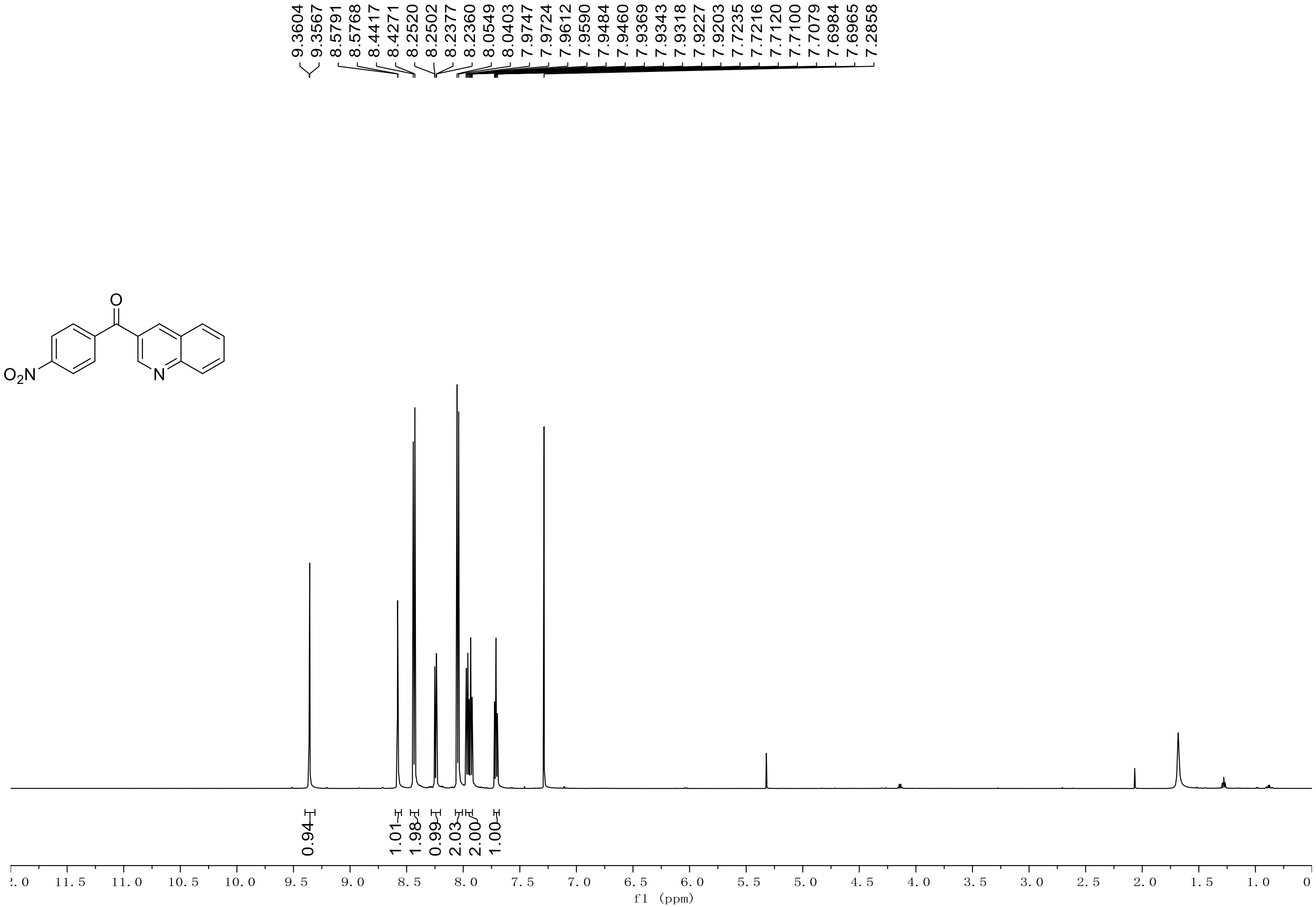

Figure 18. ^{1}H NMR (600 MHz, $CDCl_3$) spectra of compound **3i**

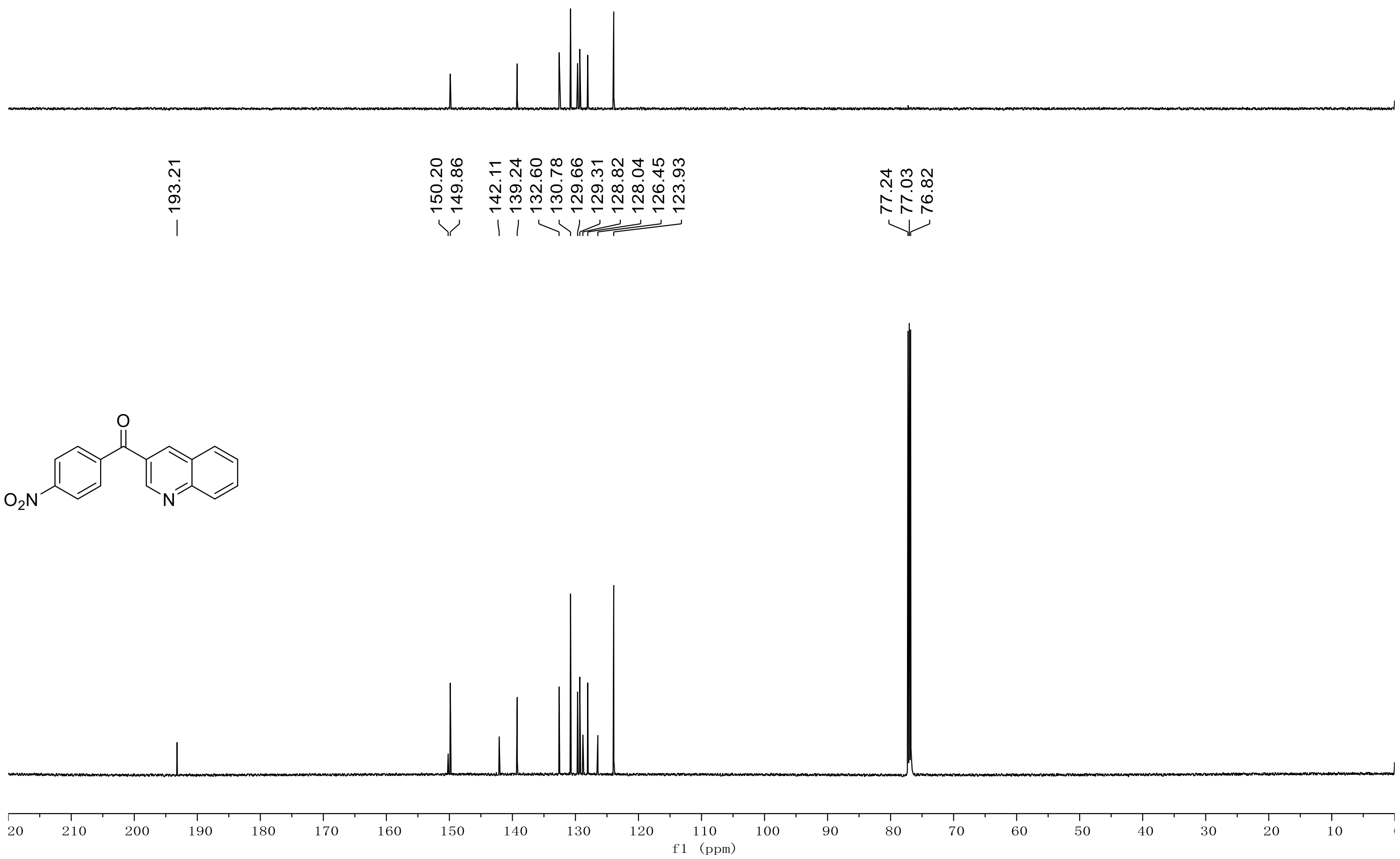

Figure 19. ^{13}C NMR (150 MHz, $CDCl_3$) spectra of compound **3i**

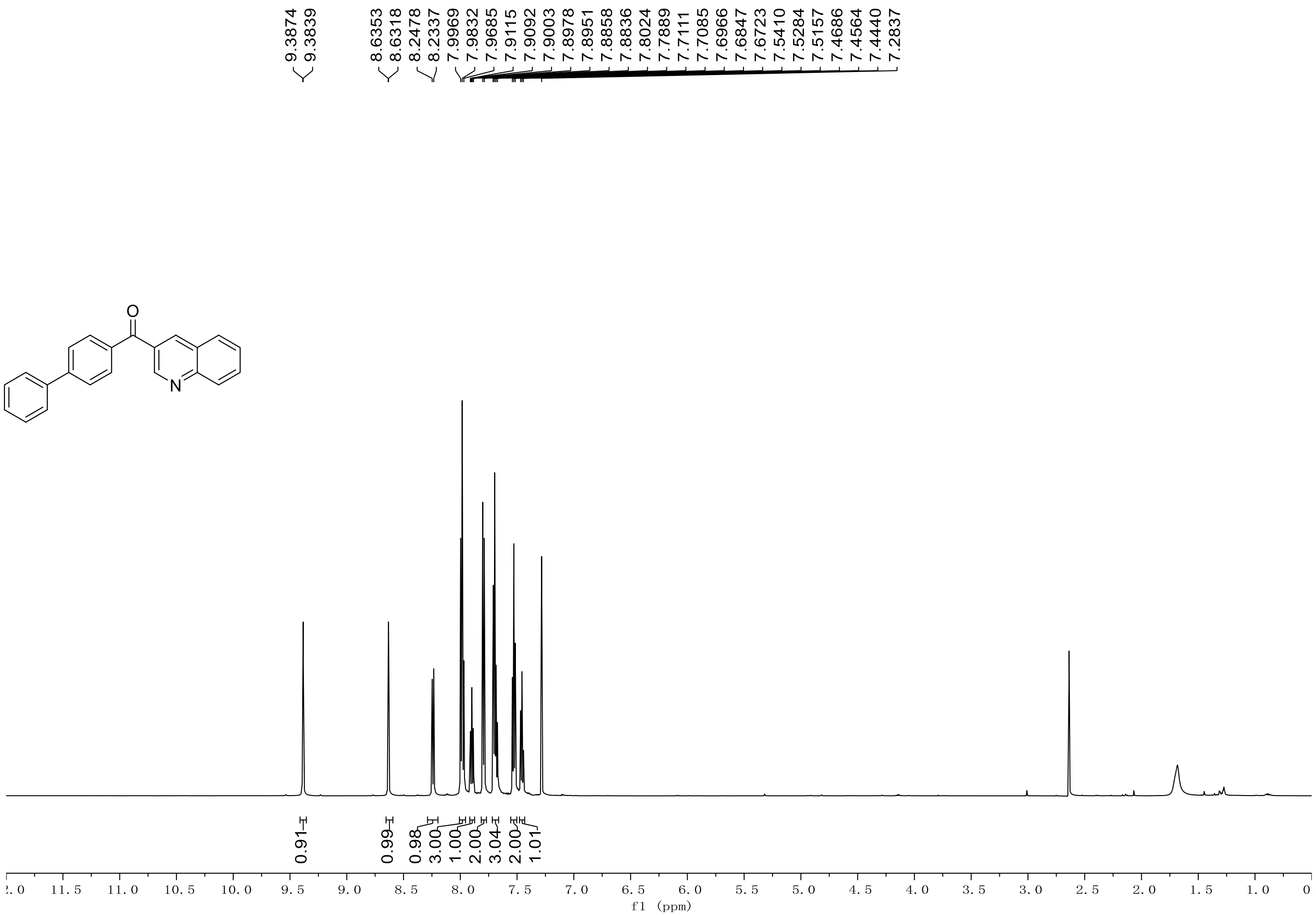

Figure 20. ^{1}H NMR (600 MHz, $CDCl_3$) spectra of compound **3j**

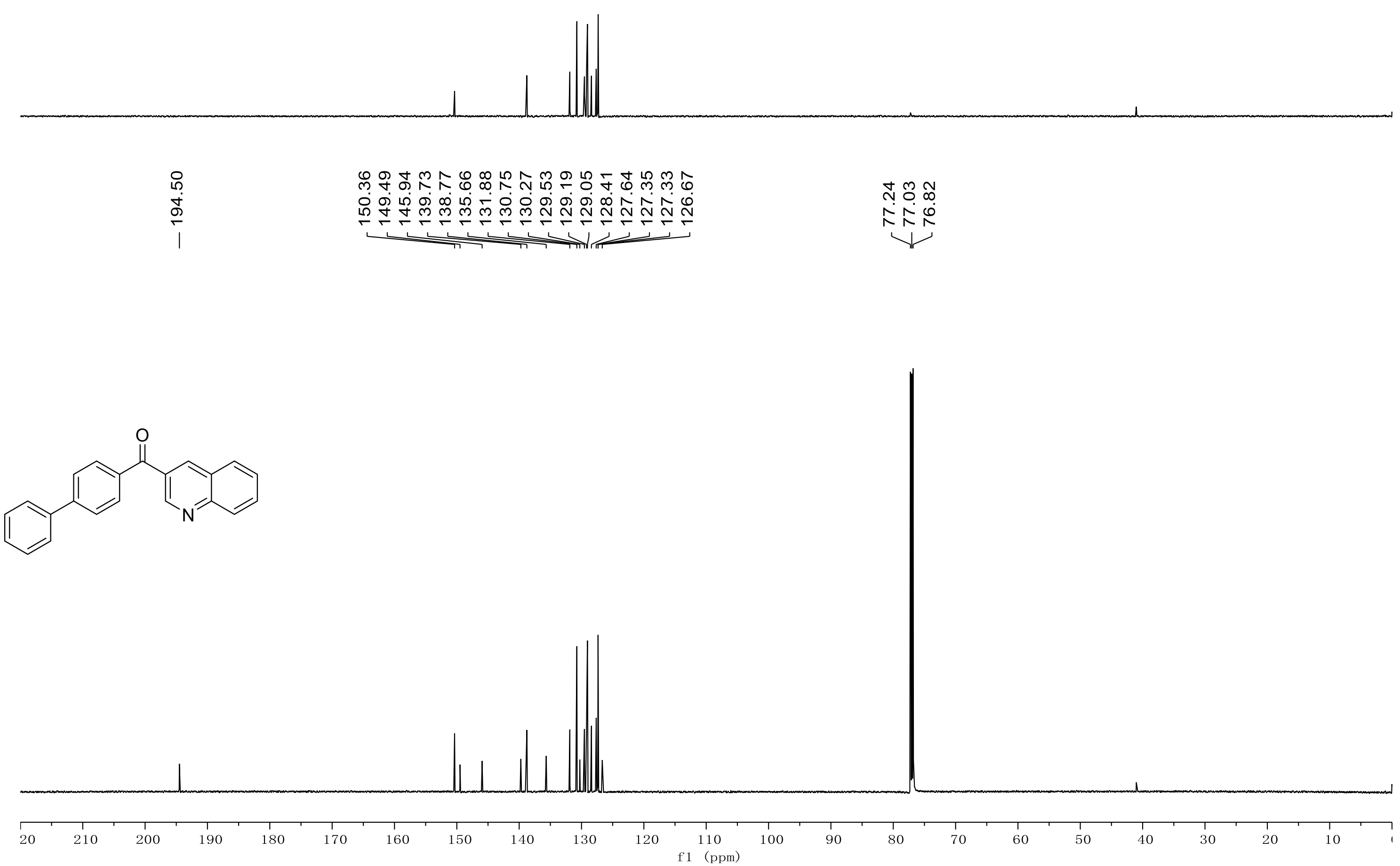

Figure 21. ^{13}C NMR (150 MHz, $CDCl_3$) spectra of compound **3j**

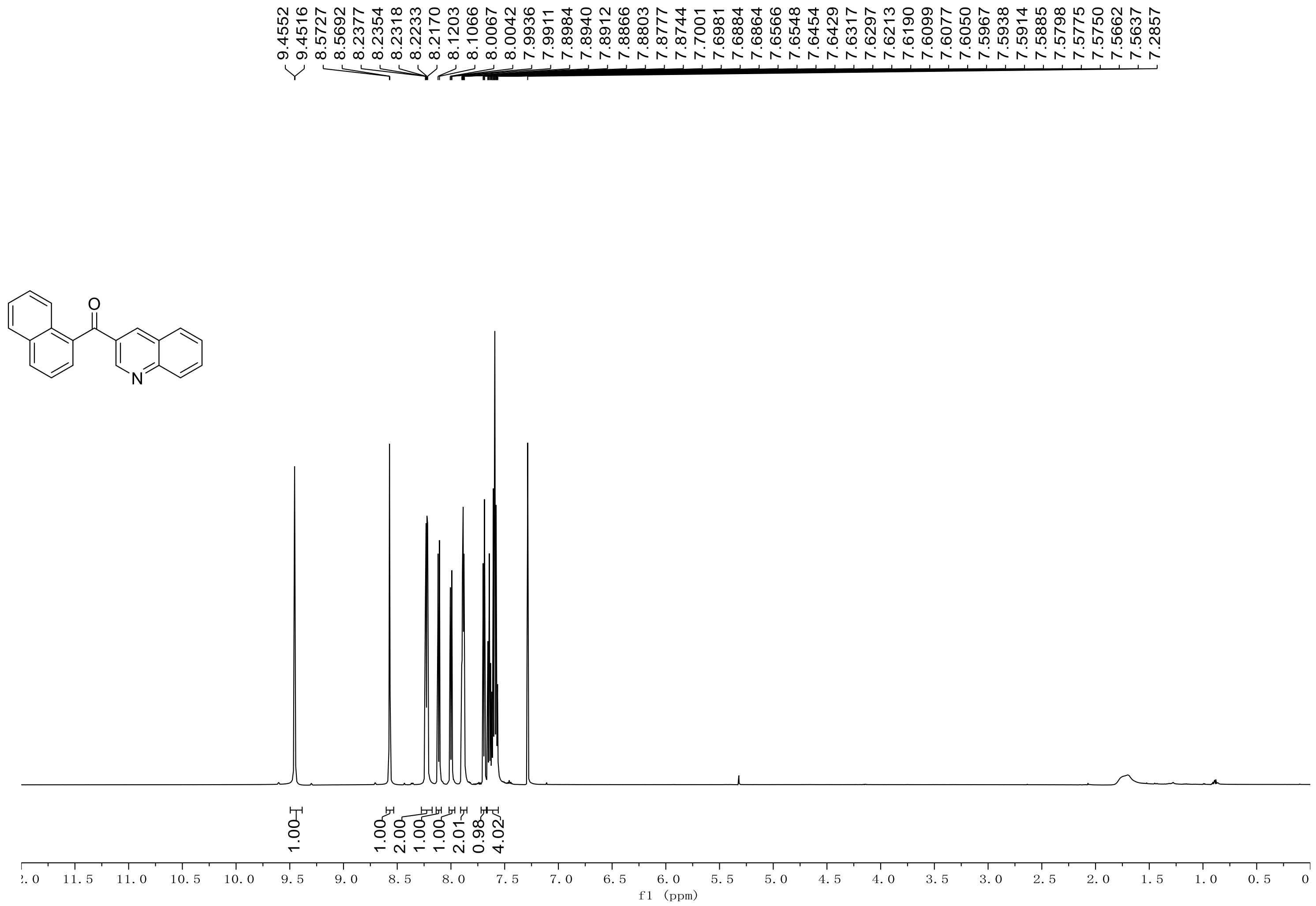

Figure 22. ^{1}H NMR (600 MHz, $CDCl_3$) spectra of compound **3k**

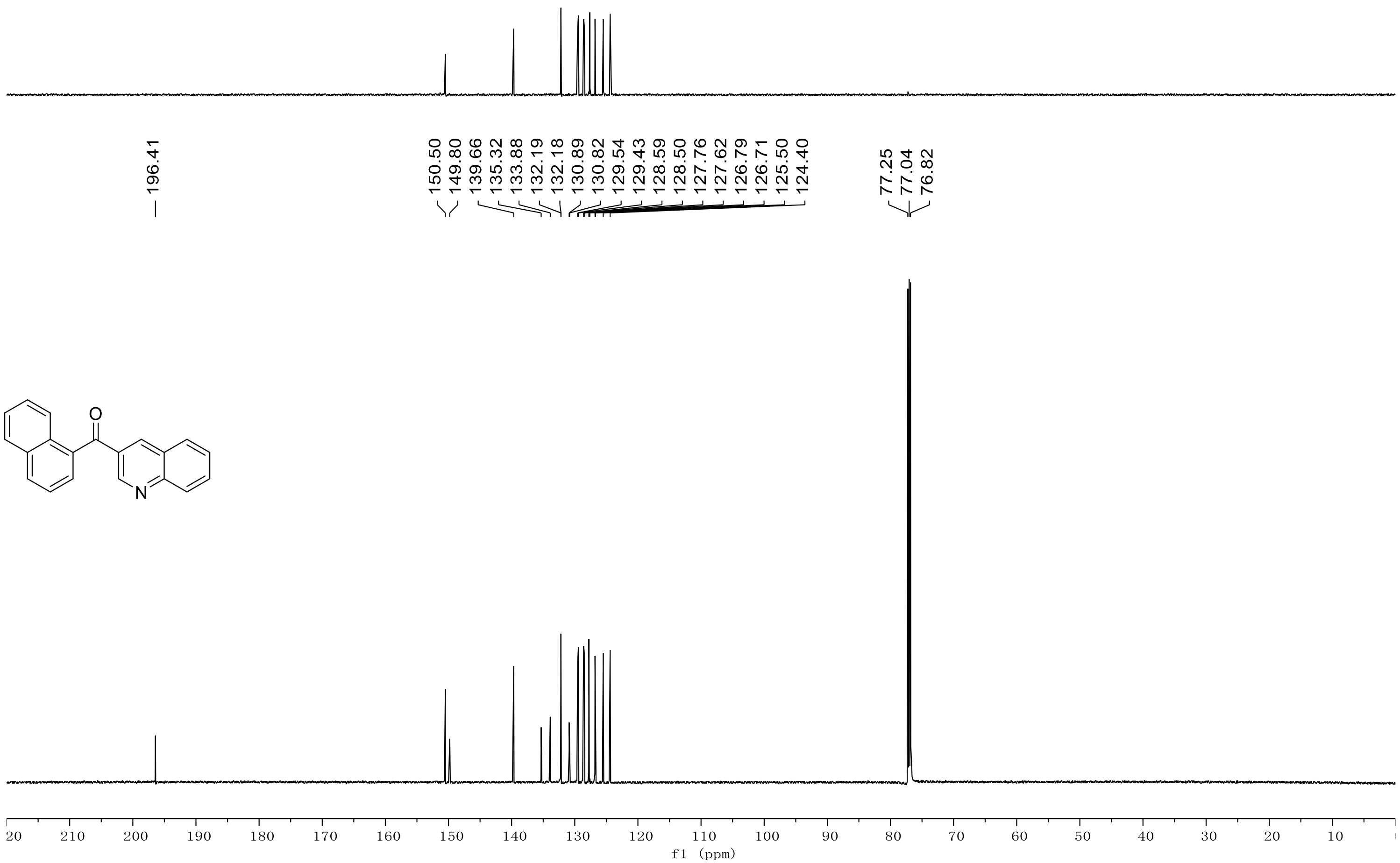

Figure 23. ^{13}C NMR (150 MHz, $CDCl_3$) spectra of compound **3k**

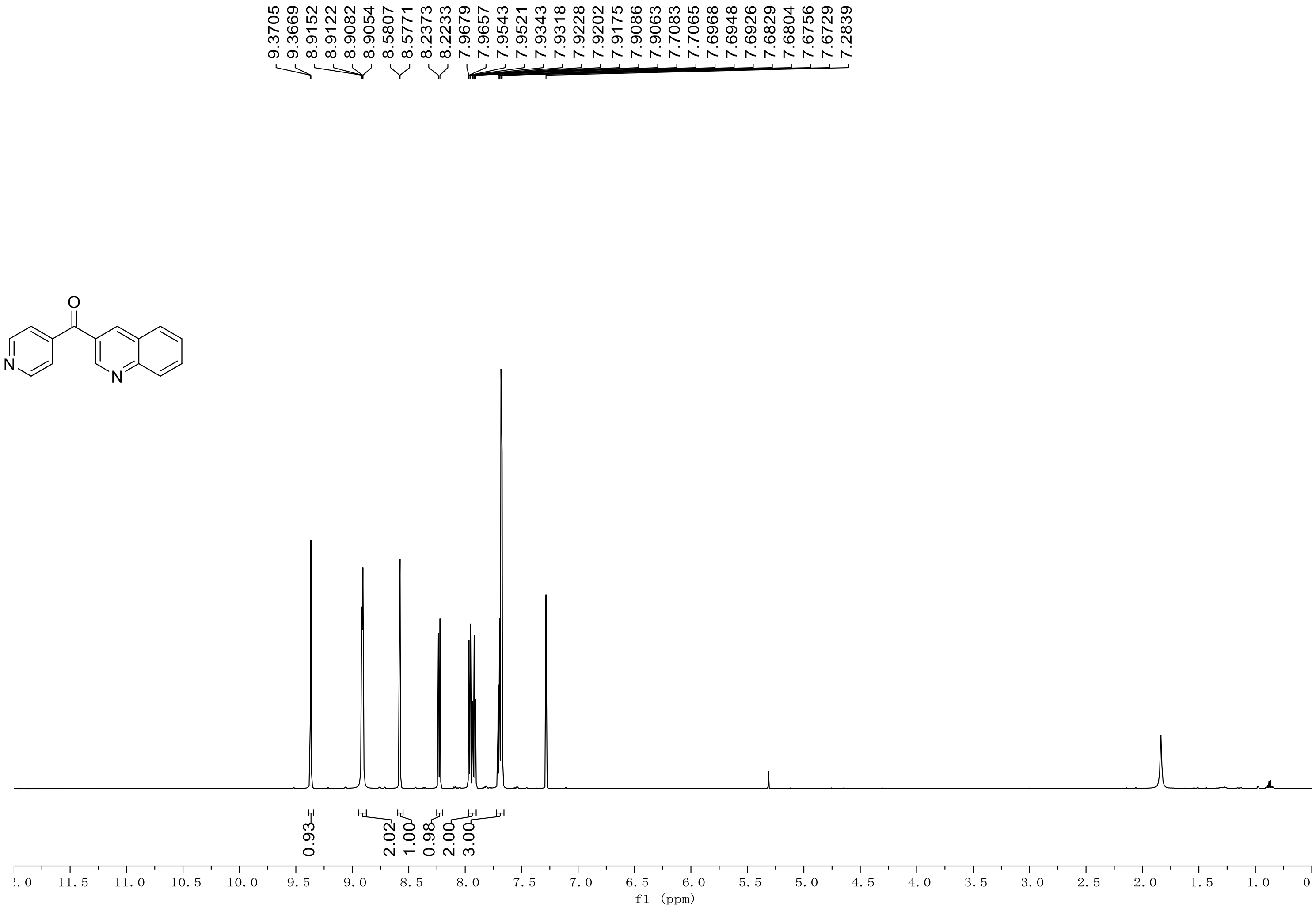

Figure 24. ^{1}H NMR (600 MHz, $CDCl_3$) spectra of compound **3l**

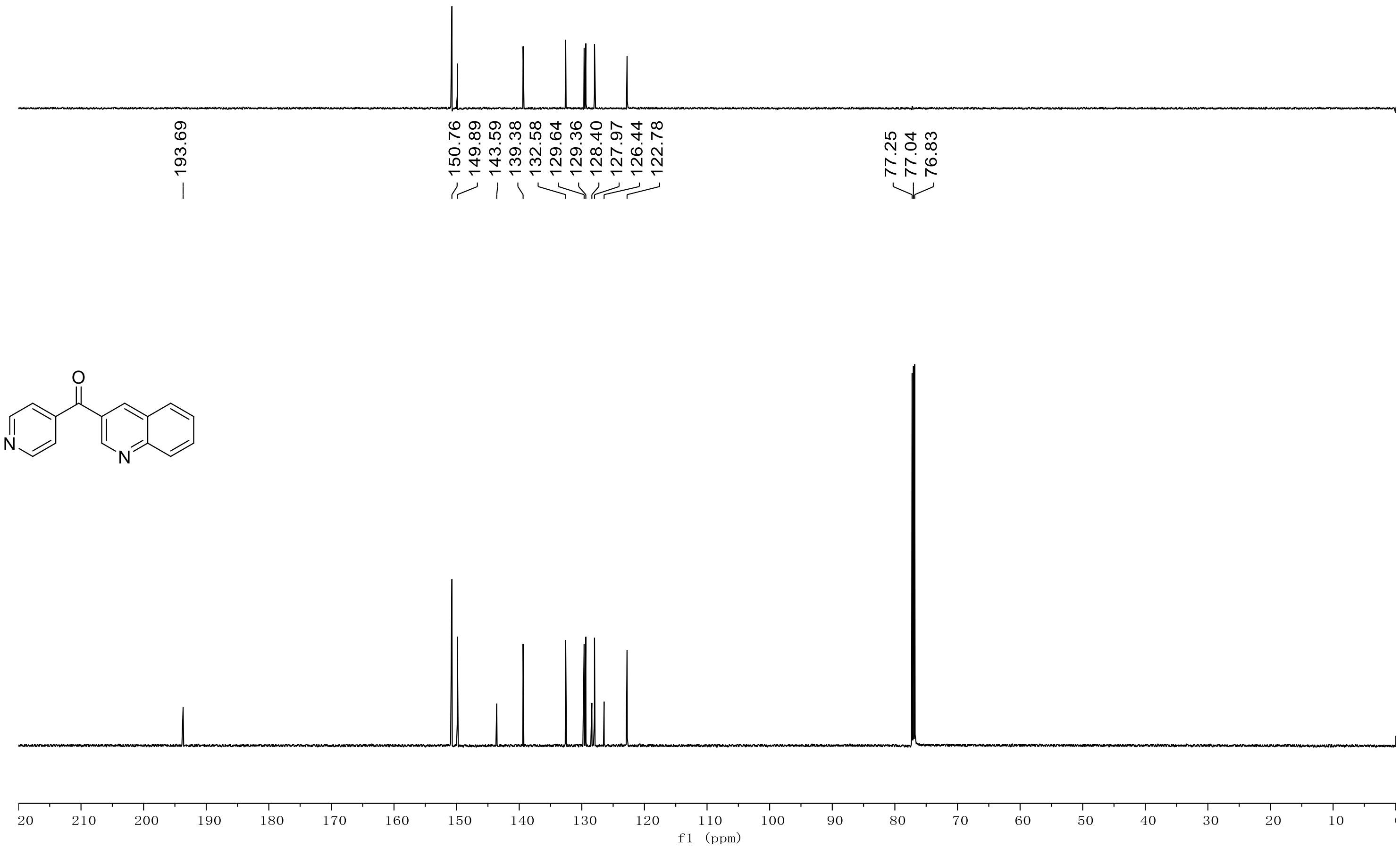

Figure 25. ^{13}C NMR (150 MHz, $CDCl_3$) spectra of compound **3l**

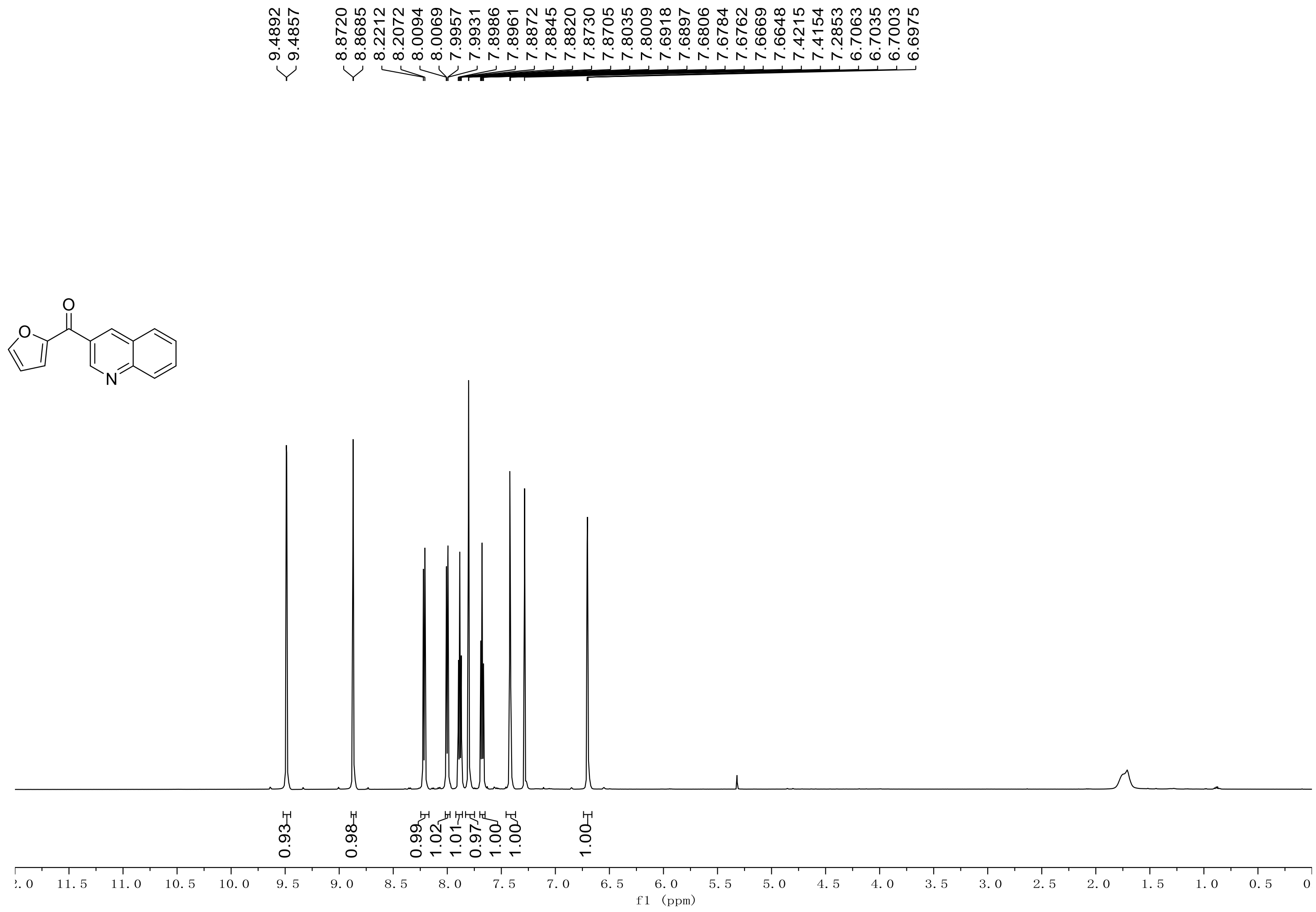

Figure 26. ^{1}H NMR (600 MHz, $CDCl_3$) spectra of compound **3m**

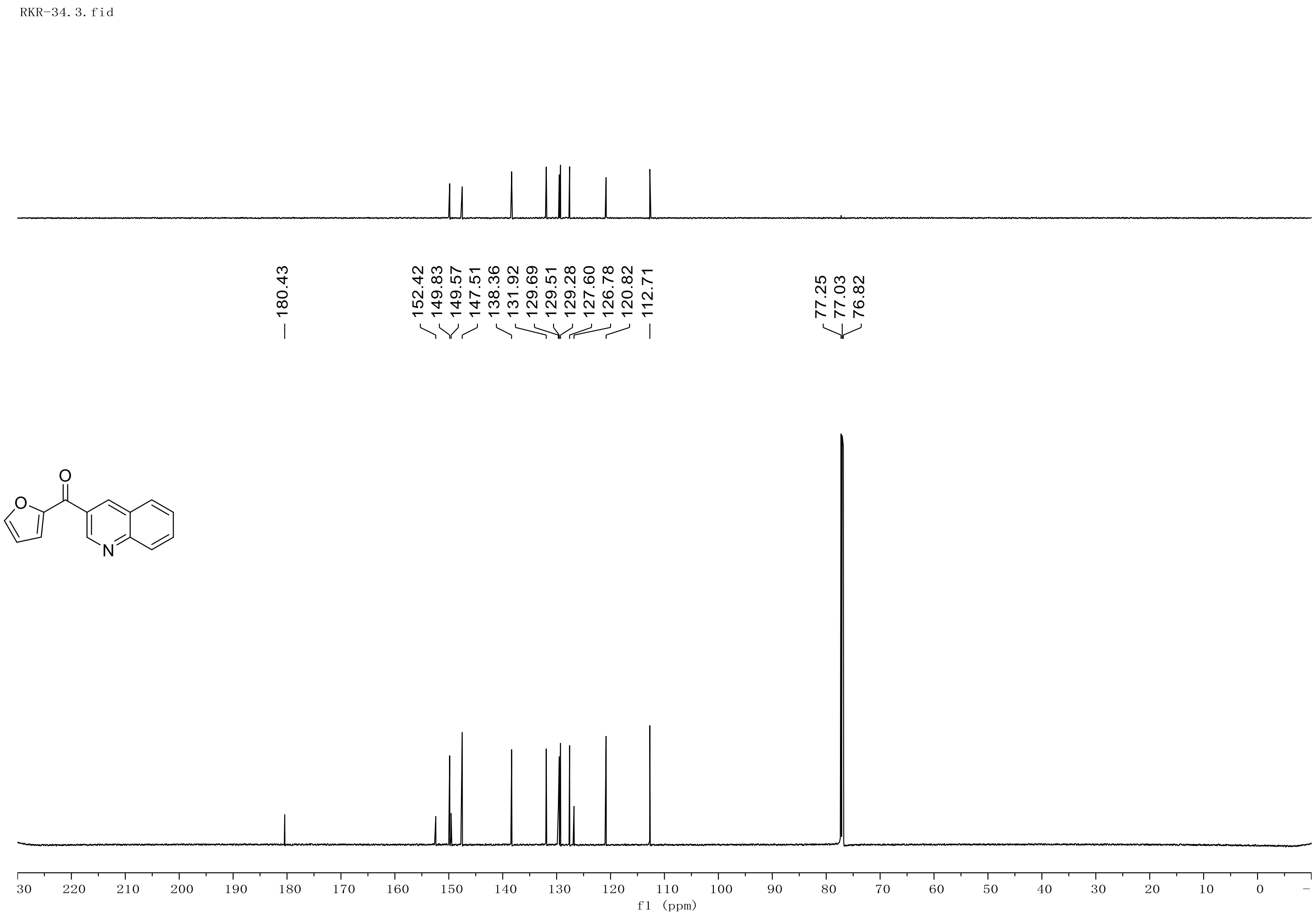

Figure 27. ^{13}C NMR (150 MHz, $CDCl_3$) spectra of compound **3m**

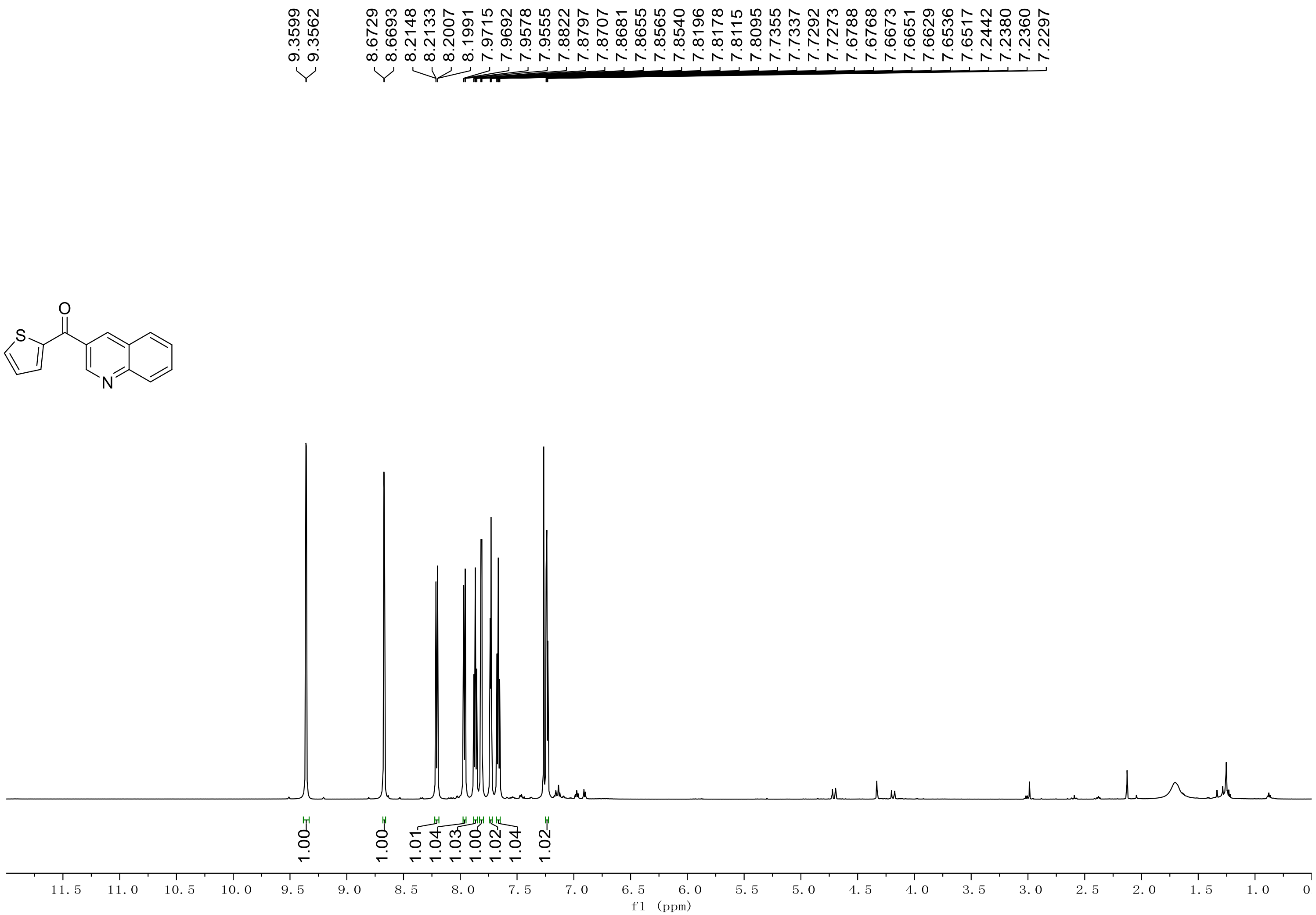

Figure 28. ^{1}H NMR (600 MHz, $CDCl_3$) spectra of compound **3n**

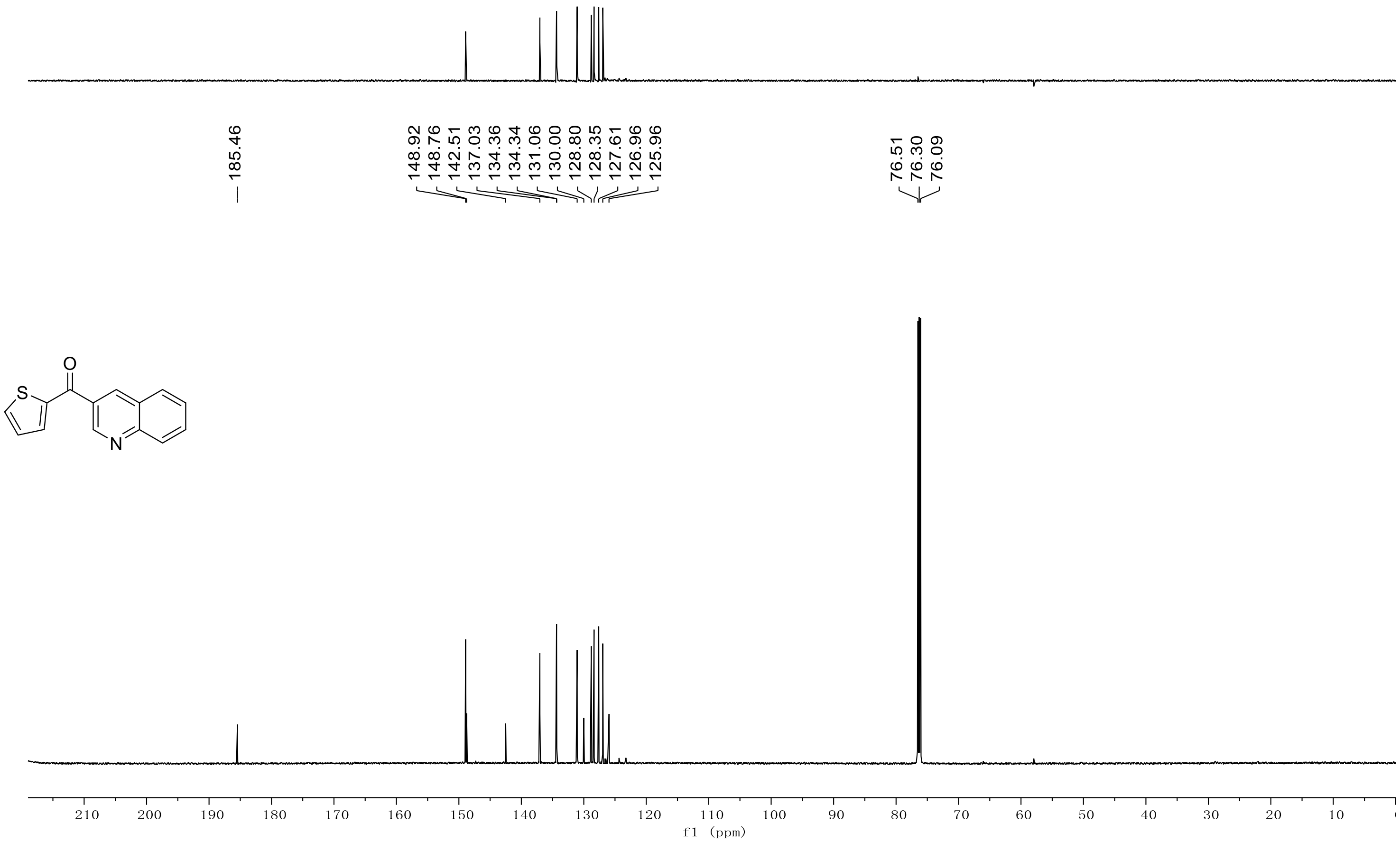

Figure 29. ^{13}C NMR (150 MHz, $CDCl_3$) spectra of compound **3n**

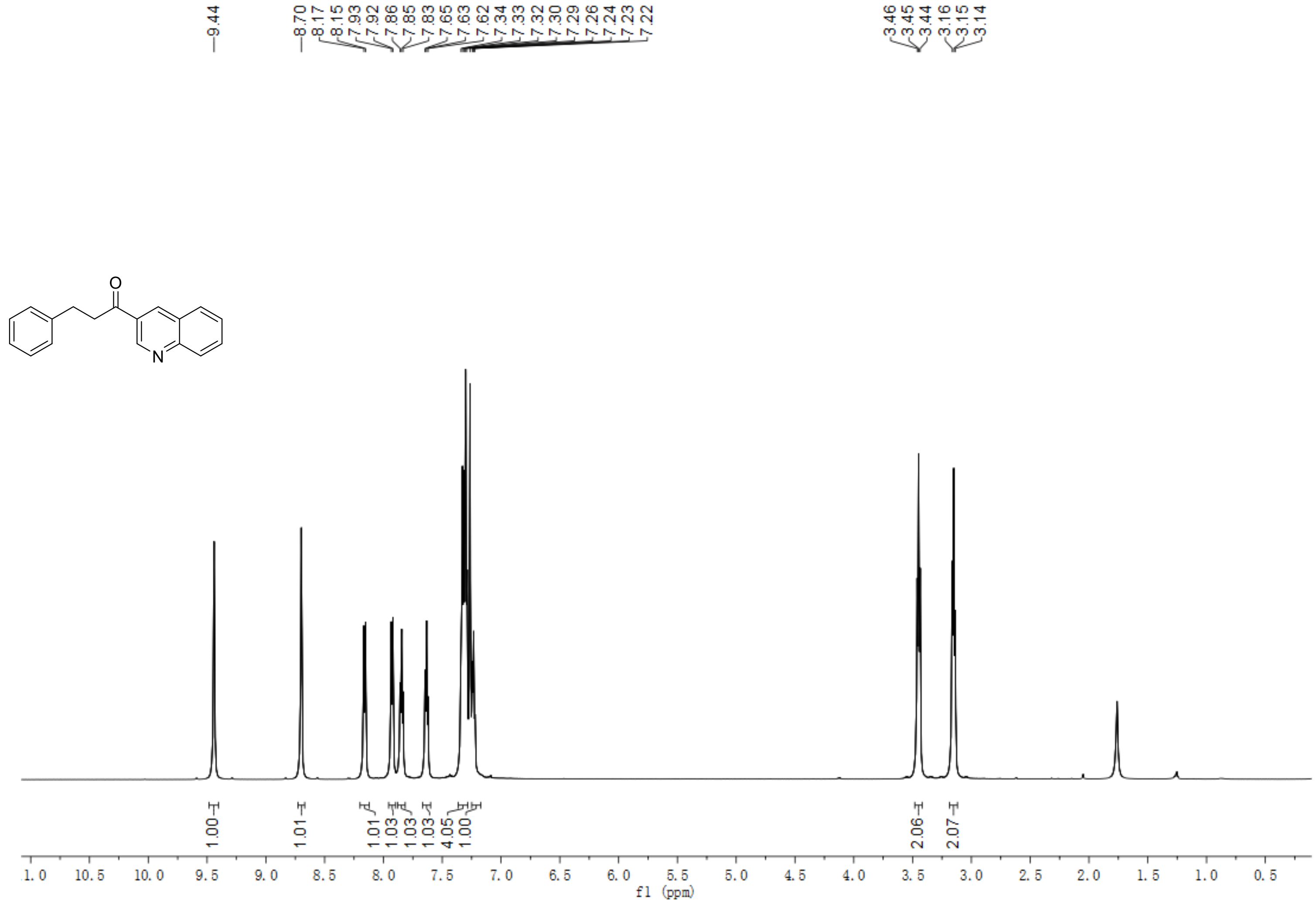

Figure 30. ^{1}H NMR (600 MHz, $CDCl_3$) spectra of compound **3o**

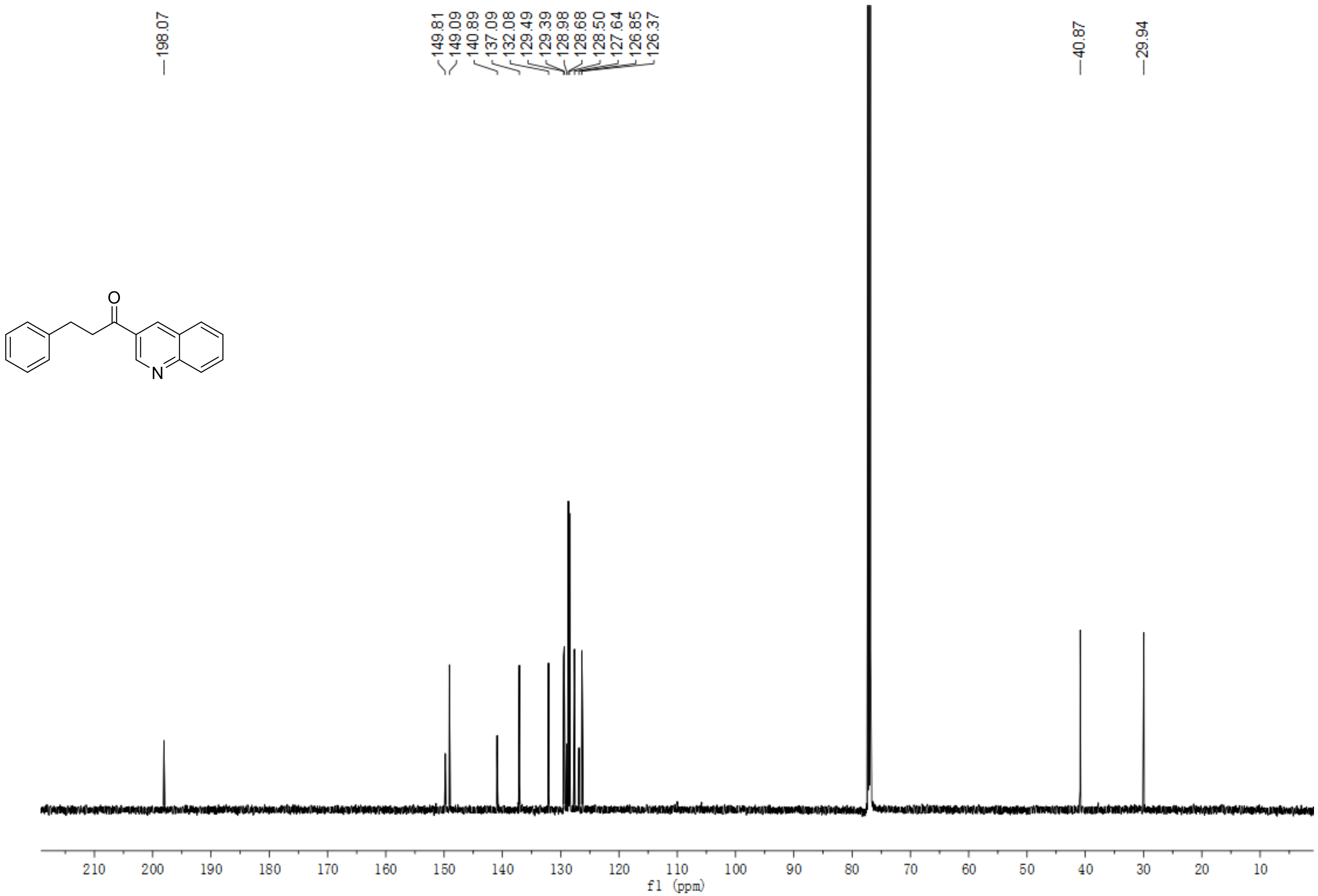

Figure 31. ^{13}C NMR (150 MHz, $CDCl_3$) spectra of compound **3o**

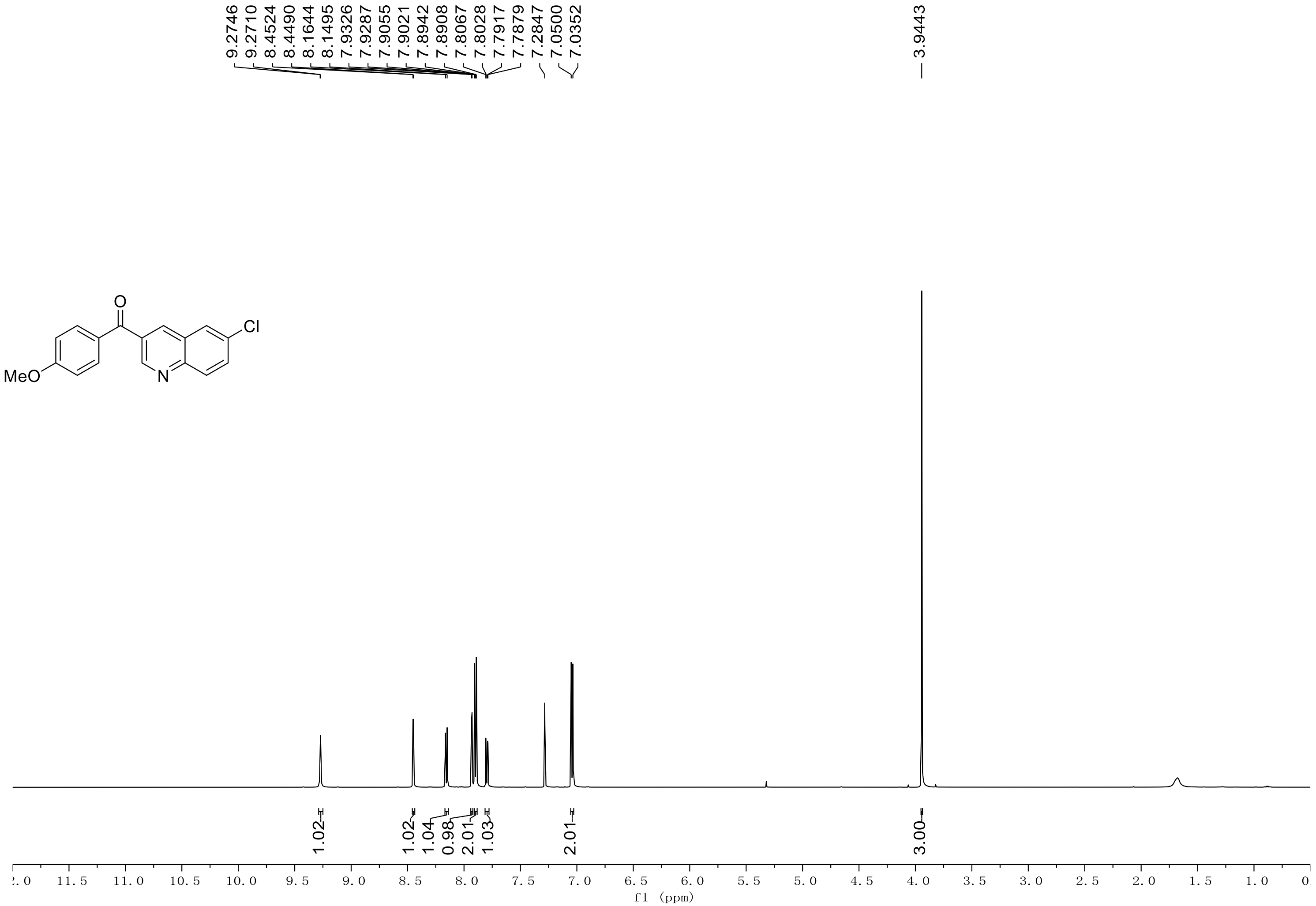

Figure 32. ^{1}H NMR (600 MHz, $CDCl_3$) spectra of compound **3p**

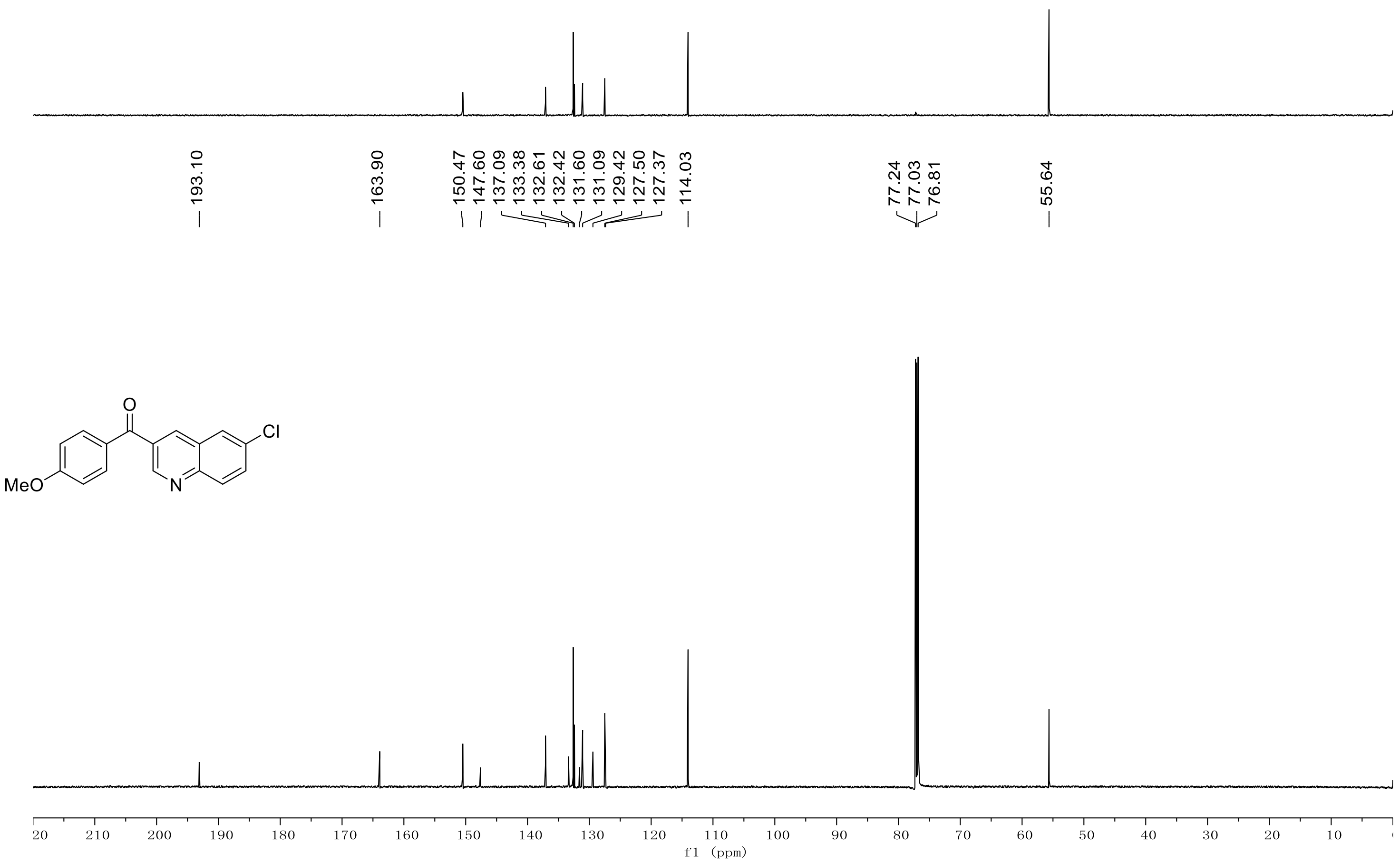

Figure 33. ^{13}C NMR (150 MHz, $CDCl_3$) spectra of compound **3p**

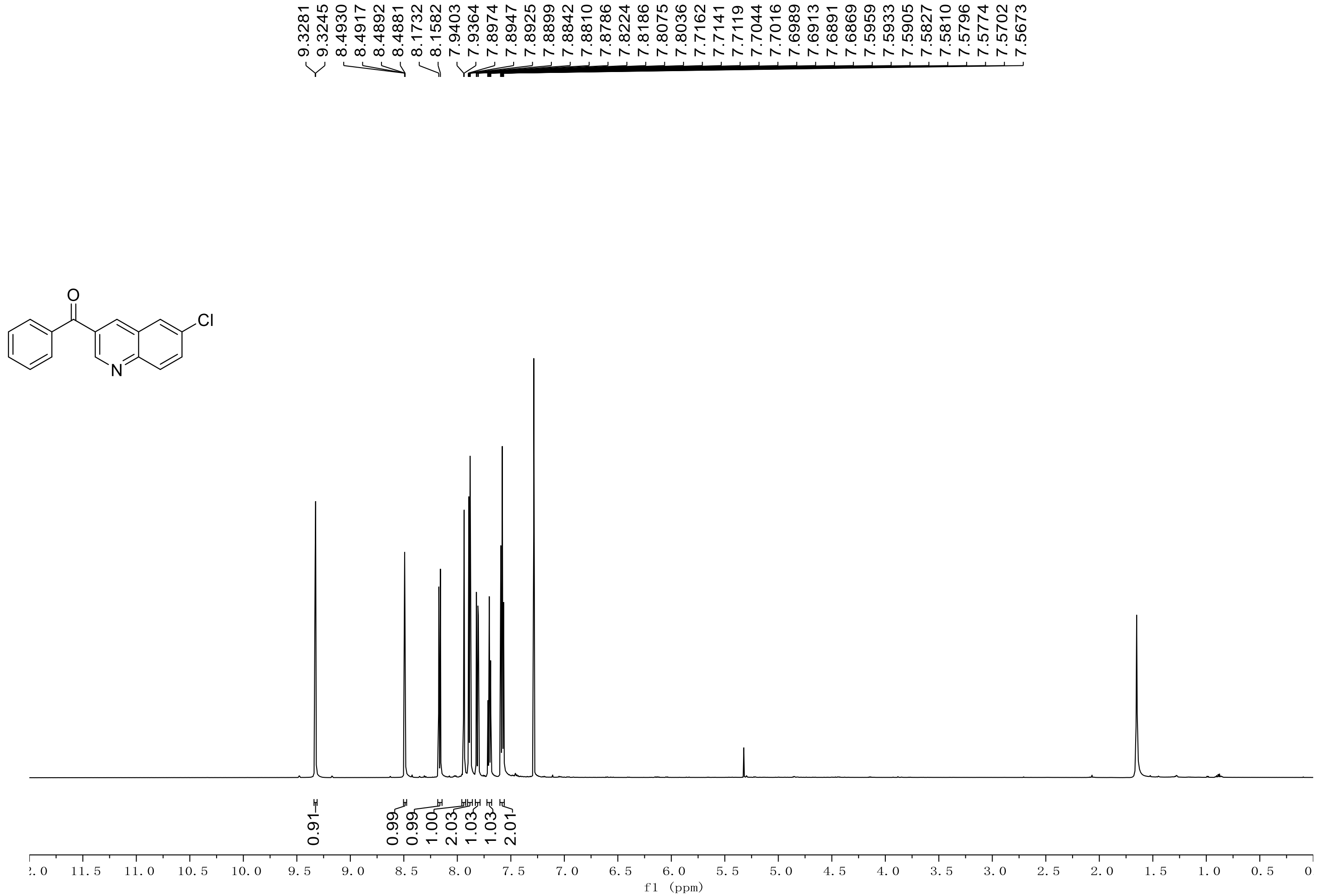

Figure 34. ^{1}H NMR (600 MHz, $CDCl_3$) spectra of compound **3q**

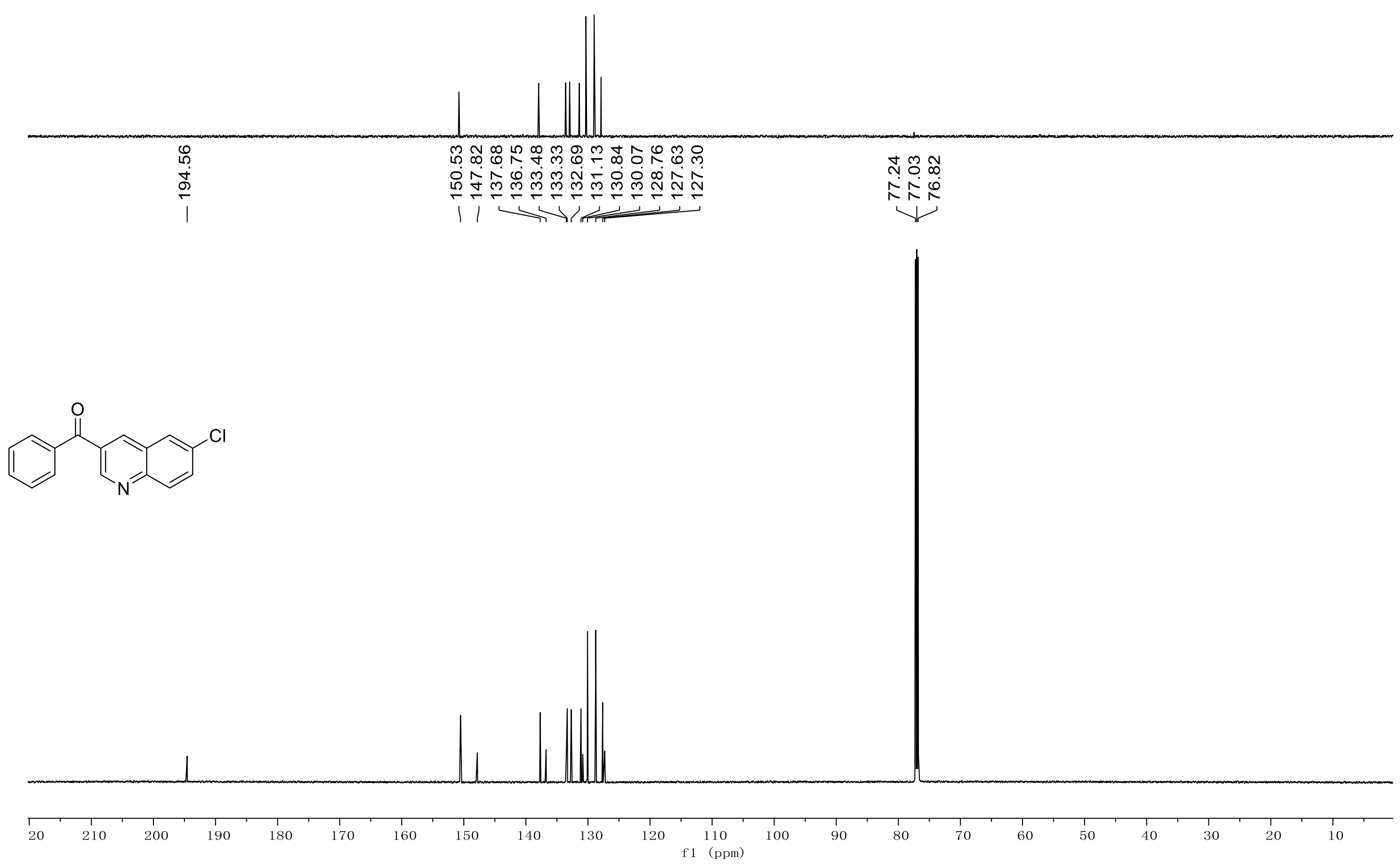

Figure 35. ^{13}C NMR (150 MHz, $CDCl_3$) spectra of compound **3q**

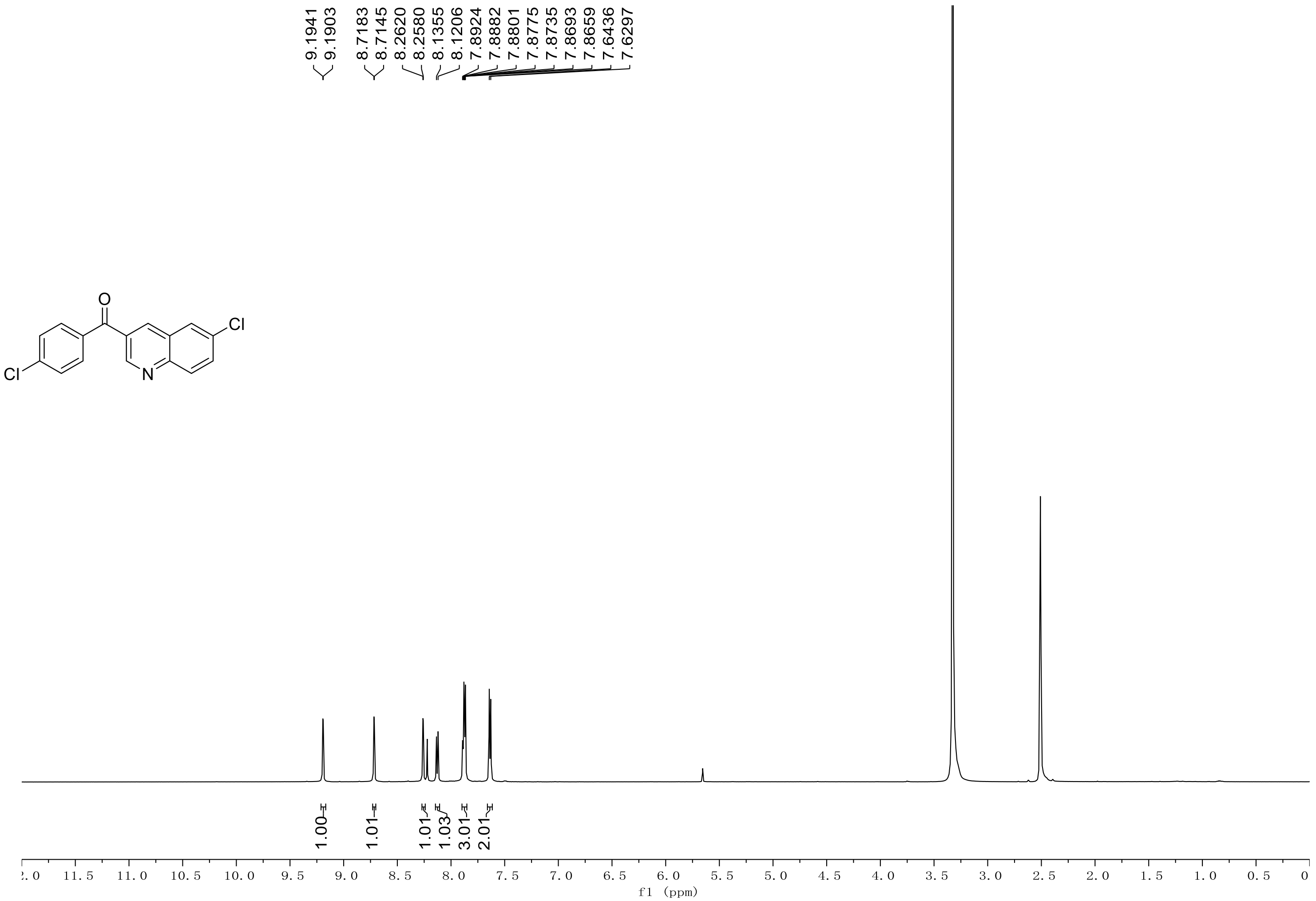

Figure 36. ^{1}H NMR (600 MHz, $CDCl_3$) spectra of compound **3r**

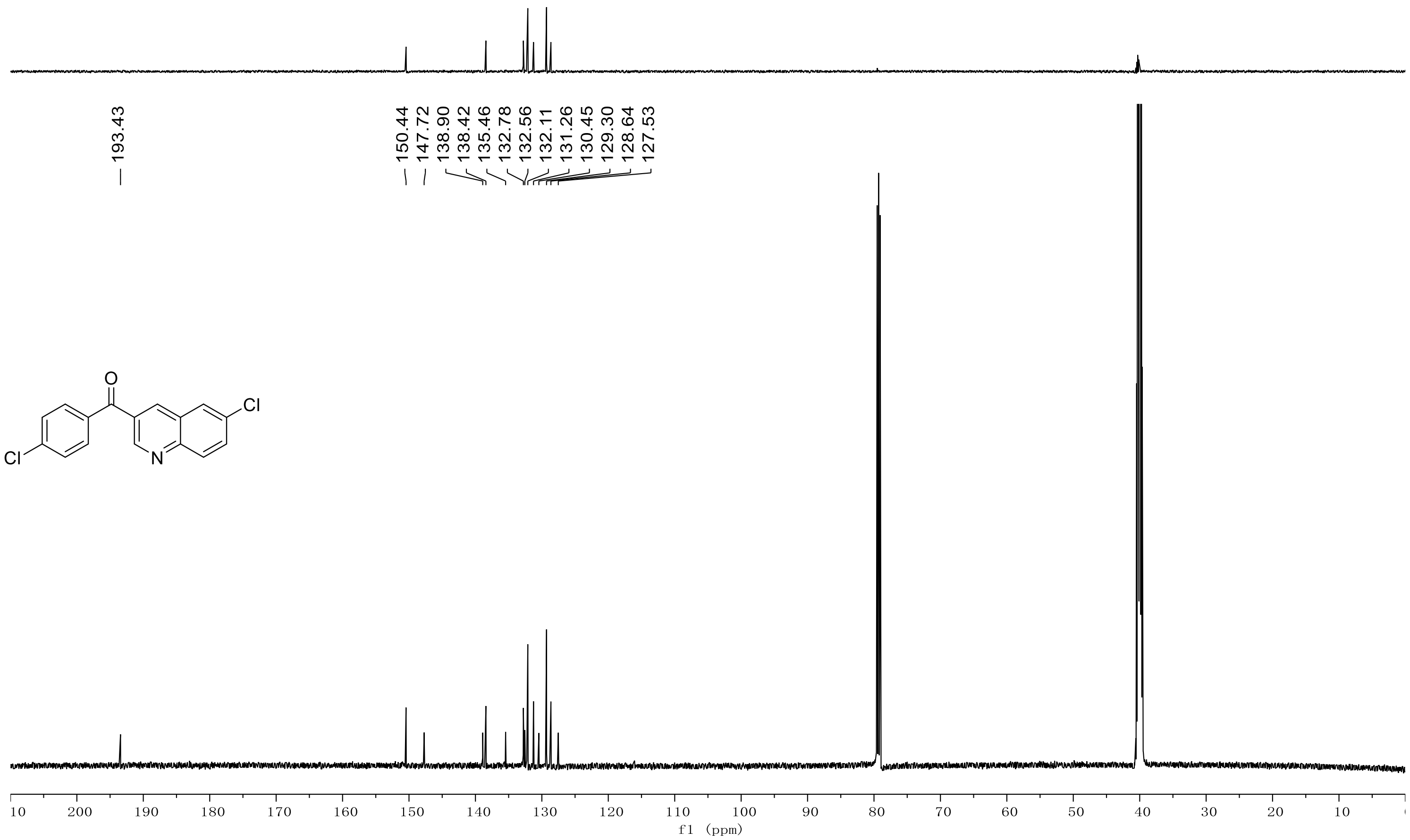

Figure 37. ^{13}C NMR (150 MHz, $CDCl_3$) spectra of compound **3r**

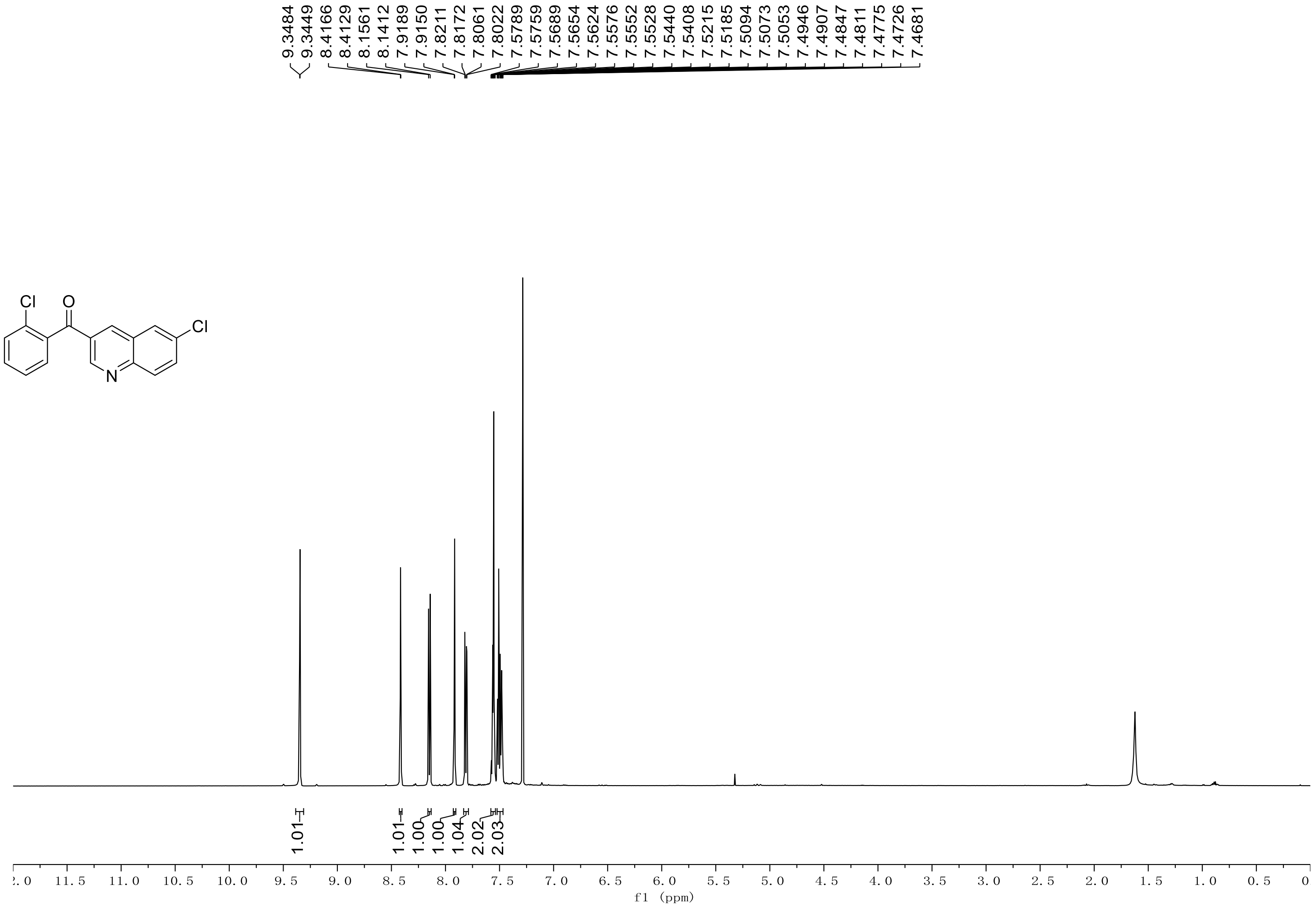

Figure 38. ^{1}H NMR (600 MHz, $CDCl_3$) spectra of compound **3s**

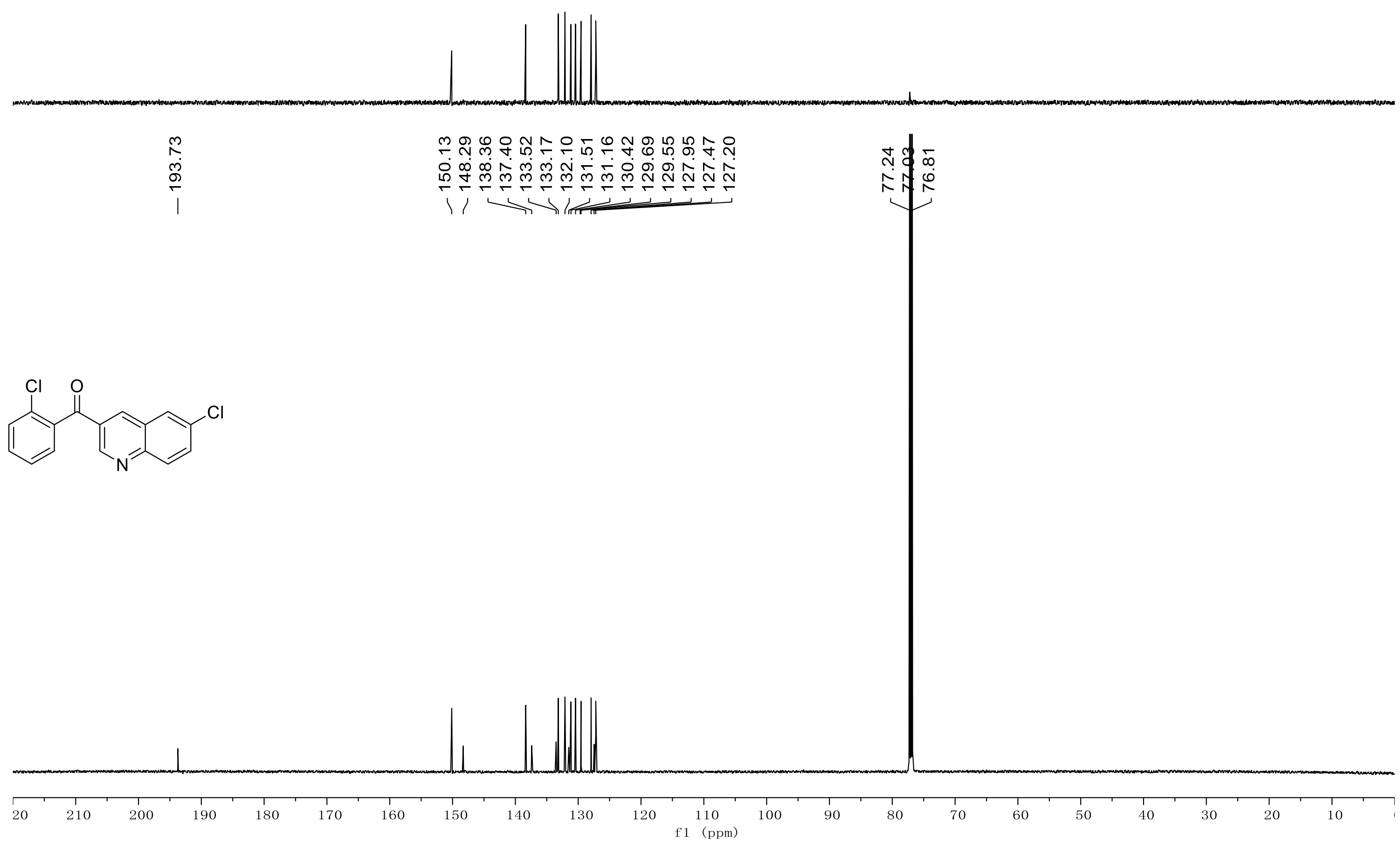

Figure 39. ^{13}C NMR (150 MHz, $CDCl_3$) spectra of compound **3s**

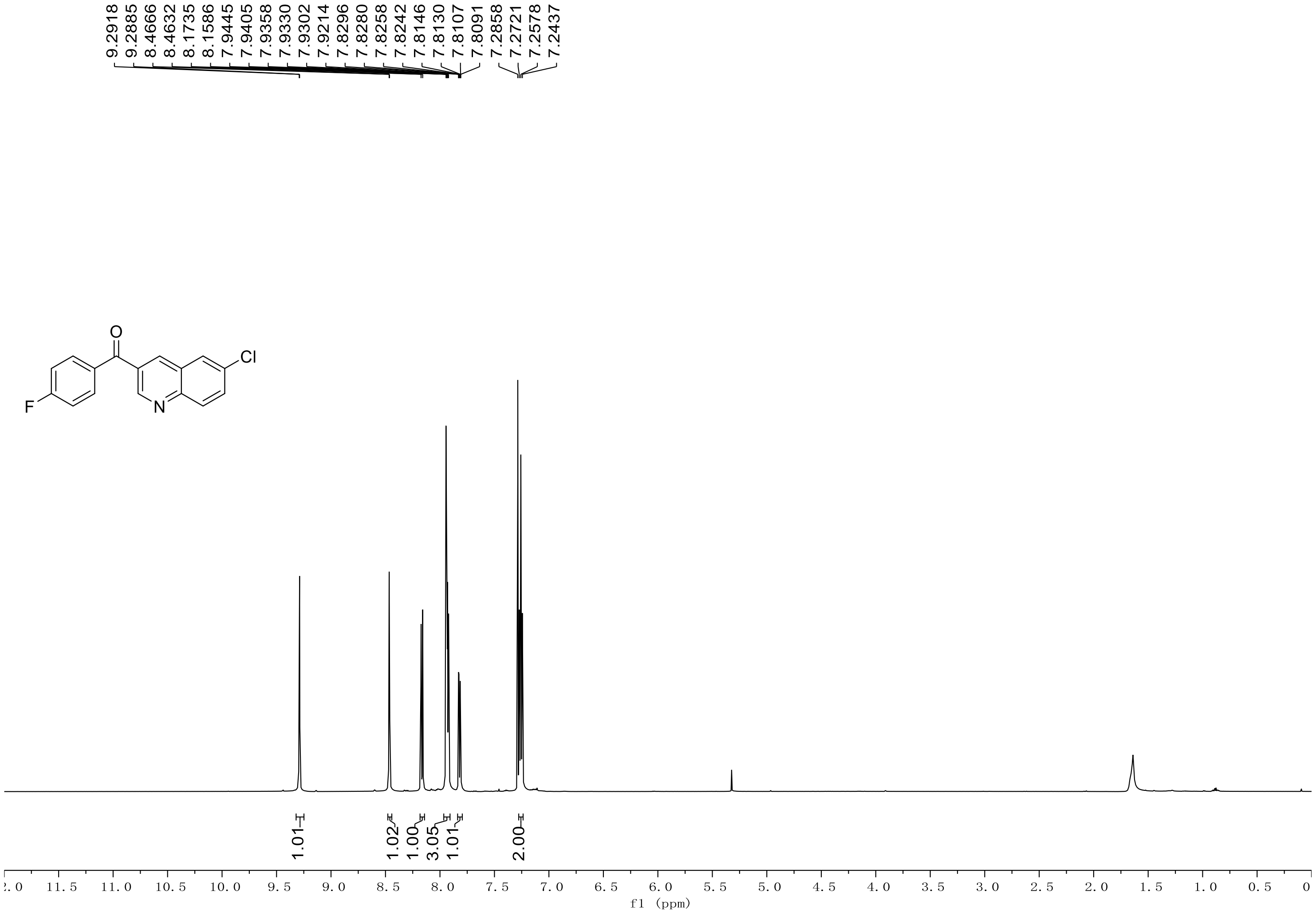

Figure 40. ^{1}H NMR (600 MHz, $CDCl_3$) spectra of compound **3t**

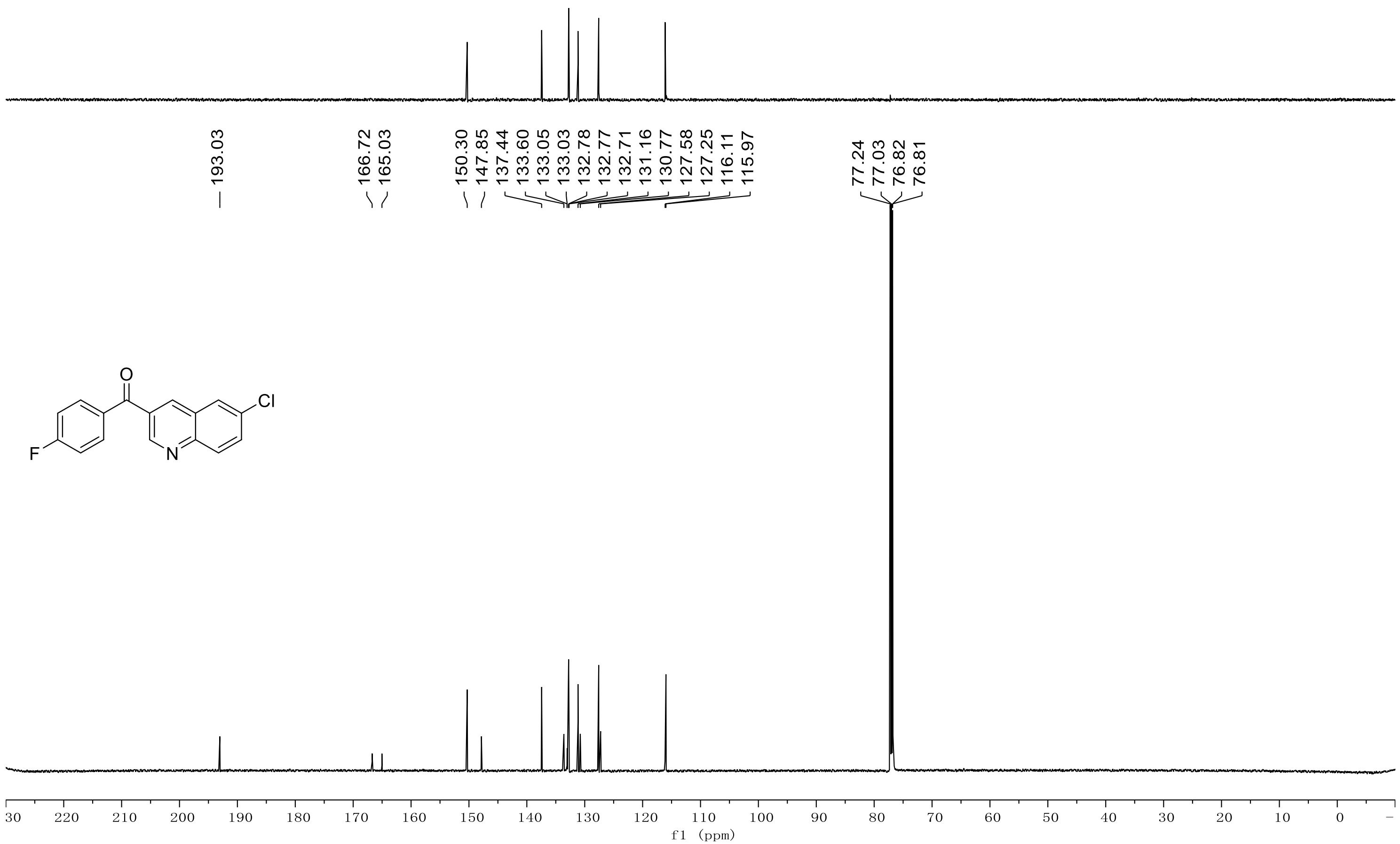

Figure 41. ^{13}C NMR (150 MHz, $CDCl_3$) spectra of compound **3t**

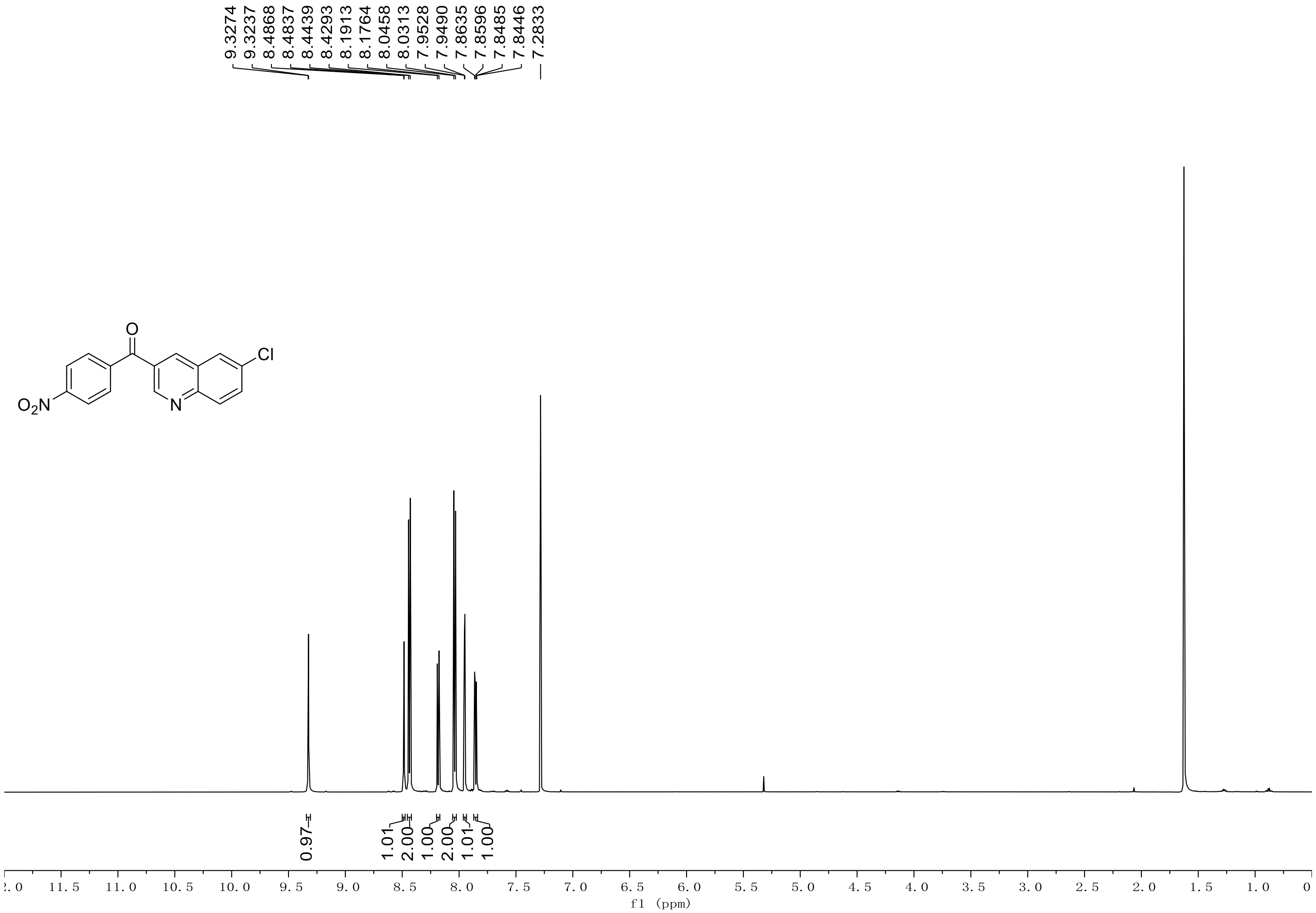

Figure 42. ^{1}H NMR (600 MHz, $CDCl_3$) spectra of compound **3u**

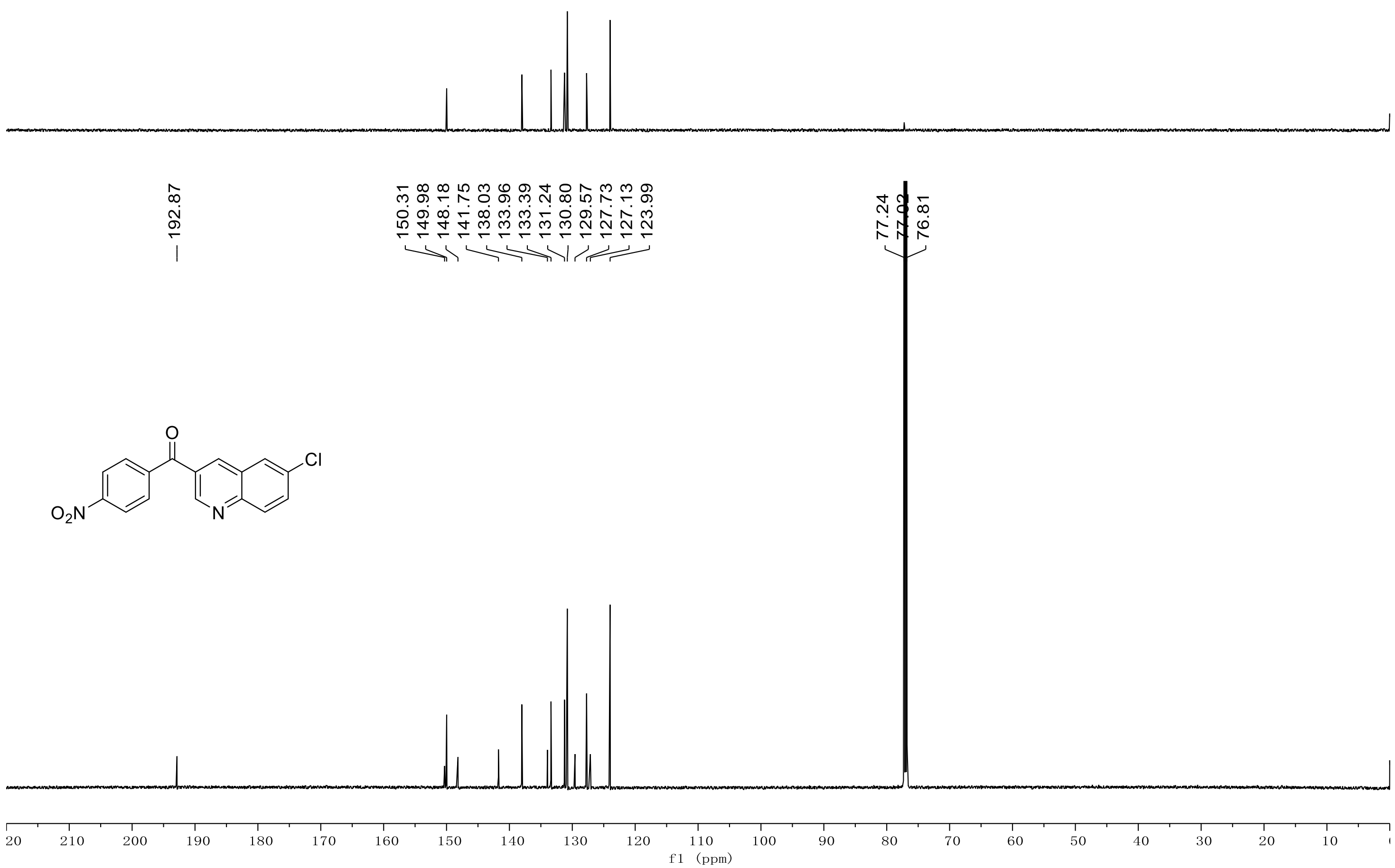

Figure 43. ^{13}C NMR (150 MHz, $CDCl_3$) spectra of compound **3u**

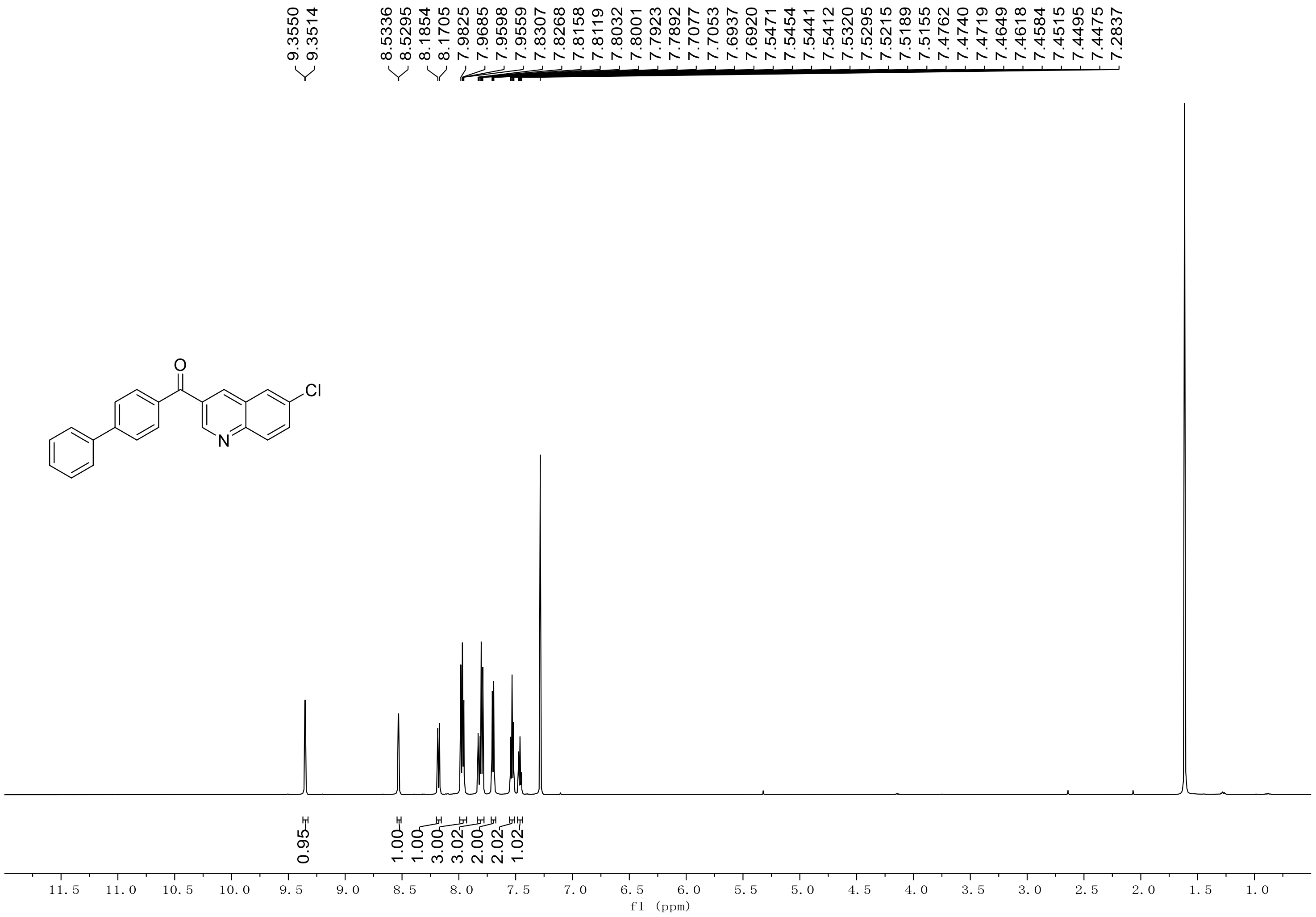

Figure 44. ^{1}H NMR (600 MHz, $CDCl_3$) spectra of compound **3v**

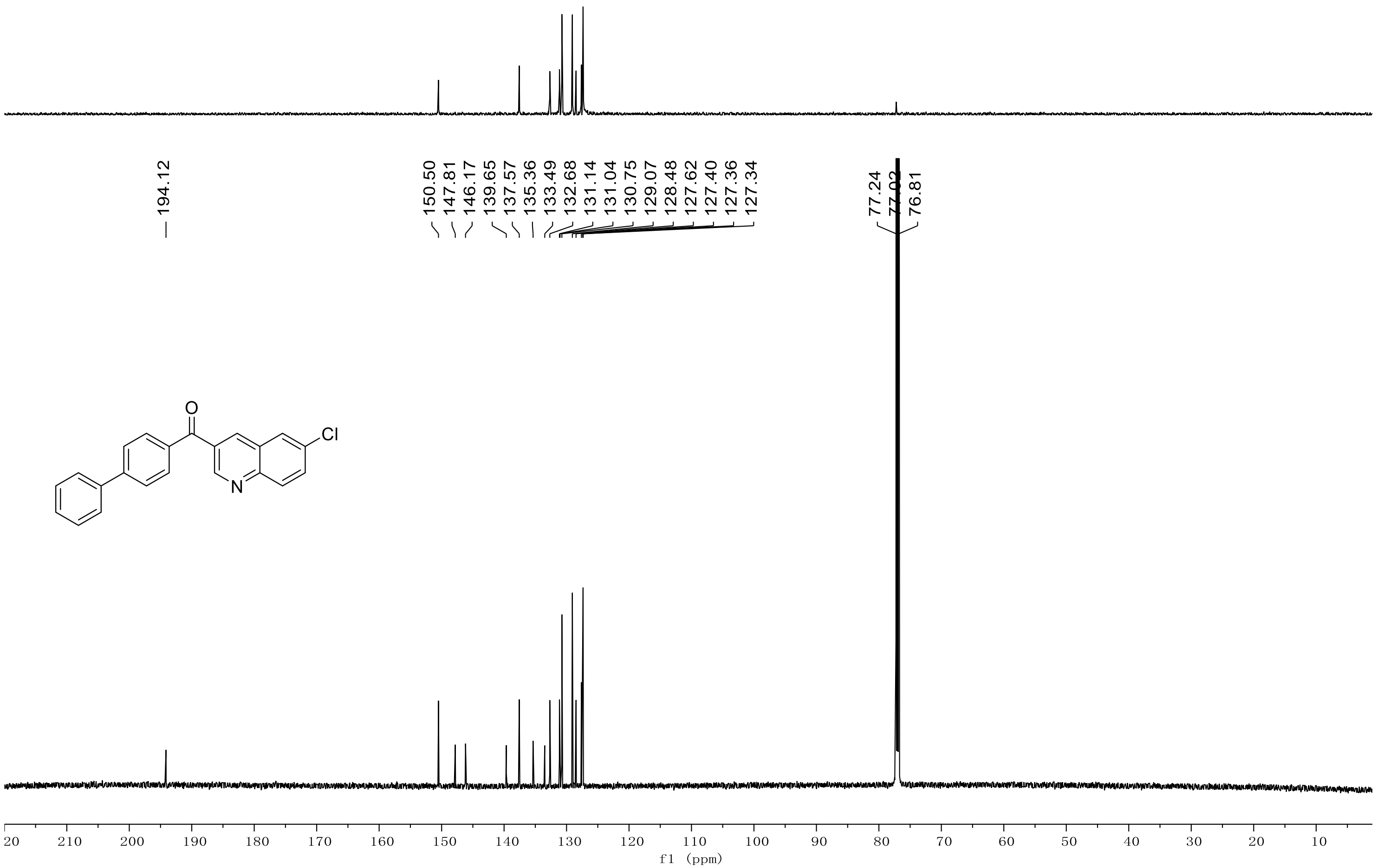

Figure 45. ^{13}C NMR (150 MHz, $CDCl_3$) spectra of compound **3v**

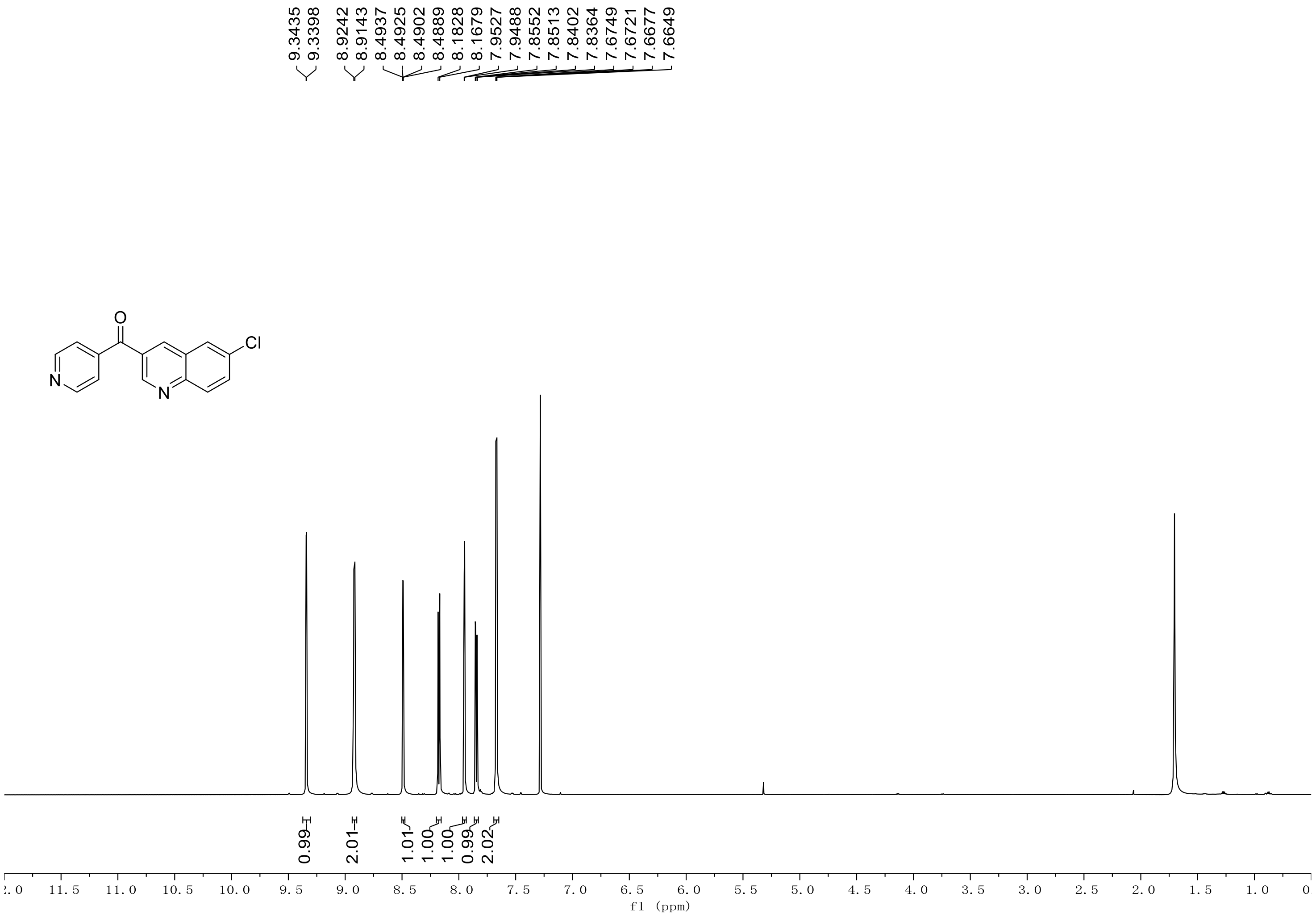

Figure 46. ^{1}H NMR (600 MHz, $CDCl_3$) spectra of compound **3w**

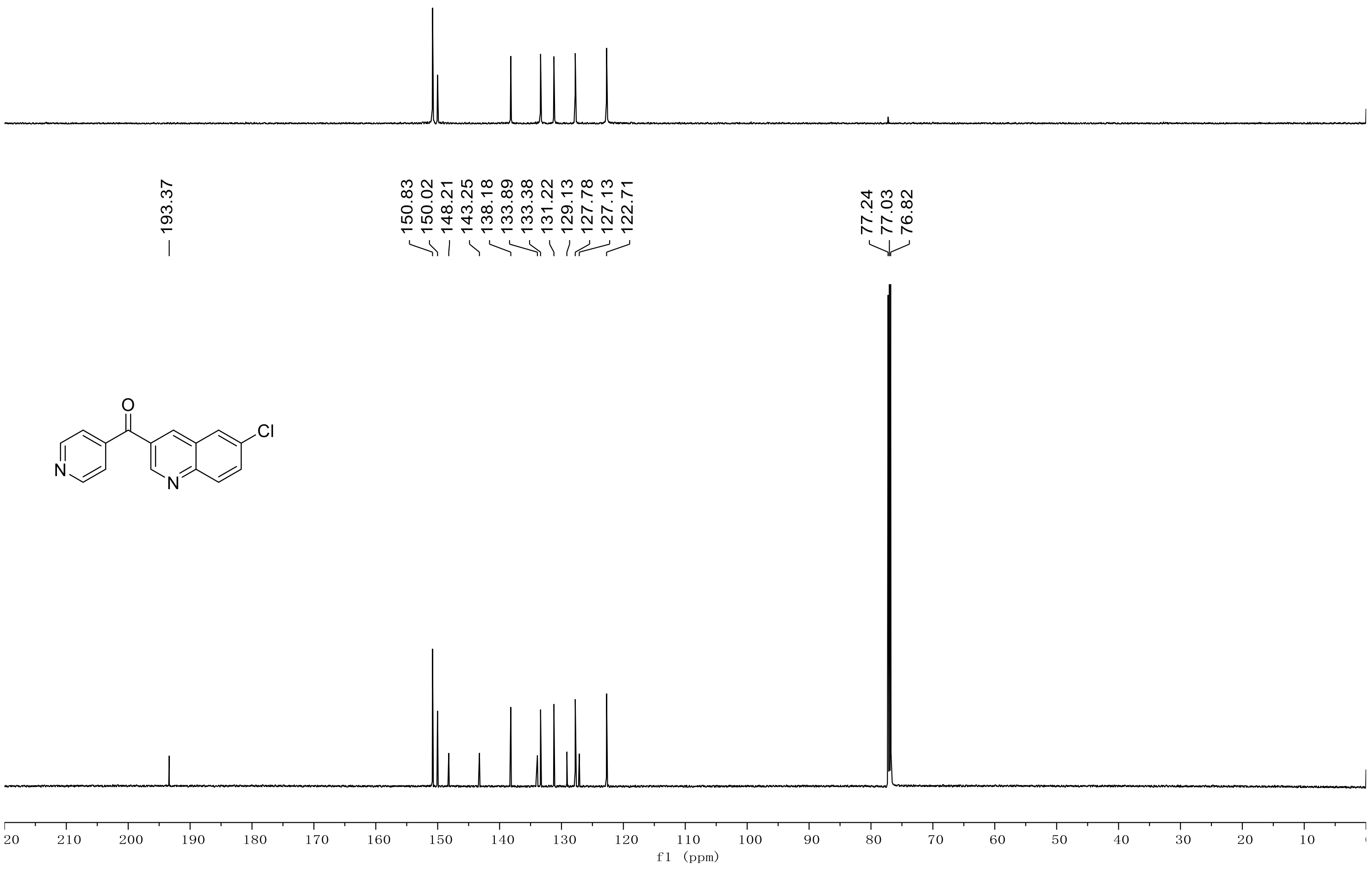

Figure 47. ^{13}C NMR (150 MHz, $CDCl_3$) spectra of compound **3w**

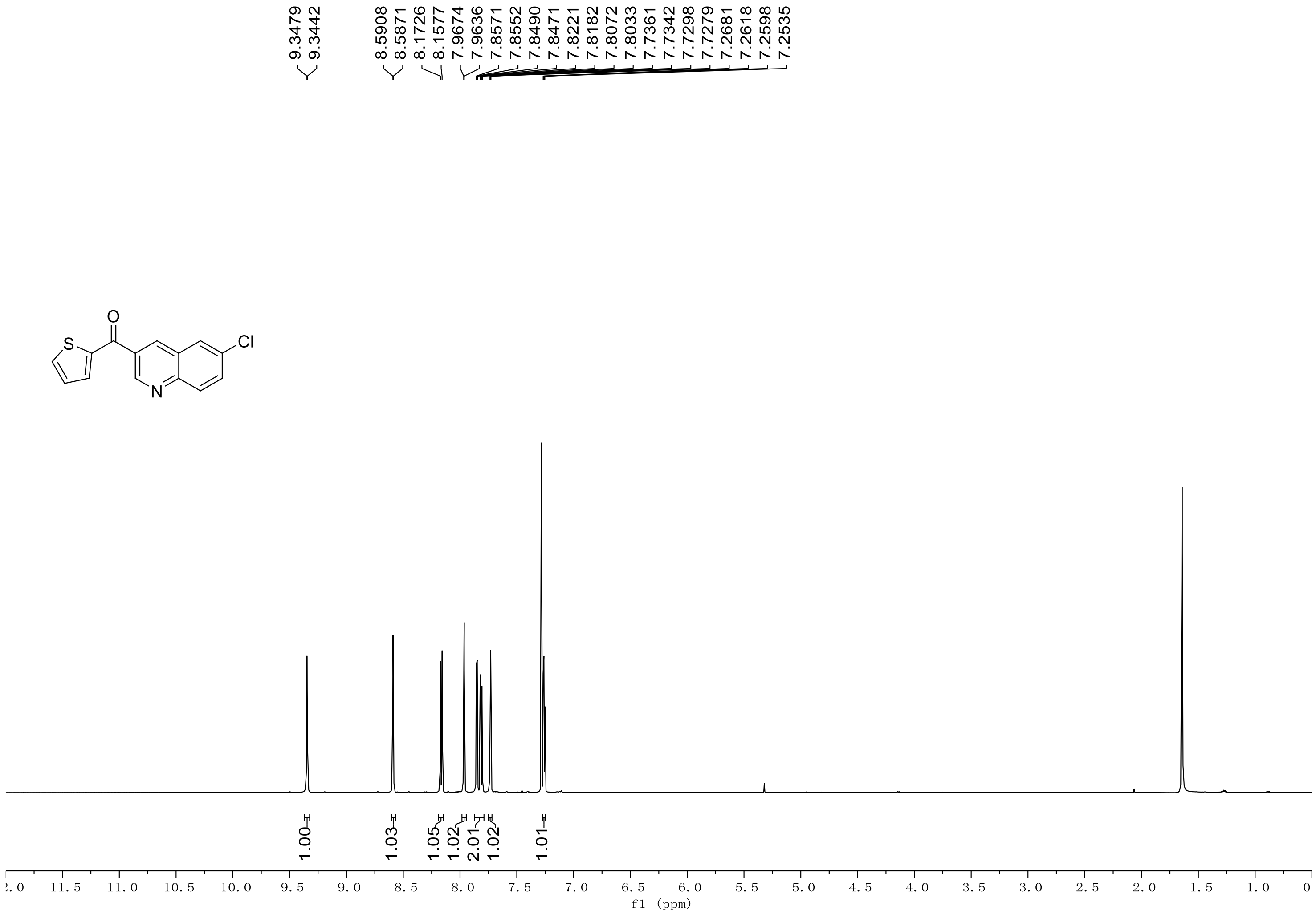

Figure 48. ^{1}H NMR (600 MHz, $CDCl_3$) spectra of compound **3x**

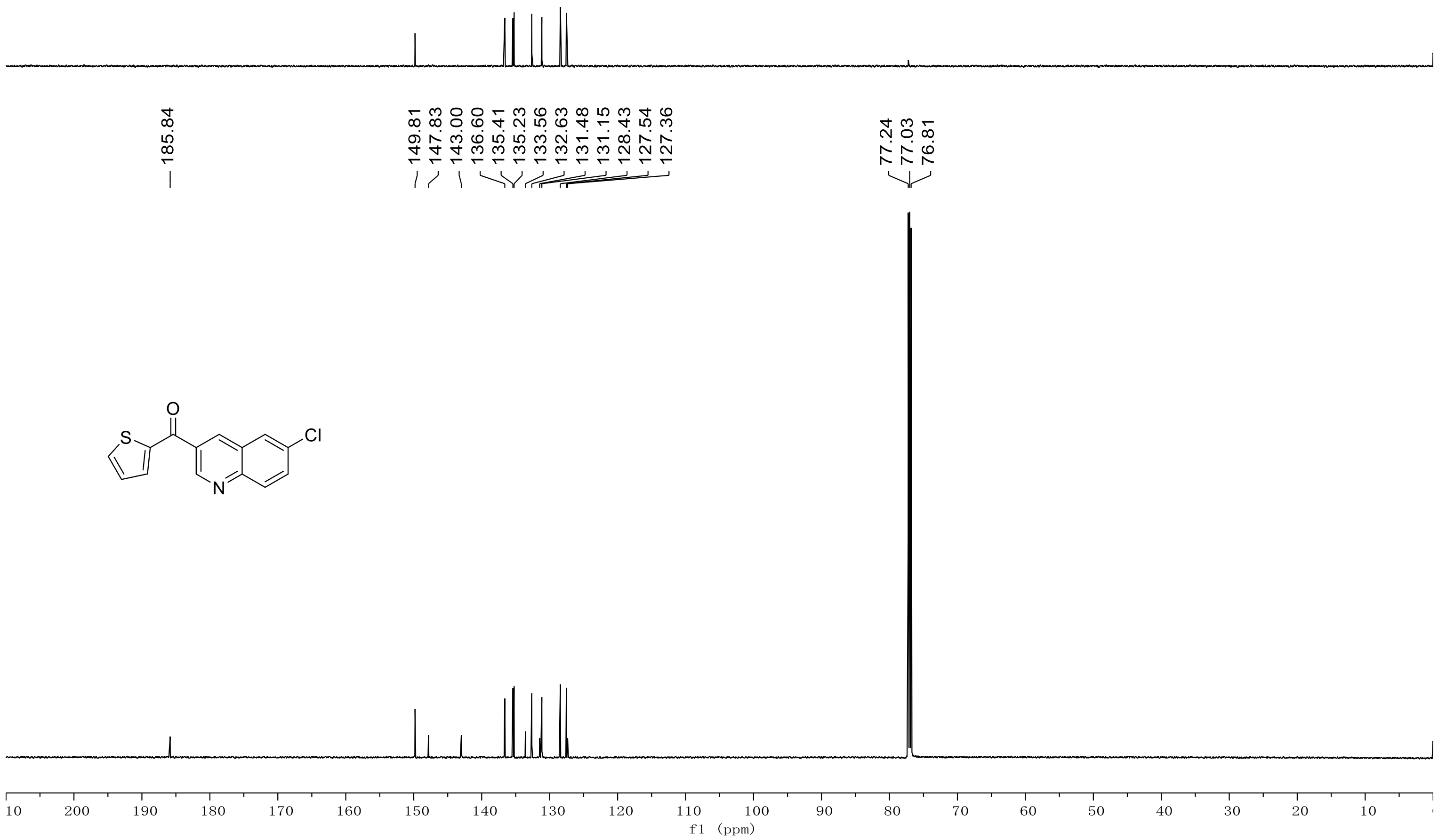

Figure 49. ^{13}C NMR (150 MHz, $CDCl_3$) spectra of compound **3x**

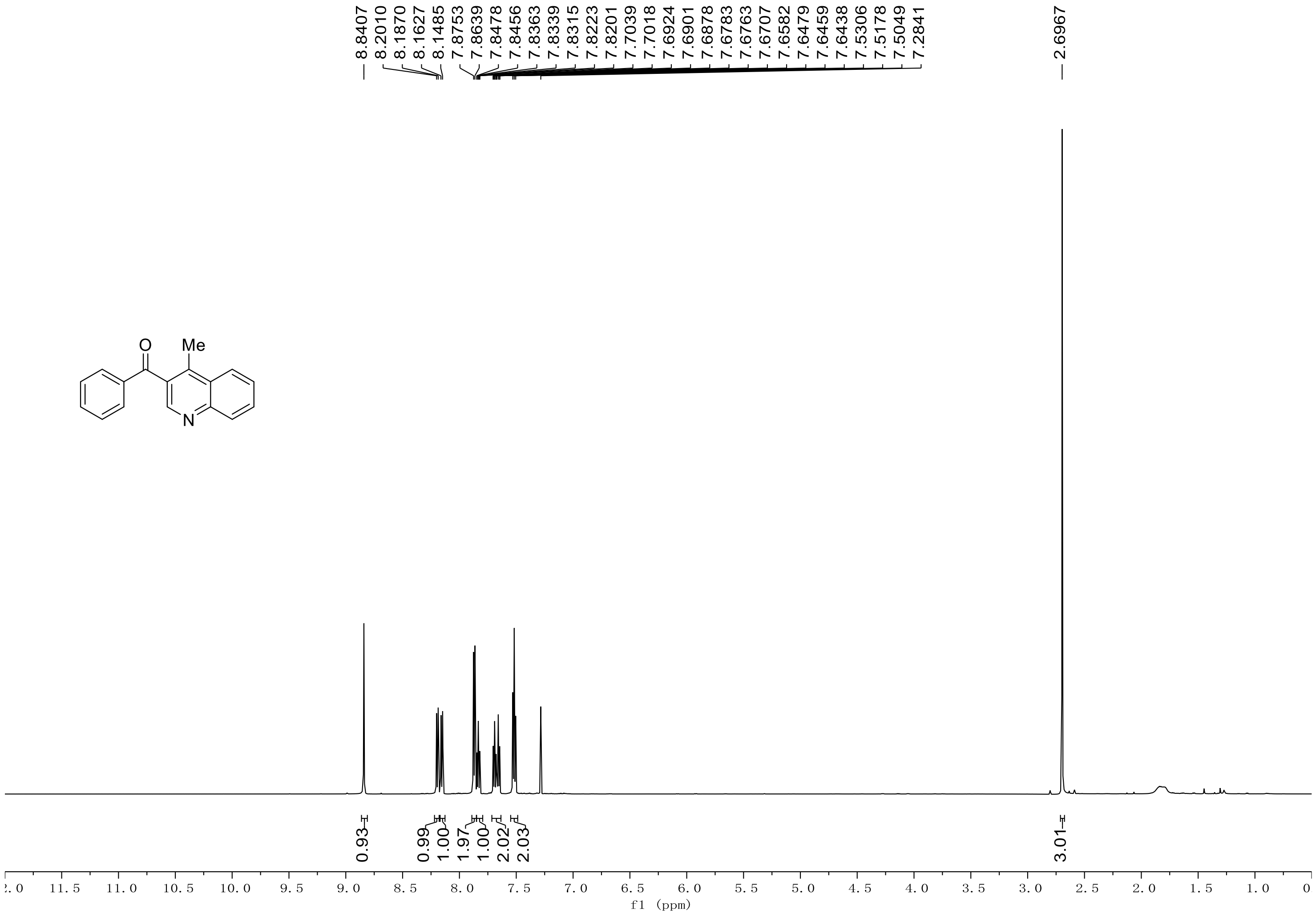

Figure 50. ^{1}H NMR (600 MHz, $CDCl_3$) spectra of compound **3y**

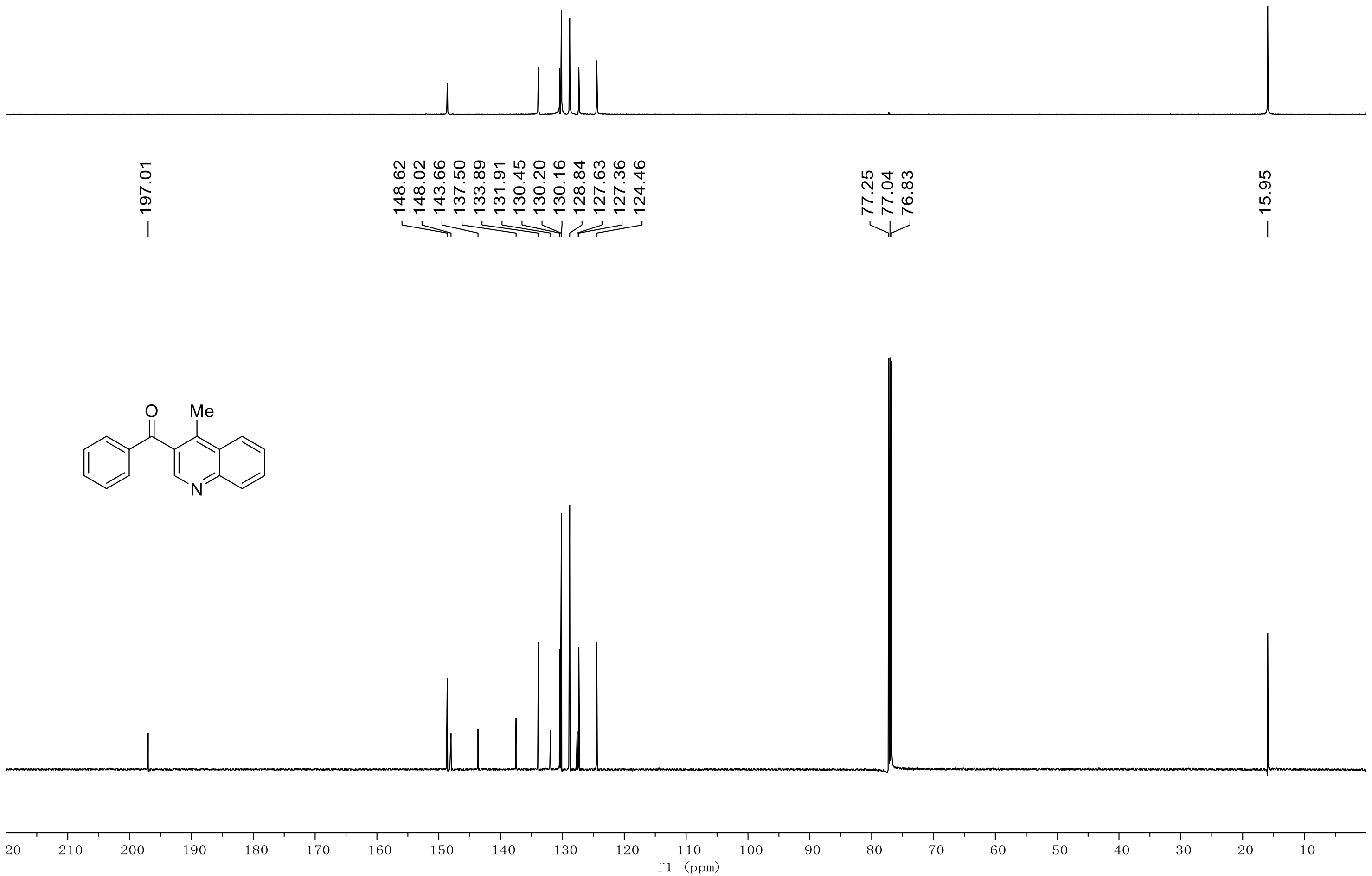

Figure 51. ^{13}C NMR (150 MHz, $CDCl_3$) spectra of compound **3y**

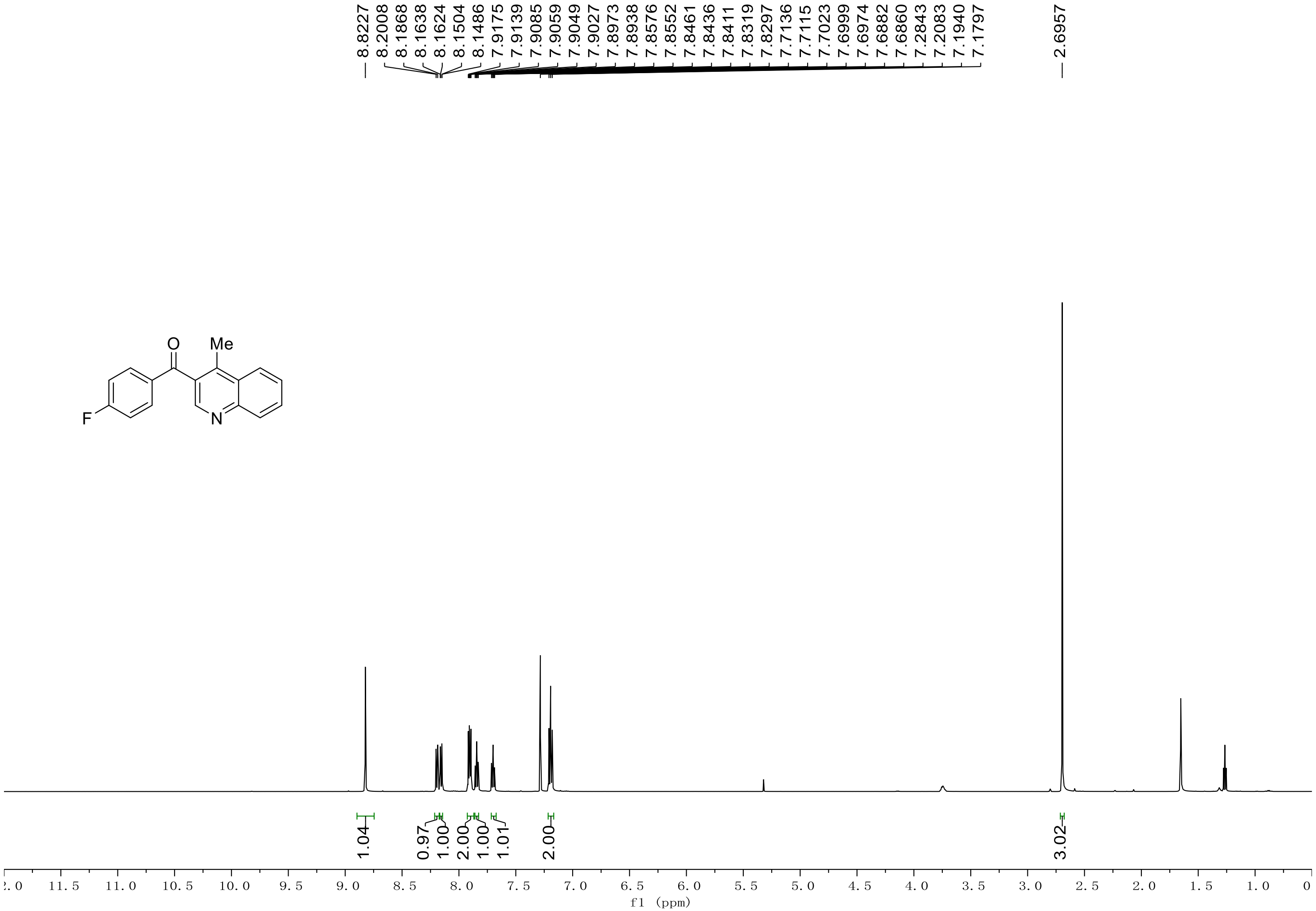

Figure 52. ^{1}H NMR (600 MHz, $CDCl_3$) spectra of compound **3z**

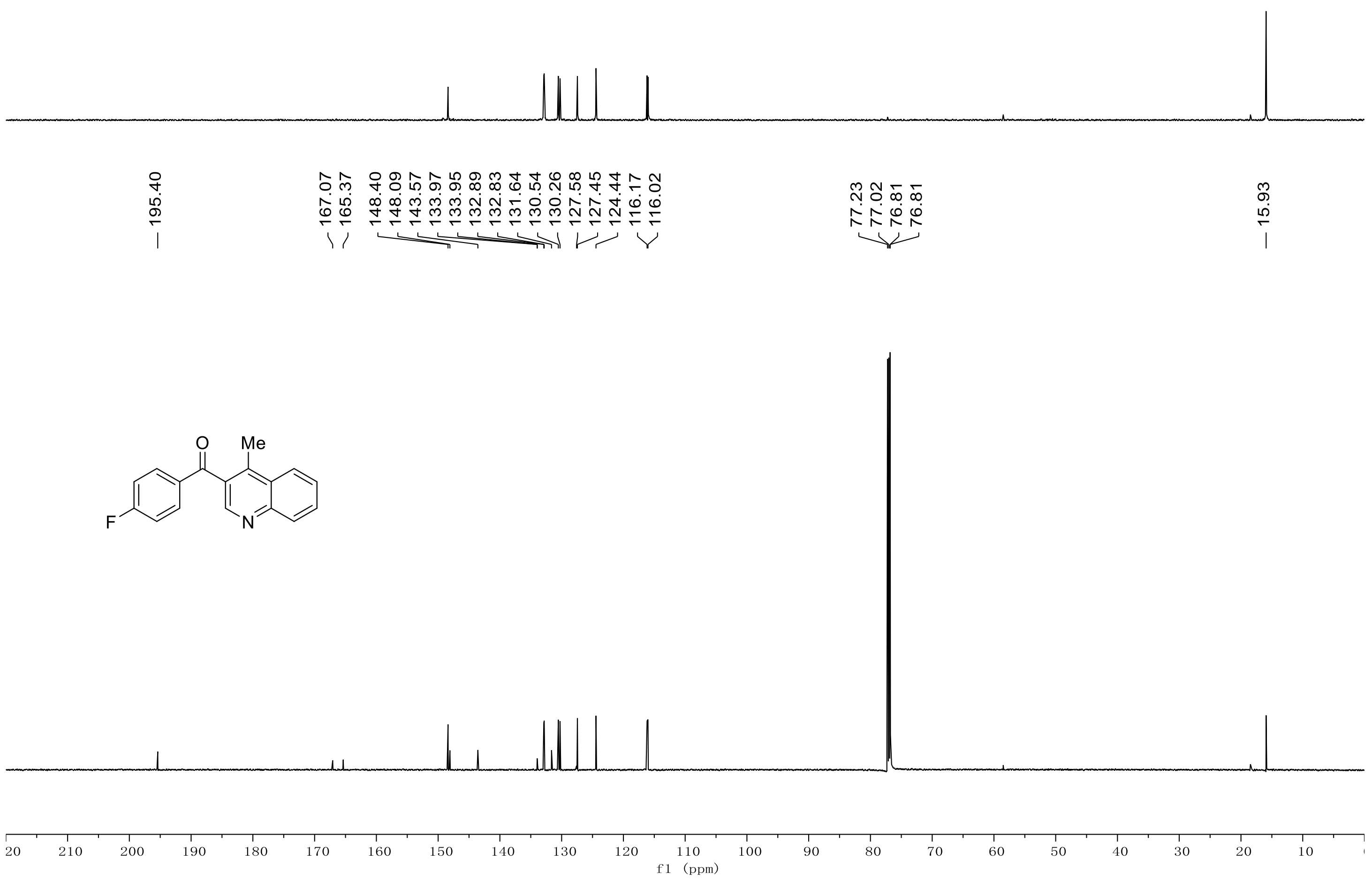

Figure 53. ^{13}C NMR (150 MHz, $CDCl_3$) spectra of compound **3z**

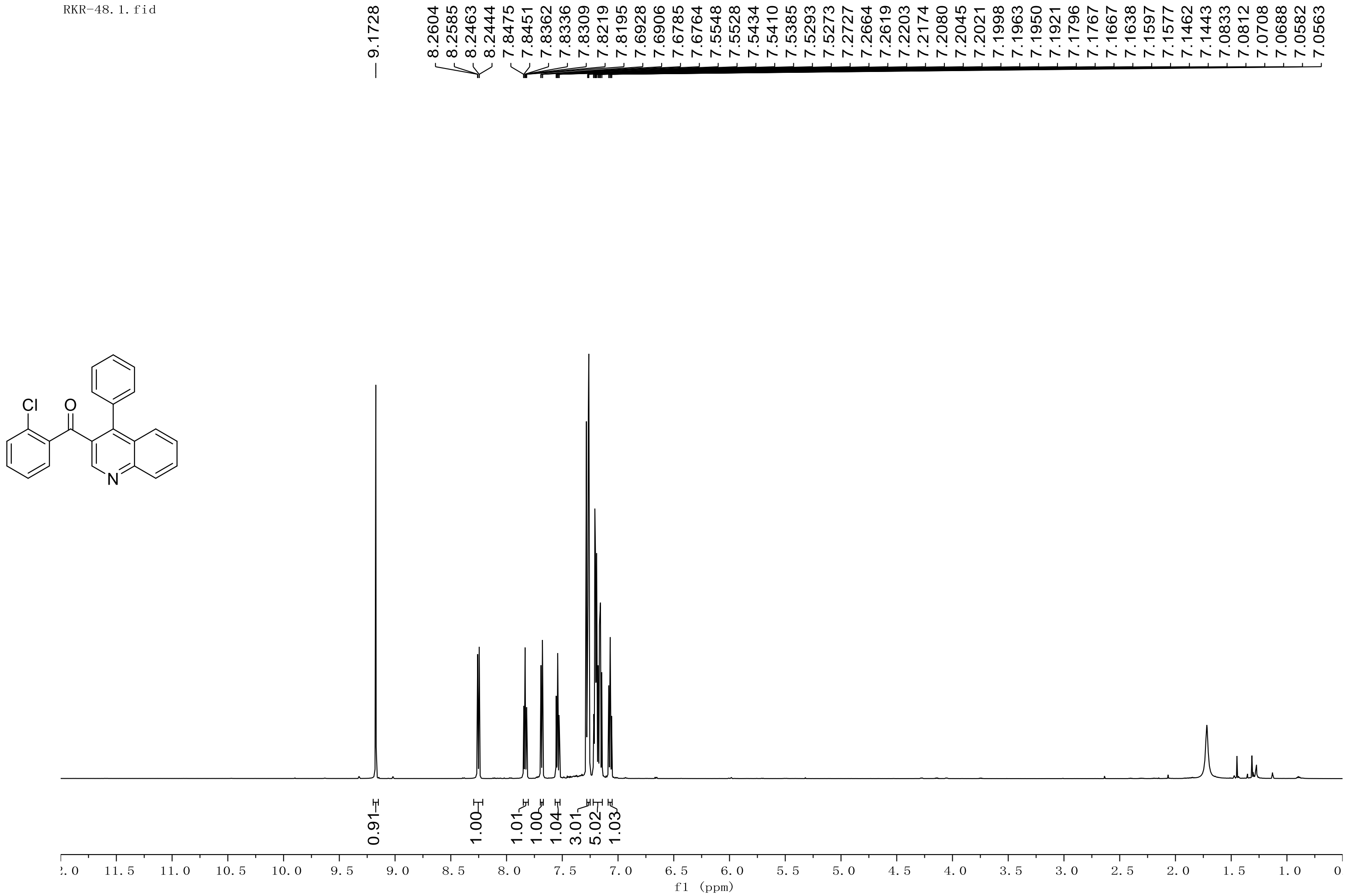

Figure 54. ^{1}H NMR (600 MHz, $CDCl_3$) spectra of compound **3a'**

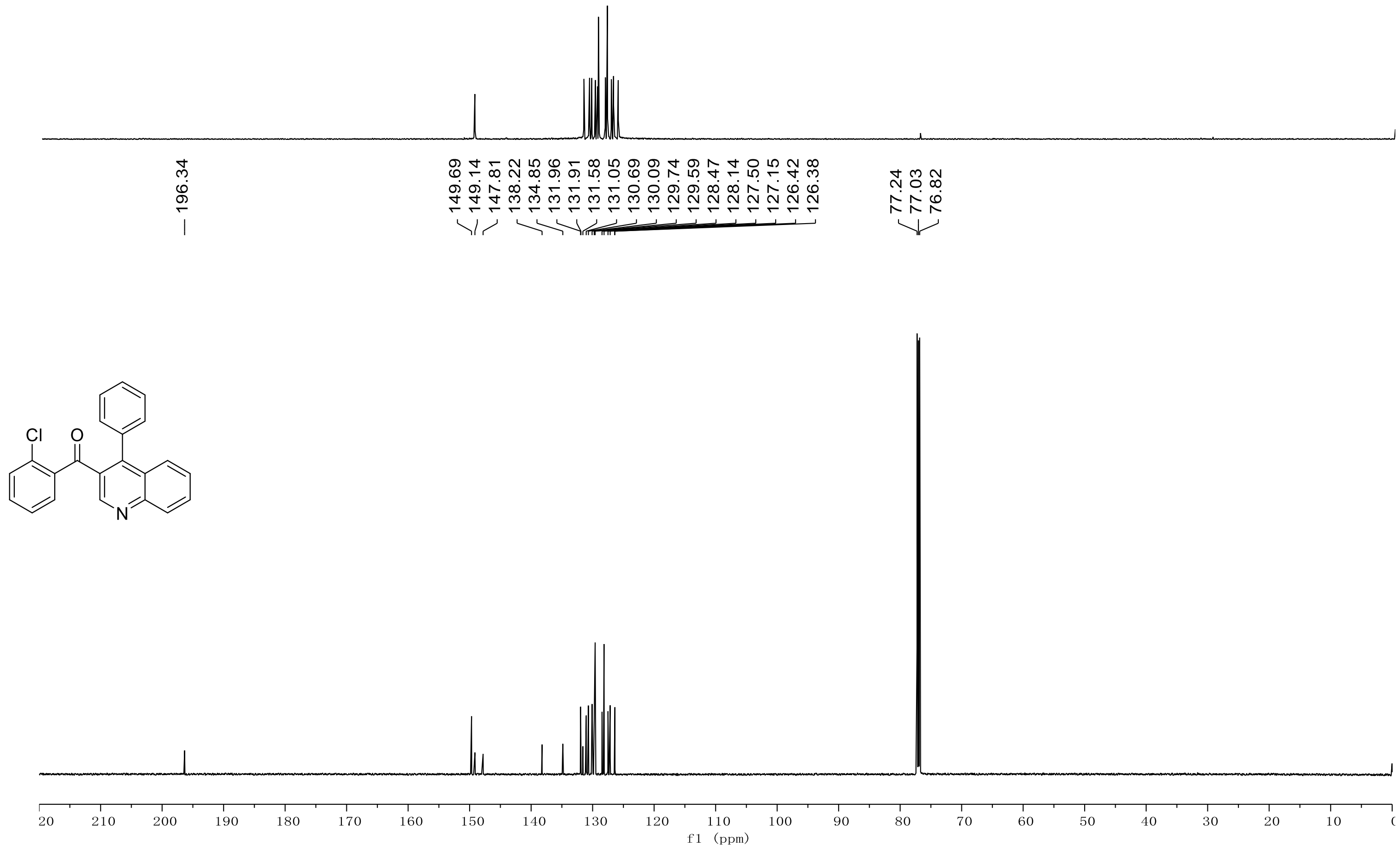

Figure 55. ^{13}C NMR (150 MHz, $CDCl_3$) spectra of compound **3a’**

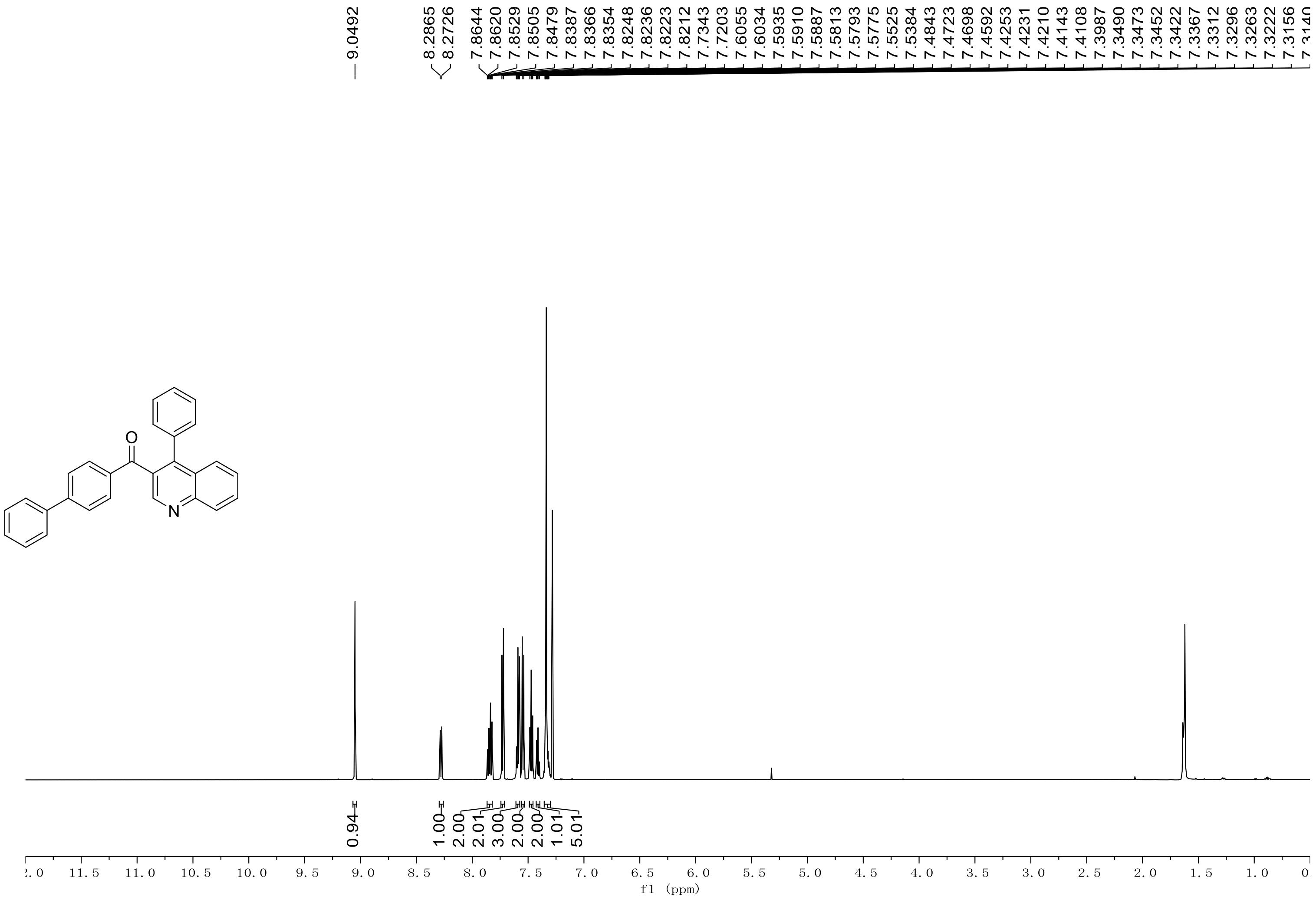

Figure 56. ^{1}H NMR (600 MHz, $CDCl_3$) spectra of compound **3b'**

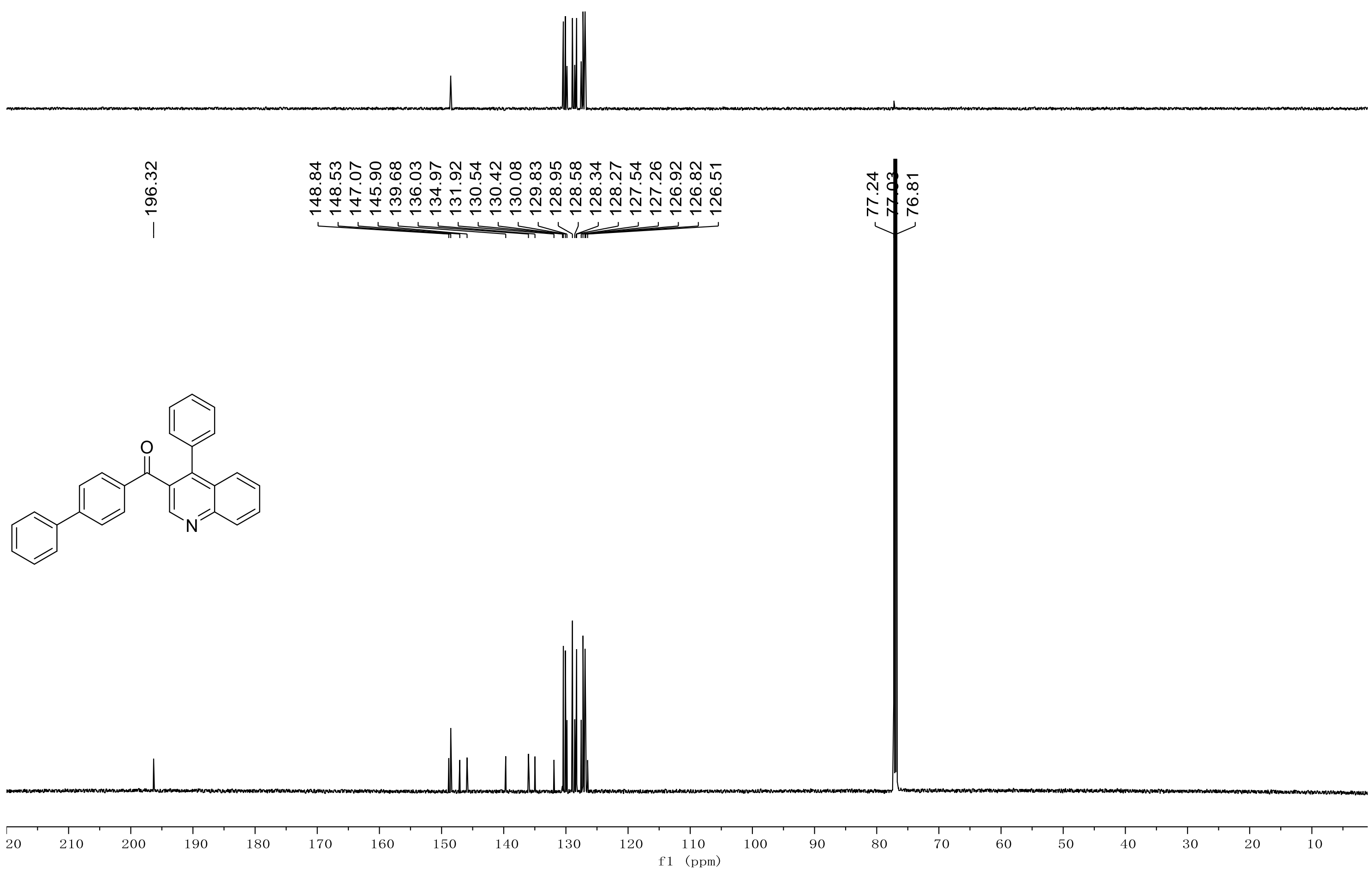

Figure 57. ^{13}C NMR (150 MHz, $CDCl_3$) spectra of compound **3b'**

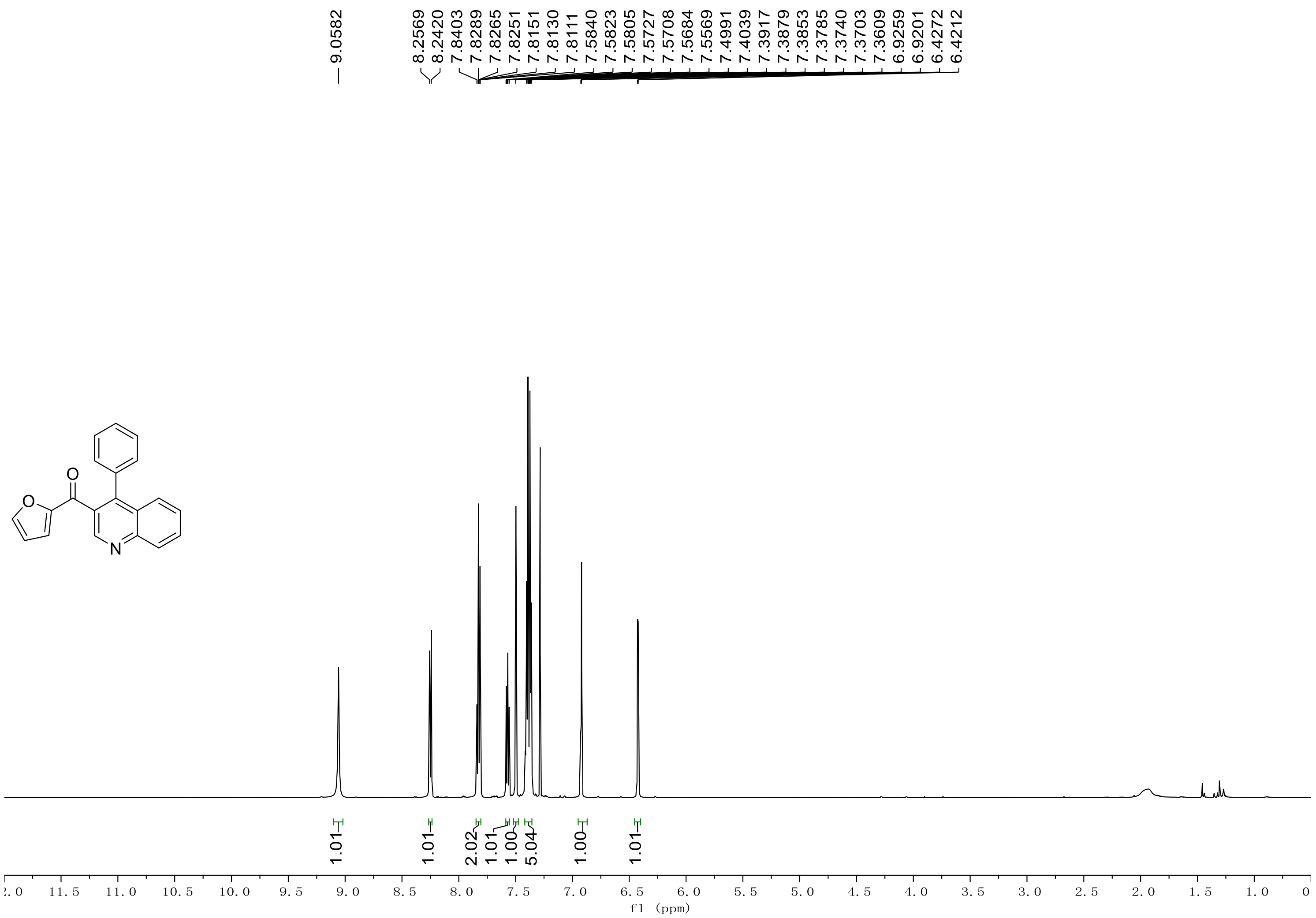

Figure 58. ^{1}H NMR (600 MHz, $CDCl_3$) spectra of compound **3c’**

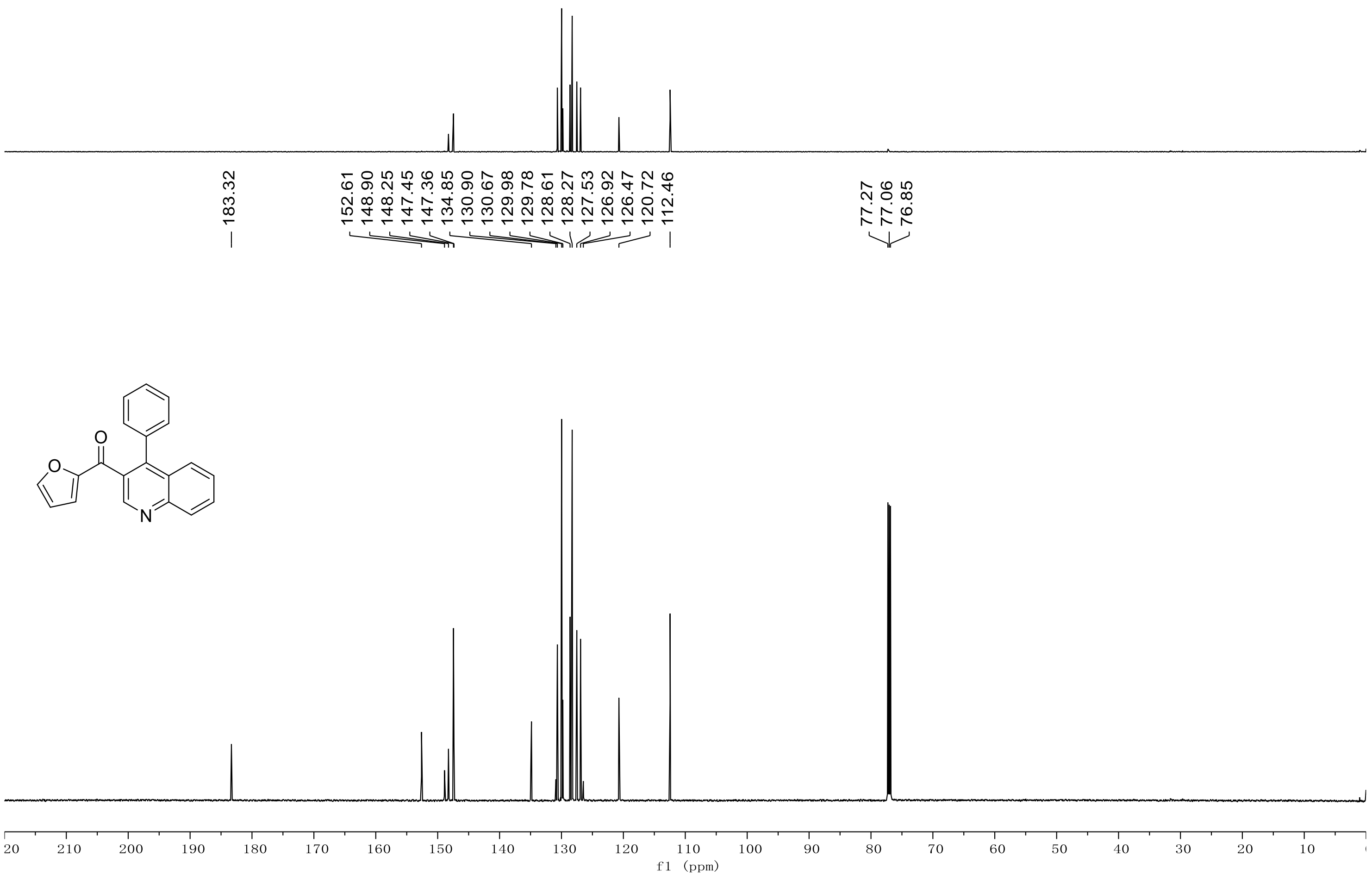

Figure 59. ^{13}C NMR (150 MHz, $CDCl_3$) spectra of compound **3c'**

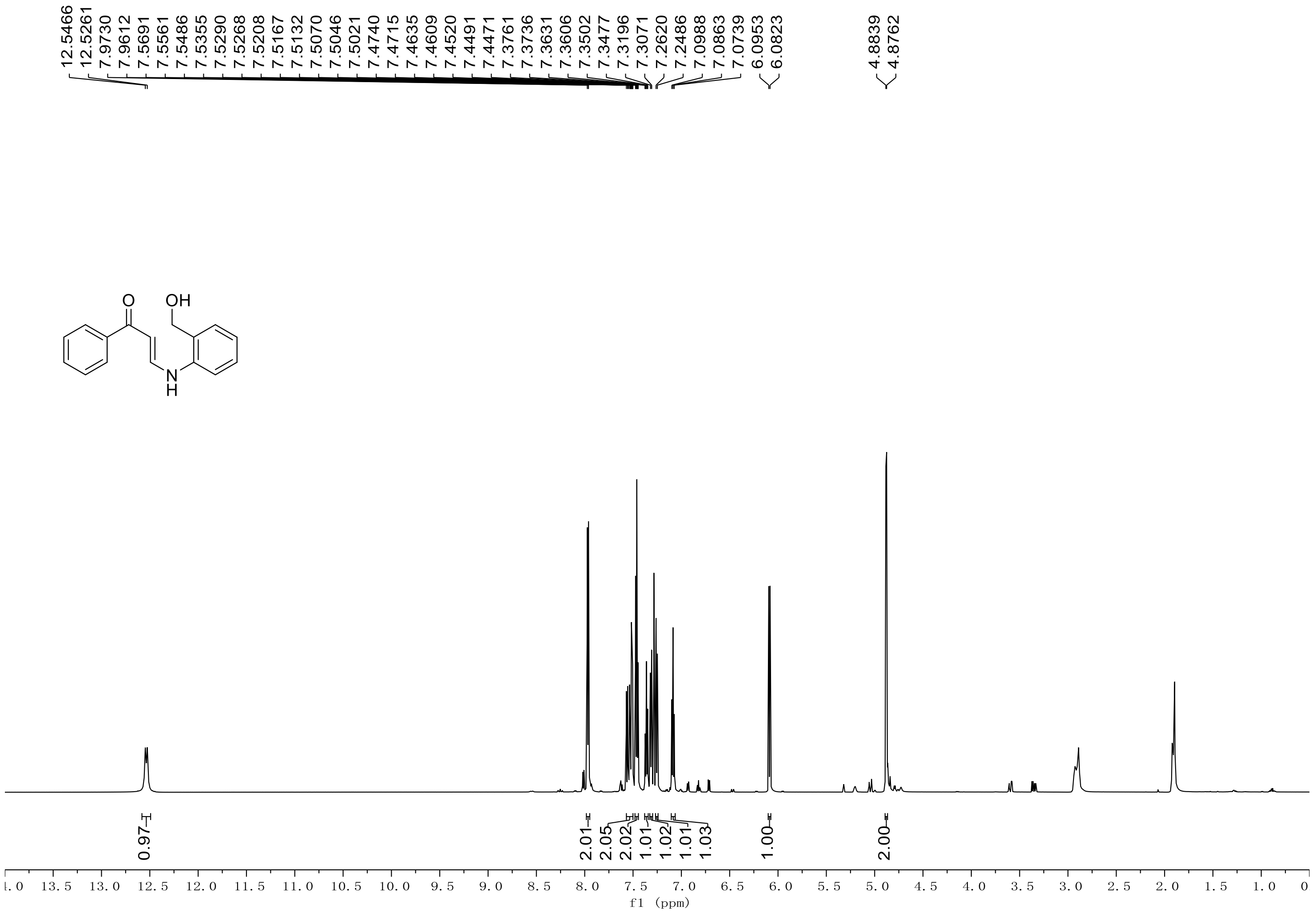

Figure 60. ^{1}H NMR (600 MHz, $CDCl_3$) spectra of compound **4**

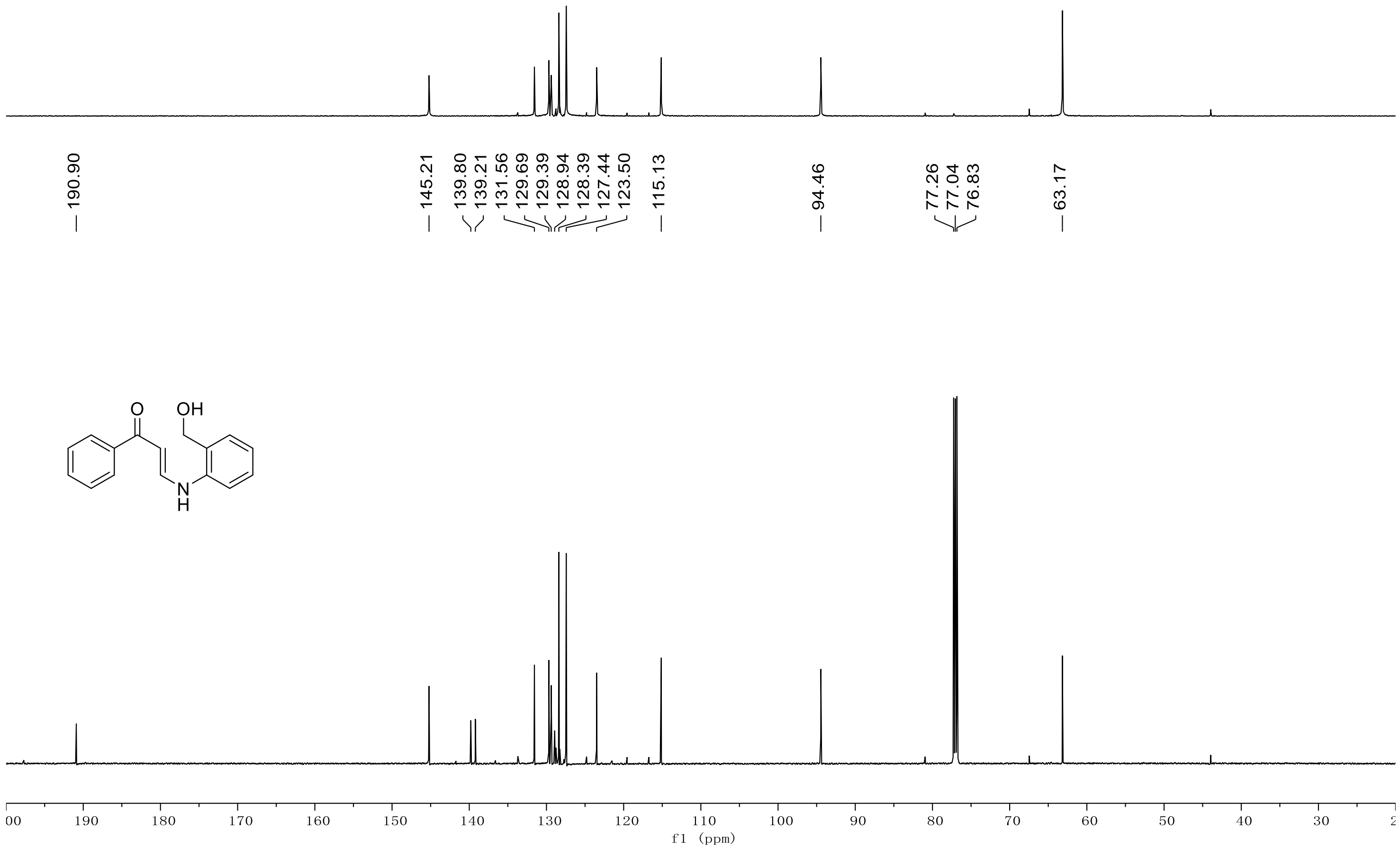

Figure 61. ^{13}C NMR (150 MHz, $CDCl_3$) spectra of compound **4**

7. References and notes.

1. (*a*) L. E. Kiss, H. S. Ferreira, L. Torrão, M. J. Bonifácio, P. N. Palma, P. Soares-da-Silva and D. A. Learmonth. *J. Med. Chem.* 2010, **53**, 3396; (*b*) S. Kantevari, S. R. Patpi, D. Addla, S. R. Putapatri, B. Sridhar, P. Yogeeswari and D. Sriram. *ACS Comb. Sci.* 2011, **13**, 427; (*c*) S. Hernández, I. Moreno, R. SanMartin, G. Gómez, M. T. Herrero and E. Domınguez. *J. Org. Chem.* 2010, **75**, 434.
2. CCDC 1846910 contain the supplementary crystallographic data for compound **3j**. These data can be obtained free of charge from The Cambridge Crystallographic Data Center *via* www.ccdc.cam.ac.uk/data_request/cif.
